# Pan-cancer landscape of AID-related mutations, composite mutations, and their potential role in the ICI response

**DOI:** 10.1038/s41698-022-00331-2

**Published:** 2022-12-01

**Authors:** Isaias Hernández-Verdin, Kadir C. Akdemir, Daniele Ramazzotti, Giulio Caravagna, Karim Labreche, Karima Mokhtari, Khê Hoang-Xuan, Matthieu Peyre, Franck Bielle, Mehdi Touat, Ahmed Idbaih, Alex Duval, Marc Sanson, Agustí Alentorn

**Affiliations:** 1 Sorbonne Université, Inserm, CNRS, UMR S 1127, Institut du Cerveau et de la Moelle épinière, ICM, Paris, France; 2 Departments of Genomic Medicine and Neurosurgery, University of Texas MD Anderson Cancer Center, Houston, TX USA; 3 Department of Medicine and Surgery, University of Milano-Bicocca, Milano, Italy; 4 Cancer Data Science Laboratory, Dipartimento di Matematica e Geoscienze, Università degli Studi di Trieste, Trieste, Italy; 5 Department of Neuropathology, Pitié Salpêtrière-Charles Foix, Paris, France; 6 Onconeurotek, AP-HP, Hôpital Pitié-Salpêtrière, F-75013 Paris, France; 7 Department of Neurology-2, Pitié-Salpêtrière University Hospital, Assistance Publique-Hôpitaux de Paris (AP-HP), Paris, France; 8 Department of Neurosurgery, AP-HP, Hôpital Pitié-Salpêtrière, F-75013 Paris, France; 9 Sorbonne Université, INSERM, Unité Mixte de Recherche Scientifique 938 and SIRIC CURAMUS, Centre de Recherche Saint-Antoine, Equipe Instabilité des Microsatellites et Cancer, Equipe labellisée par la Ligue Nationale contre le Cancer, F-75012 Paris, France; 10 Sorbonne Université, Genetics Department, AP-HP.Sorbonne Université, hospital Pitié-Salpêtrière, F-75012 Paris, France

**Keywords:** Translational research, Cancer genomics

## Abstract

Activation-induced cytidine deaminase, *AICDA* or AID, is a driver of somatic hypermutation and class-switch recombination in immunoglobulins. In addition, this deaminase belonging to the APOBEC family may have off-target effects genome-wide, but its effects at pan-cancer level are not well elucidated. Here, we used different pan-cancer datasets, totaling more than 50,000 samples analyzed by whole-genome, whole-exome, or targeted sequencing. AID mutations are present at pan-cancer level with higher frequency in hematological cancers and higher presence at transcriptionally active TAD domains. AID synergizes initial hotspot mutations by a second composite mutation. AID mutational load was found to be independently associated with a favorable outcome in immune-checkpoint inhibitors (ICI) treated patients across cancers after analyzing 2000 samples. Finally, we found that AID-related neoepitopes, resulting from mutations at more frequent hotspots if compared to other mutational signatures, enhance *CXCL13*/*CCR5* expression, immunogenicity, and T-cell exhaustion, which may increase ICI sensitivity.

## Introduction

**Fig. 1:**
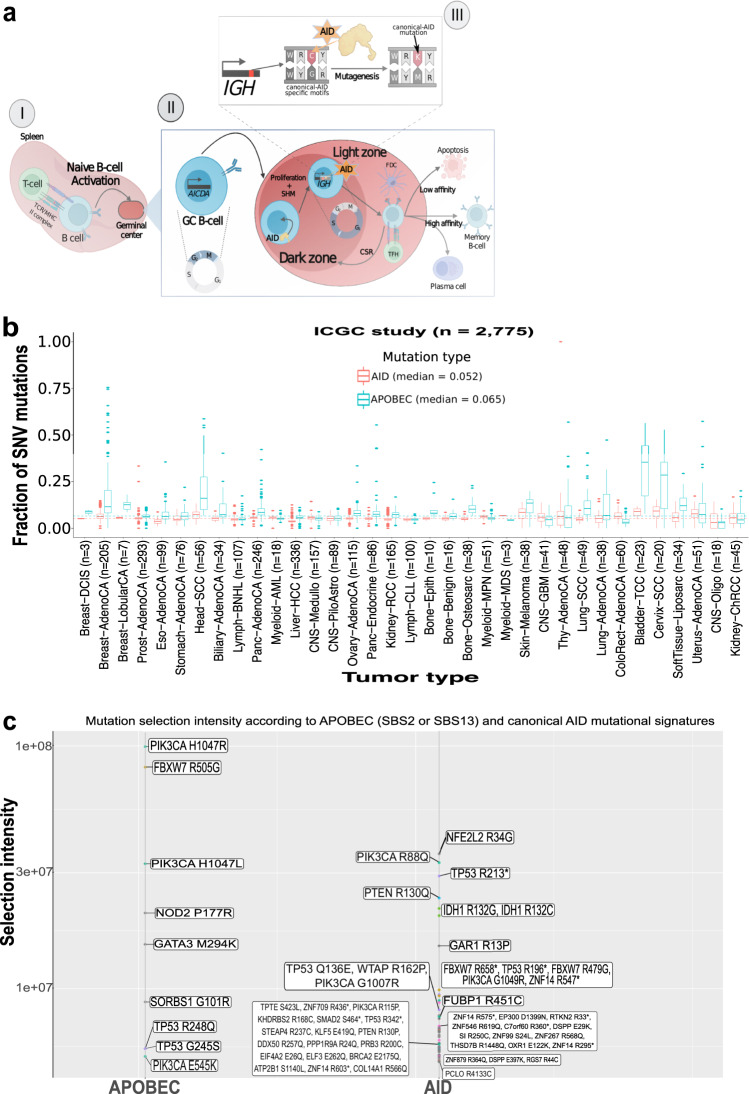
Pan-cancer landscape of AID-related mutations. **a** Illustrative representation of *AICDA* expression and AID activity within normal B cells. For AID motifs, W = A/T; R = Purine; Y = Pyrimidine; K = G/T; M = C/A. **b** Frequency of the fraction of SNV mutations attributed to AID motifs or APOBEC motifs for each tumor type in the ICGC cohort; dotted lines indicate median values across all samples. Center line represents the median values; error bars represent the upper and lower quartiles and whiskers define the minimal and maximum values. **c** AID mutations produce higher selection intensity on driver genes on minor hotspot residues but there is a higher number of affected genes/residues than the ones generated by the APOBEC related signatures.

Naive B cells enter the germinal centers (GC) of secondary lymphoid organs after being activated by a cognate antigen, where they induce the production of Activation-induced cytidine deaminase (AICDA), especially during the G2-M phases of the cell cycle (Fig. 1a, I).

AID (encoded by *AICDA*) is involved in the diversification of the variable (V) or switch domains of immunoglobulin (IG) genes during the G1-S phases of the cell cycle. It is responsible for somatic hypermutation in the dark zone of the GC and class-switch recombination in the light zone (Fig. 1a, II)^1–3^. AID deamination of cytosine to uracil also occurs during IG gene transcription and inside particular DNA patterns (Fig. 1a, III)^4^. Mutations can arise as A- > C at WA motifs (W = A/T) when resolved by the error-prone DNA polymerase-eta, which has been defined as non-canonical AID (COSMIC signature 9), or as C- > T/G at WRCY motifs (R = purine; Y = pyrimidine) when resolved by base excision repair or mismatch repair pathways, which has been defined as canonical-AID (c-AID, Fig. 1a, III)^5^. Although the single-base substitution (SBS) COSMIC somatic signatures SBS84 and SBS85 (v3.2) have been recently associated to c-AID activity, they were discovered in a trinucleotide context (specifically at RCY motifs), which does not always correspond to the observed tetranucleotide context in which c-AID acts (WRCY motifs)^6–8^. Furthermore, AID belongs to the same enzyme family as APOBEC3A and APOBEC3B, which are known to be a source of somatic mutations in a variety of malignancies and are designated by the SBS2 and SBS13 signatures according to Alexandrov, but unlike c-AID, act in trinucleotide context (TCW motifs)^6,9,10^. Off-target AID activity has also been reported in lymphomas and other hematological cancers^11,12^, but only in a few solid tumors^11–16^. Despite this, no detailed characterization of the involvement of AID-related mutations at the pan-cancer level, as well as their potential mutational and clinical implications, has been performed. The c-AID mutations were then characterized across 49 thousand tumoral samples (9 human cohorts and 3 non-human cohorts, see Supplementary methods), revealing that: (i) they are found at a frequency of 5.2% (5.1–5.3%) in virtually all cancers (human and non-human); (ii) they show stronger activity at transcriptionally active domains; and (iii) they synergize initial non-AID hotspot mutations by a second c-AID composite mutation.

Additionally, since the APOBEC mutational signature (SBS2 and SBS13) has been proposed as a biomarker for ICI response in some cancers^17,18^, we used more than 2000 ICI-treated samples^19–21^, finding AID-related fraction of mutations as an independent prognostic value to ICI after adjusting by tumor mutational burden (TMB) and APOBEC signature.

Overall, we used more than 50.000 samples covering more than 80 tumor types to thoroughly describe the landscape of AID-related mutations (Supplementary Tables 1–2).

## Results

### Landscape of AID-related mutations at pan-cancer level

We identified the mutations induced by c-AID activity by tracking the C to G/T mutations within its specific WRCY motifs. We discovered AID-related mutations in the great majority of malignancies investigated while evaluating the PCAWG data (ICGC; 2775 cancer patients and 35 cancer types). Overall, AID-related mutations were detected in 5.2% (5.1–5.3% at 95% confidence interval [CI]), while APOBEC mutations (SBS2 + SBS13) were found in 6.5% (6.4–6.7% at 95% CI; Fig. 1b) of all single-nucleotide variants (SNV) mutations. Using the TCGA, MSKCC cohorts, and various pediatric datasets, we observed similar results at the pan-cancer level (Supplementary Fig. 1 and Supplementary Fig. 2). Conversely, as expected, the frequency of AID-related mutations was slightly higher in hematological cancers at ~8%, specially for B-cell malignancies, like diffuse large B-cell lymphoma (DLBCL), which showed a 10.9% frequency (Supplementary Fig. 2). Intriguingly, the AID mutations were also identified in canine melanoma, glioma, and osteosarcoma at a frequency of 6.0%, 4.7%, and 2.9%, respectively (Supplementary Fig. 2). However, c-AID signatures were less abundant than other tumor-specific major signatures, for example the ultraviolet (UV) exposure related signatures (SBS7a and SBS7b) in skin melanoma had a frequency of 65.0% (26.0 + 39.0%) versus 7.9% of c-AID; the tobacco smoking signature (SBS4) in Lung adenocarcinoma with a 50.0% frequency versus 4.2% of c-AID; and the temozolomide-related signature (SBS11) in glioblastoma (GBM) with 25.2% versus 7.7% of c-AID (Supplementary Fig. 1). Moreover, to discard an association of our tetranucleotide-based c-AID mutations (using ICGC cohort) with other COSMIC somatic signatures (v3.2) we computed the cosine similarity scores and observed SBS84, SBS9, and SBS85 showing low cosine scores of 0.497, 0.157, and 0.039, respectively. Cosine similarity is a measure of closeness between two mutational profiles where a value of one represents identical signatures while of zero completely different ones. Alexandrov et al. and others have demonstrated two mutational signatures to be different when having a cosine similarity score inferior to 0.75^6,8,22,23^. Next, to discard that the observed c-AID mutations are due to randomness, we generated a background mutational model by simulating, for each sample from the ICGC cohort, its mutations 1000 times. We preserved, within each chromosome, the mutational burden and mutational patterns at pentanucleotide resolution (mutated base-pair with ± 2 bp context) to generate a distribution of mutations and a null hypothesis about the number of c-AID-related mutations generated by chance (globally, per tumor type, and per sample). Pentanucleotide resolution, in which c-AID motifs fall, was chosen as it has been previously demonstrated to more completely capture the patterns of substitution mutational signatures in human cancer^23^. We observed a 2.69 (2.67–2.71 at 95% CI) enrichment of observed versus expected c-AID-related mutations globally, and only 3/2727 samples having significantly more c-AID mutations by chance (two-sided Fisher exact test; Supplementary Fig. 3). We removed those three samples from further analyses. These observations indicate that it is very unlikely that the majority of the observed c-AID mutations are the result of chance or an already reported mutational signature.

Concerning the genomic distribution of AID motifs in the normal genome, the quantity is not different across chromosomes when adjusting the motifs’ number by chromosome length (FDR corrected *p*-value Wilcoxon test; Supplementary Fig. 4). Regarding AID-related mutations, in DLBCL most commonly affected chromosomes involved the presence of either immunoglobulin related genes: *IGH* (chr14), *IGL* (chr22), *IGK* (chr2), or genes already related with off-target AID activity: *PIM1*, *IRF4*, *HIST1H1C* (chr6; Supplementary Fig. 4)^24^. Globally speaking, for the majority of tumor types the highest density of AID mutations were located in chromosome 5, in which *GPR98* and *DNAH5* were frequently affected, followed by chromosomes 17 and 2 (Supplementary Figs 5–8).

Interestingly, within the driver genes context, hematological cancers (i.e., non-Hodgkin’s lymphoma (Lymph-BNHL), DLBCL) and MB had the highest signature contribution of AID provoked mutations. Furthermore, among the involved targets, *TP53*, in all cohorts; *IDH1*, in hematological cancers, GBM and low-grade glioma (LGG); and *PIK3* genes (TCGA and ICGC cohorts), were recurrently altered (Supplementary Fig. 9). These results were also confirmed using a selection intensity approach, i.e., of every somatic mutation within the ICGC dataset. Selection intensity, introduced by Cannataro et al., is defined as a metric of proliferative advantage of a specific mutation under a model of somatic selection^25^. We found a higher selection intensity of *PIK3CA*, *NFE2L2* but also in “minor” *IDH1* mutations (i.e., not R132H) and *PTEN* (Fig. 1c).

*AICDA* expression and AID-related mutations were not correlated, and only in thyroid cancer (THCA) were slightly positively correlated (Rho = 0.18, *p*_adj_ = 0.01), suggesting that *AICDA* is not constitutively activated in any cancer. The AID mutations were more frequently negatively correlated with the TMB of cancers from TCGA (i.e., in adenoid cystic carcinoma (ACC), kidney renal papillary cell carcinoma (KIRP), kidney renal clear cell carcinoma (KIRC), liver hepatocellular carcinoma (LIHC), lung adenocarcinoma (LUAD), ovarian cancer (OV), and THCA; Supplementary Fig. 10). In brief, we found AID activity leaves important DNA footprints across human and not-human tumors, including driver genes.

### Oncogenic AID activity is higher at transcriptionally active domains and differs according to transcription direction

AID activity within the normal context takes place especially during transcription elongation, when the polymerase becomes stalled, and requires a licensing step to regulate over-activity which can be bypassed when abnormal high nuclear levels of AID are present. On the other hand, R-loops, a hybrid structure of B-form double-stranded DNA and A-form dsRNA, are formed during transcription which increases DNA exposure and has been linked to AID activity^26,27^. By using genomic coordinates of R-loop associated regions and the ICGC cohort (WGS data), we attempted to answer if, within the tumoral context, AID mutations were more localized in or out these regions compared to either APOBEC mutations (COSMIC SBS2/SBS13), other mutational processes, or c-AID mutations expected by chance (from the simulated data). The number of expected c-AID mutations falling “in R-loops” from simulated data was significantly lower than those c-AID mutations observed in real data (0.13% vs 0.18%, *p*-value <0.001), which is probably the consequence of having globally less c-AID mutations by chance (Supplementary Fig. 3). However, within the real data the c-AID falling “in R-loops” (1130/629,871) was not significantly different to those caused by SBS2 (0.19%; 456/241,695; *p*-value = 0.37; two-sided Fisher exact test) or SBS13 (0.19%; 400/204,922; *p*-value = 0.15) (Supplementary Table 3). Overall, this suggests that within the oncogenic context, AID promiscuous activity is not particularly related to R-loop formation.

**Fig. 2:**
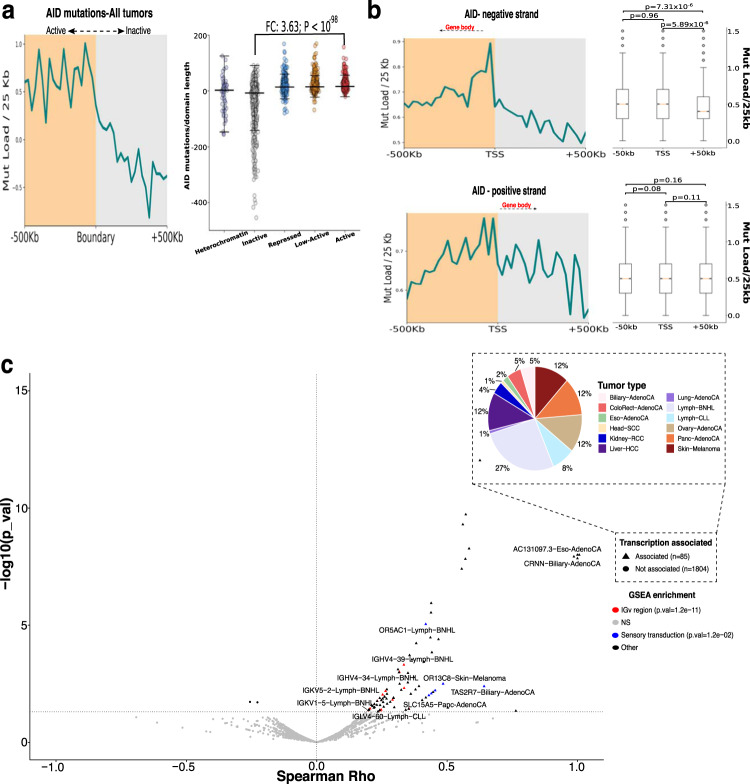
Oncogenic AID activity is higher at transcriptionally active domains and differs according to transcription direction. **a** Average profile of AID somatic mutations accumulation in 2775 cancer samples and replication timing across 500 kb of TAD boundaries delineating active to inactive domains (left); dot plots representing the distribution of the mutations divided by the domain length in different domain-types (heterochromatine = purple, inactive = gray, repressed = blue, low-active = orange, active = red; right; Wilcoxon rank-sum test). Error bar limits are the 25th and 75th percentiles; the center line is the median. **b** Average profiles of c-AID mutations accumulation in 2775 cancer samples across 500 kb of TSS for negative-strand genes (top) or positive-strand genes (bottom). Boxplots on the right shows the mutational load comparisons within the TSS and adjacent ∓50 bins on each strand where error bar limits are the 25th and 75th percentiles; the center line is the median and whiskers define the minimal and maximum values. **c** Volcano plot (*n* = 1130) showing the genes whose expression and mutations are correlated per tumor type (p-adj < 0.05, Spearman Rho > 0), where colors indicate genes enriched in a specific pathway by DAVID database analysis and the pie chart (inset plot) indicates the distribution across tumor types of the associated genes. All panels were produced using the ICGC cohort.

Next, we used topologically associated domains (TAD) information of five different previously defined domains (i.e. heterochromatine, inactive, repressed, low-active, and active), to see the distribution of AID/APOBEC mutations across chromatin folding domains^28–30^. We found AID mutations occurring more towards active domains than inactive (FC = 3.63; *p*-val = 5.01 × 10^−98^), especially at the TADs boundaries (Fig. 2a-left). As previously described, we found that APOBEC signatures are also causing mutations towards active domains but the active/inactive ratio is notably higher for the SBS13 than the SBS2, indicating distinct molecular underpinnings (Supplementary Fig. 11).

Since the R-loop forming regions do not cover all the transcription start sites (TSS) and given the observed association with active domains, we next analyzed the AID mutation’s distribution around the TSS as previous studies showed recruitment of AID to those sites^27^. By dividing mutated genes based on strandness, we found a very particular pattern for the AID mutations falling on the negative strand compared to the positive strand. Mutations accumulate near the TSS and towards the gene body while maintaining a more constant and significantly higher mutational load compared to the opposite direction (Fig. 2b; −50 kb vs TSS, *p*-value: 0.96; TSS vs +50 kb, *p*-value: 5.89 × 10^−06^). This was not observed on the positive strand (−50 kb vs TSS, *p*-value: 0.08; TSS vs +50 kb, *p*-value: 0.11) nor on the APOBEC signatures at either strand (Supplementary Fig. 12).

Next, we wondered if the AID mutations of a specific gene were produced during transcription of that same gene. To answer this we used 1130 samples (comprising 24 tumor types) from which the mutations and expression data were available (ICGC cohort) and correlated the number of c-AID mutations occurring in gene i (AID_Muts_gi_) to the expression of the same gene i (Exp_gi_) within each tumor type since the expression and mutations vary greatly in this context. Only 6.6% of the total different mutated genes (21,341) were expressed from which 6.0% (81/1,403, not repeated genes) were correlated with its corresponding gene expression per tumor type (p.adj <0.05, Spearman Rho > 0); additionally, more than one-third of these genes were found in hematological cancers (Lymph-BNHL and Lymph-CLL). Gene set enrichment analysis revealed immunoglobulin V region-related genes (adjusted *p*-value = 1.2 × 10^−11^) within hematological cancers (Fig. 2c). Other interesting genes included, *CRNN* in biliary adenocarcinoma, which loss of expression had been previously associated with poor survival in esophageal squamous cell carcinoma^31^, and the transporter-associated gene, *SLC15A5* in pancreatic adenocarcinoma. Altogether, our analysis suggests that AID activity is coupled to the transcription process with immunoglobulin genes in hematological cancers following the line of “normal” context as the expression is more constitutive. However, the mutations in other genes are probably produced during short-term transcription of both the affected gene and *AICDA* whose dynamics depend on the strand location of the gene and hence the direction of transcription.

### The impact of AID-related mutations with ICI response

**Fig. 3:**
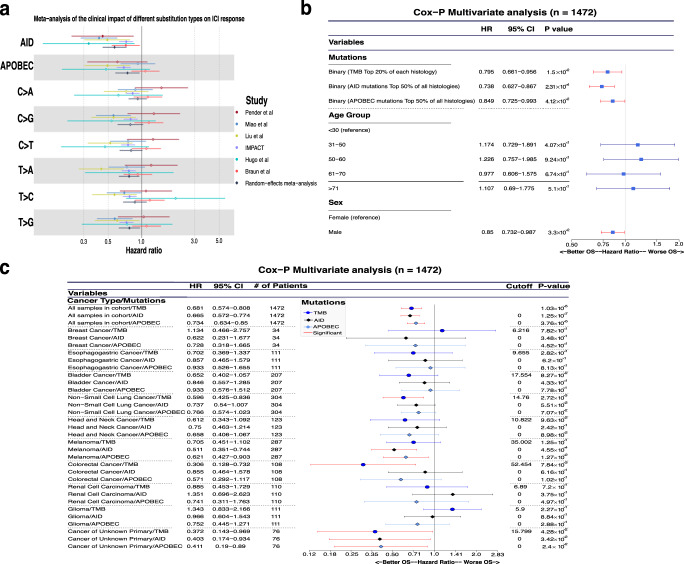
The impact of AID mutations on ICI response. Meta-analysis of the survival impact of the fraction of AID mutations in different studies. **a** Effect of using AID/APOBEC (5th decile as cutoff) or SNV substitutions where AID remains significant across all the studies. **b** Forest plot of a Cox model of the global impact, after adjustment by TMB (top 20%), median APOBEC mutations, age, and gender. **c** Forest plot of the Cox model of the impact of AID mutations per cancer subtype.

Because several recent studies pinpointed a potential role of APOBEC related mutations on the efficacy of ICI^17,18^, we sought to use the fraction of AID as a surrogate marker of ICI response. We used different open access datasets that had available genomic and clinical data (see Methods). We performed a random-effects meta-analysis comparing the overall survival (OS) of all these studies and comparing the impact of AID, to the APOBEC signature and the different SNV. The details of this analysis are provided in the methods. Strikingly, the AID-related mutations were associated with the best OS in all of the studies and the random-effects model showed also a favorable prognosis (median as the cutoff, Fig. 3a). Moreover, the effect was still significant across almost all the studies independently of either decile chosen as cutoff at univariate (Supplementary Fig. 13) or multivariate adjusting for TMB (Supplementary Fig. 13). Accordingly, the APOBEC signature was associated with a favorable prognosis, but not in all datasets. However, the random-effects model also indicated an overall favorable prognosis associated with APOBEC. The rest of SNV showed much more heterogeneous results and only T > A and T > G mutations were associated with favorable prognosis in the random-effects model (Fig. 3a).

Interestingly, within the largest study of IMPACT-MSKCC, the fraction of AID-related mutations (top 50% of all histologies as the cutoff) was also independently associated with both better OS (Hazard ratio [HR] = 0.715; 95% CI = 0.61–0.839; *p* = 3.81 × 10^−5^) and predictive value compared to TMB or APOBEC after adjusting by TMB (top 20% of each histology as the cutoff), APOBEC signature (top 50% of all histologies as the cutoff) age and sex (Fig. 3b). It should be noted, that when using a univariate Cox proportional Hazards ratio model per every cancer type or adjusting by TMB ≥ 10, the results were also similar in the overall population of this study, but the clinical impact of AID-related fraction of mutations was only found in metastatic melanoma and cancer with unknown primary (Fig. 3c; Supplementary Fig. 13). Additionally, there was practically no correlation between the fraction of AID mutations with the APOBEC signature neither globally nor by tumor type in this cohort and in the ICGC and TCGA datasets (Supplementary Fig. 14). Similarly, by using four additional studies across different tumor types, we also found an association of high AID mutations with improved OS after adjusting by age, gender, and TMB using the multivariate Cox model^19,20,32,33^.

Overall, all the studies confirmed the independent prognostic value of the high fraction of AID mutations according to the median in the univariate and multivariate analyses.

### Landscape of AID-related neoepitopes and its relation with ICI response

**Fig. 4:**
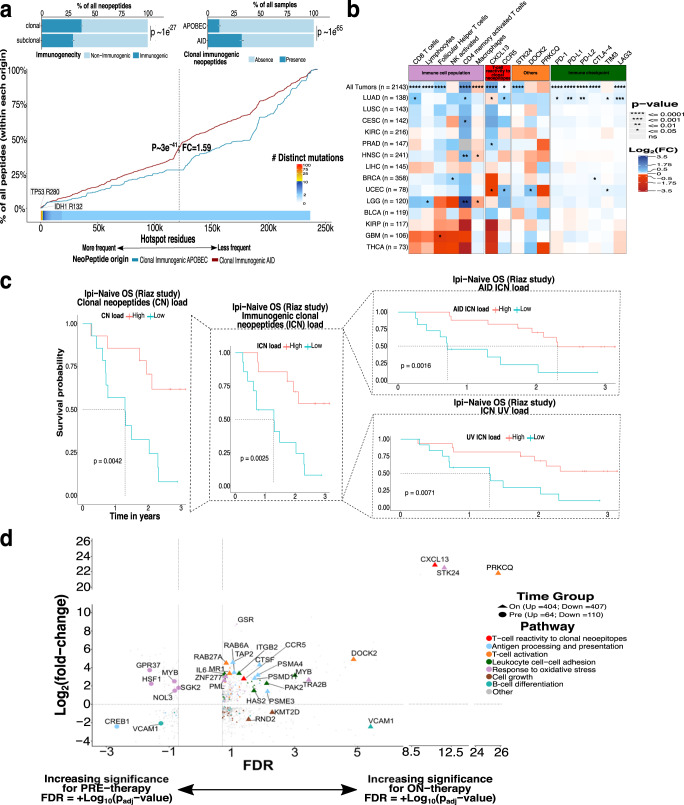
Landscape of AID-related neoepitopes and its relation with ICI response. **a** Percentage of neoepitopes originating from clonal/subclonal mutations in which the color indicates comparison for immunogenic or non-immunogenic calculated by Prime (top left; Two-sided two-sample Z-test for equal proportions). Top right shows the comparison of the percentage of samples having at least one AID immunogenic clonal neoepitopes (ICN) versus APOBEC ICN (“Presence”; Two-sided two-sample Z-test for equal proportions). Bottom plot shows the cumulative distribution of hotspot mutation utilization among the AID/APOBEC ICNs as a function of decreasing population-level frequency (Two-sided Mann–Whitney U test, FC of median AID to APOBEC values, error bars are 95% binomial confidence intervals). The number of distinct mutations indicates the number of unique samples having a mutation at a specific residue (e.g., IDH1 R132). **b** Heatmap of gene expression comparison between AID ICN “Presence” versus “Absence” groups across tumor types/all tumors (*n* = 2143; two-sided Wilcoxon test) measured as log2 FC. **c** OS prediction within Ipi-Naive patients improves when using AID ICN load (top right), ICN UV load (bottom right), ICN load (middle), or clonal neoepitopes load (left), lowest to highest log-rank *p*-values. **d** DEGs (p-adj < 0.20) between high ICN load patients versus low ICN load for pre-therapy, where increasing negative values on the *x*-axis shows higher significance (+Log10[p-adj]), or on-therapy, where increasing positive values on the *x*-axis means higher significance (−Log10[p-adj]). The *y*-axis shows upregulated (FC > 0) or downregulated genes (FC < 0) and colors indicate genes enriched in a specific pathway by GO analysis. **a** and **b** correspond to the TCGA cohort (*n* = 2143) meanwhile **c** and **d** to ICI-treated melanoma cohort (Riaz et al., *n* = 68).

Having found an association between AID activity and ICI benefit, we hypothesized that AID mutations might generate highly immunogenic neoepitopes. We addressed this by analyzing the neoepitopes that were products of AID activity on the TCGA cohort and on melanoma patients treated with Nivolumab (anti-PD-1)^34^. A recent bioinformatic-experimental study using immunogenic and non-immunogenic peptides, experimental testing, and X-ray structures showed that TCR binding and recognition improves with the presence of hydrophobic amino acids (aromatic W, F, Y followed by V, L, and I) at specific “MIA” positions (position P_4_-P_Ω−1_) due to increased structural avidity, stacking interactions, hydrogen bond acceptance and limited rotational freedom with the TCR^35^. Additionally, as a previous study showed that APOBEC promiscuous activity increases neopeptide hydrophobicity^36^, we wondered if AID-related mutations led to the production of not only more hydrophobic neoepitope but more “Immunogenic” in terms of amino acid changes (W, F, Y, V, L, I over others) at MIA positions and if these effects were different due to clonality, histology, or mutational processes. We computed the PRIME %rank score and used it to classify neoepitope as “Immunogenic” or “Non-Immunogenic” (see Methods), on a list comprising 2143 patients (TCGA) from which RNA-seq, HLA haplotyping, clonality, and mutational process origin data were correctly assessed; we also restricted the analysis to only patients with >1 FPKM expression on the genes originating the neopeptide, microsatellite stability, and intact antigen presentation related genes. We analyzed 286,909 neoepitopes from which only 17.75% were predicted to be immunogenic but interestingly they occur more frequently within clonal neoepitope than in subclonal (38% versus 30%, *P* = 1 × 10^−^^27^; two-sided Fisher exact test; Fig. 4a). Because our results suggested a higher presence of immunogenic neoepitopes, in terms of numbers, provoked by mutations occurring earlier, we restricted the subsequent analyses to only immunogenic clonal neoepitopes (ICNs).

Strikingly, albeit a global higher number of APOBEC induced ICNs was present, the proportion of samples having at least one ICN produced by AID (classified as “Present”) was practically three times higher than those provoked by APOBEC globally (32% versus 11%, *P* = 1 × 10^−65^; two-sided Fisher exact test; Fig. 4a). We next sought to compare the cumulative distribution of the AID/APOBEC ICNs in terms of population hotspot mutations recurrency finding that AID produces ICNs at hotspots with greater positive selection (FC = 1.59; *P* = 3e-41, two-sample Z-test for equal proportion, Fig. 4a) which could give rise to higher possibilities of immune recognition and improved tumor control. By comparing tumors harboring at least one AID ICN (“Presence”) to those which did not (“Absence”), we found an increased fraction of CD8, CD4 memory activated and follicular helper T-cells that were “exhausted” by higher expression of the inhibitory immune-checkpoint molecules PD-1, PD-L1, PD-L2, CTLA-4, and LAG3. Furthermore, these observations were seen in the majority of tumor types but the increment was only significant when accounting for all the samples (*n* = 2143) or for LUAD (Fig. 4b, two-sided Wilcoxon test).

Since these findings suggested that AID mutations inducing ICN as a possible explanation of ICI response, we next analyzed a cohort of 68 melanoma patients treated with anti-PD-1 (Nivolumab) from which WES, neoepitopes, and RNA-seq data were available prior treatment (pre) or 4 weeks after initiation of Nivo (on)^34^. Through all the analysis we separated patients as Ipi-Prog (*n* = 35), which had previously progressed on anti-CTLA-4 treatment (Ipilimumab), or as Ipi-Naive, which only received Nivo (*n* = 33). First, we looked at the distribution and effect of AID mutational load on survival compared to UV-related mutations. We found that the responders (Complete-response/Partial-Response) had a higher number of AID-related mutations compared to the stable disease (SD) or progrssive disease (PD) groups (Ipi-Prog median = 0.094; Ipi-Naive median = 0.108), but was not significantly different and was also observed for UV mutations (Ipi-Prog median = 0.354; Ipi-Naive median = 0.349). Conversely, the effect on OS was markedly different, being associated with prognosis only when using AID mutations within Ipi-Naive patients (log-rank *p* = 0.026) but not with UV mutations in neither naive nor progressive patients (log-rank *p* = 0.93 and *p* = 0.34; Supplementary Fig. 15). The AID ICN load improved survival prediction better (log-rank *p* = 0.0016) than if using global clonal neoepitopes load (log-rank *p* = 0.0042), global ICN load (log-rank *p* = 0.0025) or UV ICN load (log-rank *p* = 0.0071; Fig. 4c). As the effect was tightly marked only in Ipi-Naive patients, we focused the subsequent analysis on only this group.

When coupling RNA-seq data (*n* = 20), we found 64 upregulated and 110 downregulated genes comparing patients with high AID ICN load versus low within pre-therapy samples (q < 0.20; Supplementary Table 4). Gene Ontology (GO) analysis identified downregulation of antigen presentation and TNF signaling pathways (q-value <0.05; Fig. 4d and Supplementary Fig. 15). We also observed an increased expression of the inhibitory immune-checkpoint molecules PD-1, PD-L1, PD-L2, CTLA-4, ICOS, LAG3, and cytolytic activity (Supplementary Fig. 16). These results are consistent with both our previous analysis on TCGA data and previous studies^34,37,38^.

Next, we endeavored to identify expression changes on patients that responded (according to AID ICN load) after 4 weeks of Nivo treatment by comparing pre-therapy to on-therapy data from the patients (n_pre_ = 20; n_on_ = 20). From the 811 genes found to be differentially expressed (q < 0.20; Supplementary Table 5), 404 were upregulated and involved in antigen processing and presentation, T-cell activation (e.g., *PRKCQ, CD8B, CD38, CD151, MALT1*), leukocyte cell–cell adhesion, response to oxidative stress (*STK24*, *GSS, GCLC, PDK1*) and T-cell reactivity to clonal neoepitopes (CXCL13 and CCR5) (q-value <0.05), the last ones being recently described^18^. On the other hand, downregulated pathways included mainly cell growth, B-cell differentiation, and some chemokines (*CXCL11, CCL4*, and *CCL14*) or chemokine receptors (*CCR3* and *CCR8*) (Fig. 4d). Furthermore, we also observed an increased expression of *CXCL13* and *CCR5* on the TCGA samples with high AID ICN load (Fig. 4b). Altogether, these results show possible explanations of why AID mutations reflect a more straightforward approach to predict response to Ipi-naive treatment.

### AID synergizes initial hotspot mutations through late mutations on weakly functional alleles

**Fig. 5:**
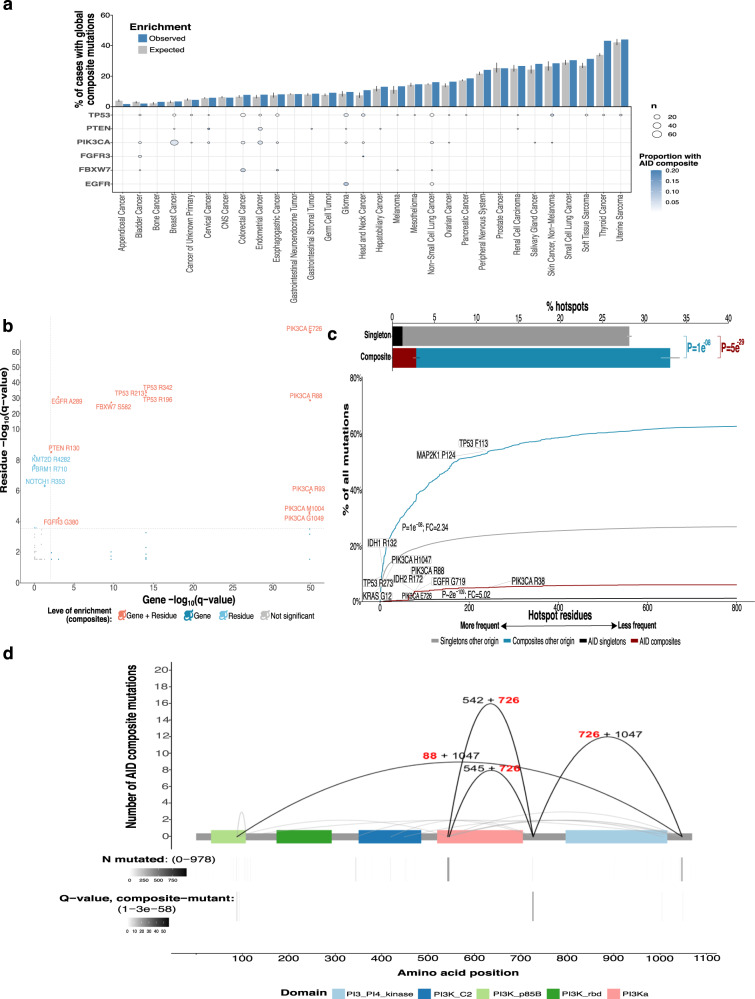
Impact of AID mutations on composite mutations. **a** AID composite mutations in enriched genes by lineage (*n* = 31,353 samples). Cases with global composite mutations and the expected value based on cohort size and mutational burden (top). Significant enrichment for AID composite mutations in cancer genes per cancer type (FDR-adjusted *P*-values from a one-sided binomial test for enrichment; bottom, *n* = 29,461). **b** Residue versus gene enrichment arising from AID composite mutations (FDR-adjusted from one-sided Fisher’s exact test for residues or one-sided binomial test for genes). **c** Cumulative sum of the percentage of hotspot mutation utilization by decreasing frequency of population-level hotspot mutations among composite or single mutations (AID or not AID provoked). Two-sided Mann–Whitney U test, fold-change (FC) of max composite to singleton values. Top inset, percentage of hotspots attributable to composite/singleton mutations (Two-sided two-sample Z-test for equal proportions, color indicates comparison for AID or not AID provoked). **d** Occurrence of PIK3CA AID composite mutations where arcing lines indicate the composite pairs (≥2 tumors, red-bold color for AID enriched residues) and numbers indicate the amino acid position. Residue PIK3CA E726, located between the kinase and PI3KA domains, is highly enriched as an AID composite. Significance values for the composite mutants (FDR-adjusted *P*-value, one-sided binomial test) are shown at the bottom. Error bars indicate 95% binomial confidence intervals.

Since recent studies have unraveled that composite mutations, pair of driver–driver, driver–passenger, or passenger–passenger mutations on the same gene, can synergize the functional impact compared to their single-mutated contra part, we analyzed the contribution of c-AID mutations within this phenomenon by analyzing 31,353 samples comprising 41 tumor types from the MSKCC cohort (Fig. 5). As previously described, using a panel of 353 oncogenes (168 genes) or tumor suppressor genes (TSGs, 185 genes), we found that composite mutations occur more frequently in TSGs than in oncogenes (12.2% versus 6.0% of all mutations; *P* = 2e-278, two-sided two-sample Z-test)^39,40^ but interestingly when separating by c-AID mutations compared to those of other origins, we observed a global contribution to the composite mutations of 6.9%; furthermore, within oncogenes, 9% consisted of at least one c-AID mutation, compared to 5% within TSGs (Supplementary Fig. 17). We further verified that biallelic loss was also enriched for AID composite mutations, as it was reported from global composite mutations, within TSGs since there were more truncating variants compared to oncogenes (64% versus 8%; P ~0; fisher exact test; Supplementary Fig. 17). Next, we calculated gene enrichment for AID composite mutations globally and per tumor type to discard that the observations were due to randomness by modeling the AID composite mutational burden as a function of genetic covariates (see Methods). Surprisingly, we found enrichment for six genes including *FGFR3* especially among HNSCC with 20% corresponding to AID composite mutations, and lower lineage-specific proportions for *EGFR* (8.9% in Glioma), *PIK3CA* (~4% in Breast, Endometrial, Cervical, and Skin cancers), *FBXW7* (~7% in Colorectal and Esophagogastric cancers); *PTEN* (2.5 and 4% in Endometrial and Cervical cancers) but not *TP53* since it was present across different tumor types (Q < 0.01; Fig. 5a and Supplementary Fig. 17, Supplementary Table 6). We used a similar approach for residue’s enrichment to avoid missing residues not enriched at the gene level and observed that PIK3CA E726 was the most enriched (q = 2.59e^−58^, Fisher’s exact test) followed by TP53 R213, EGFR A289, and PIK3CA R88 (Fig. 5b, Supplementary Table 7-8). Since most found residues happened to be of lesser positive selection, we next checked the cumulative proportion moving from frequent hotspots (greatest positive selection) to less frequent ones, finding that AID composite mutations are five times more likely to happen than AID singleton mutations (*P* = 2e-109, two-sample Z-test for equal proportion) which has higher than the fold-change (FC) between composite mutants (other than AID) to singleton mutants (FC = 2.3; P ~ 0). Furthermore, any AID mutation was absent from the highest positive selective hotspots (i.e. KRAS G12, PIK3CA H1047, TP53 R273) suggesting that AID mutations have a preference towards weakly functional alleles after the acquisition of high positive hotspots (Fig. 5c, Supplementary Table 9). To further evaluate this hypothesis, we added the allelic configuration and clonality to subset to mutations arising from the same tumor cell population and retain molecular timing information. We observed that both globally (69% versus 31%, *P* = 7e-4, two-sided binomial test, Supplementary Fig. 17) and within AID composites (73% versus 27%, P = 0.03, two-sided binomial test, Supplementary Fig. 17) the most frequent hotspot mutation occurs first and is followed by a synergizing second mutation but only within oncogenes, which was the case of the minor mutation PIK3CA E726, between the kinase and the PI3KA domains, that occurs significantly after (*p* = 0.039, one-sided binomial test) than other stronger mutations (i.e., PIK3CA E542, PIK3CA E545 at helical domain or PIK3CA H1047 at the kinase domain*)* (Fig. 5d, Supplementary Table 10) and is a product of AID promiscuous activity. When looking only at phase-able mutations (without the molecular timing variable) we observed that 88% of composite mutations on *PIK3CA* occur in cis from which 26% were AID provoked; other genes with a high percentage of cis AID composite mutations were *EGFR, KMT2D*, and *APC* (Supplementary Fig. 17, Supplementary Table 11). Some *PIK3CA* composite mutations have already been proved to increase cell proliferation, tumor growth but also PI3K inhibitor sensitivity in human breast epithelial cell lines, but to the best of our knowledge, it has not been linked to being the product of AID activity^40,41^.

Additionally, we analyzed the contribution of other mutational processes to the composite mutations. Besides the aging signature, AID contributed more to the composite mutations than other signatures (Supplementary Fig. 18), opening the possibility of further research on the molecular implications of these mutations.

## Discussion

By integrating more than 50,000 bulk level samples across 80 tumor types and different data levels, we present, to the best of our knowledge, the first study shedding light on the oncogenic and clinical implications of AID at pan-cancer scale. Our results point to the idea that AID induces traceable mutations with important functional and clinical implications that are mainly produced during the transcriptional activity of the mutated gene. Firstly, by using WGS data, we found that AID mutational load is increased at transcriptionally active TAD domains (compared to the background) and close to TSS. Moreover, regarding the different AID mutational behavior depending on the strand location of the gene, we propose a model where the negative strand is more prone, than the positive strand, to AID attack at naked transcribed breathing dsDNA (normally located near TSS) and is followed by attack at DNA stem-loops and transcription bubbles (but not at R-loops) being generated as the RNA polymerase transcribes^4^.

Summing up the findings that globally AICDA expression and c-AID mutations were not correlated in the TCGA nor in ICGC datasets plus that mostly only the immunoglobulin genes’ expression was correlated with c-AID mutations in hematological cancers, it is tempting to speculate that the genotoxic effect of AID might be due to short-term activation of AICDA, which have been seen in APOBEC^42^. Indeed, in a fate-mapping study, AICDA expression was present in a fraction of non-lymphoid embryonic cells^43^ and also in malignant melanoma cells from a single-cell RNA-seq study comprising 33 melanoma tumors (Supplementary Fig. 19)^44^. Furthermore, AICDA transcripts in lymphocytes have a half-life of only 1 h^45^, supporting the lack of correlation between AID-related mutations and AICDA expression.

Despite, ephemeral, *AICDA* expression mutational footprints are widespread across cancers, and presumptively across mammals, with similar mutational frequency compared to APOBEC but a higher contribution to driver oncogenes, to composite mutations, and to the production of higher quality neoepitopes. Already reported AID off-target activity, outside lymphomas, is limited especially to *TP53, KRAS*, and *MYC* in gastric, colorectal and skin melanoma^16,46,47^. For example, Nonaka et al. demonstrated that mice expressing AID in the skin spontaneously developed skin squamous cell carcinoma with *Hras* and *Trp53* mutations that presented the characteristic AID motif^47^; this was also reported by Sawai et al. in precancerous lesions in AID Tg mice^15^ and by Li et al. in AID-induced mutations within the p53 gene in colorectal cancer^46^. We thoroughly extended this data and found that AID activity has a preference towards least positive selection hotspots that synergizes with previous stronger hotspot mutations; this is the case for the minor mutation PIK3CA E726, especially present in SKCM and BRCA, that might confer higher PI3K inhibitor sensitivity^40,41^.

Finally, we found that the AID-related fraction of mutations is an independent prognostic value to ICI response using >2000 samples even after adjusting by TMB. AID-related neoepitopes exhibited distribution towards clonal hotspots with a greater positive selection which could result in improved immune recognition; however, this is avoided by tumor-induced immune exhaustion. It should be noted that the statistical power in individual histologies is reduced, and as sample sizes increase, additional histology-specific associations may appear in future larger prospective studies that may lead to a formal validation of the predictive value of AID-related signature on ICI response and the results regarding the AID-related neoepitopes. It is also important to highlight that there could be some analytical bias related to the combination of different datasets using different mutation calling approaches. However, the signal associated with the AID-related mutations was similar throughout the studies and the pipelines, and results of the different included studies are public and well standardized, limiting in part this mutation call bias.

We propose a model in which AID ICN has higher probabilities of being recognized by T-cells, triggering selective expression CXCL13, previously found to be a marker of antigen reactive CD8 T-cells, for recruitment of CXCR5 + T and B cells^18^. These recruited cells, subsequently exhausted by the continuous expression of inhibitory immune-checkpoint molecules, can be reinvigorated after ICI treatment.

c-AID mutations are present at pan-cancer level, with higher frequency in B-cell malignancies and other hematological cancers, and higher presence at transcriptionally active TAD domains. AID mutational load predicts response and is associated with favorable outcome in ICI-treated patients, probably due to producing immunogenic clonal neoepitopes at hotspots with greater positive selection that might enhance *CXCL13*/*CCR5* expression, immunogenicity, and T-cell exhaustion. Overall, we pieced together an immense part of the oncogenic AID puzzle but many parts still need to be found, especially filling gaps with biological validations as the results, here presented, hold the promise of important clinical applications.

## Methods

### Subject details

The total cohort at bulk level consisted of 50,631 tumor samples representing more than 80 cancer types. TCGA information consisted of: mutational data in Mutation Annotation Format (MAF) included 9264 cancer patients and 741 normal samples; RNA-seq data; immune data, allele-specific integer copy numbers, and previously predicted neoepitopes. The PCAWG data (ICGC) included 2775 cancer patients along with 35 different cancer types with whole-genome sequencing (WGS) information (SNV and CNV) from which 1522 had the expression data available. Composite mutations data included 31,353 cancer patients from the MSKCC comprising 41 tumor types by the MSK-IMPACT assay (sizes depending on the date of sequencing comprising 341, 410, and 468 cancer-associated targeted genes) downloaded from CBioPortal for the general maf or their github repository (https://github.com/taylor-lab/composite-mutations/tree/master/data) for the clinical, mutational burden classification, mutational signatures, composite mutation annotation, phasing information, and molecular timing^39^. Additionally, hematological cancers cohort (AML, DLBCL, Myelodysplastic Syndromes, and other leukemias; *n* = 3859)^48–61^ and pediatric cancers cohort (20 tumor types; *n* = 1051)^62^ were obtained from CBioPortal some were part of the Therapeutically Applicable Research to Generate Effective Treatments (TARGET) initiative (phs000467, phs000471, and phs000465). ICI cohort consisted of 2261 samples coming from: MSKCC-IMPACT dataset (*n* = 1472), Pender et al. cohort (*n* = 98), Miao et al. cohort (*n* = 249), Liu et al. (*n* = 144, melanoma), Hugo et al. (*n* = 37, melanoma), and Braun et al. (*n* = 261)^19–21,32,33,63^. Riaz et al. melanoma cohort consisted of 68 patients treated with Nivolumab (anti-PD-1) from which 35 had previously progressed on Ipilimumab (anti-CTLA-4) treatment from which data was obtained prior treatment (pre) or 4 weeks after initiation of Nivo (on). Data consisted of whole-exome sequencing (WES), neoepitopes (n_pre_ = 68; n_on_ = 41), and RNA-seq (n_pre_ = 45; n_on_ = 41)^34^. The canine cohort consisted of a total of 187 samples representing 3 tumor types including glioma (*n* = 81), osteosarcoma (*n* = 35), and melanoma (*n* = 1)^64–66^.

### Tracking AID-related mutations

We developed a code to detect c-AID-related mutations over *wrCy/rGyw* (±strand, where “W” stands to either adenine or thymine, “R” to purine, and “Y” to pyrimidine) motifs, giving a total of 8 motifs per strand (positive strand = AACC, AACT, AGCC, AGCT, TACC, TACT, TGCC, TGCT; negative strand = TTGG, TTGA, TCGG, TCGA, ATGG, ATGA, ACGG, ACGA).

The code was developed under R, takes a maf (mutation annotation format) object as input and outputs an S3 class object containing: (i) a matrix of the 768 possible tetranucleotide substitutions across the samples; (ii) a data table with all the needed values for enrichment calculation, the enrichment score, Fisher exact test *p*-value and FDR for enrichment, the fraction of AID mutations, among others; and (iii) a maf-like data table, with the same format as the input, containing only the attributed AID mutations. Finally, mutations were tagged as AID or not AID if overlapping or not with the mutations found in the output maf after applying the function.

### Mutational landscape simulation

We simulated the mutations from each sample from the ICGC cohort to generate a null hypothesis for testing if the c-AID-related mutations happen significantly more by chance or not. Mutations were simulated 1000 times per sample using the tool SigProfilerSimulator^23^ to generate a distribution of mutations in which the mutational processes were reconstructed using a ± 2 bp context around the mutation (SBS-1536 resolution) and the mutational burden was kept the same per sample. c-AID mutations from each of the 1000 simulated lists of mutations were extracted per sample (as explained in the “Tracking AID-related mutations” section); then we evaluated if the observed c-AID mutations were higher than those expected by chance (for each simulation) using a two-sided Fisher exact test. This generated a distribution of odds ratios (OR) per sample (or tumor type) from which significance was considered for OR overlapping 1 in less than 5% of the bootstrap samples.

### AID motifs distribution across the genome

The AID motifs, i.e. WRCY motifs W = adenine or thymine, R = purine, C = cytosine, Y = pyrimidine, that is AACC, AGCC, AACT, AGCT, TACC, TGCC, TACT, and TGCT. We used an R script to find these patterns in the GRCh38 genome using the Biostrings package v2.60.0. In addition, we also calculated the number of these AID motifs in 20 kb binned windows throughout the genome using bedtools, adjusting by the chromosome size.

### c-AID mutations genomic distribution

The inter-mutational distance, i.e., the distance between each somatic substitution and the substitution immediately prior, was calculated per tumor type by combining the MAF data of all samples within that tumor type using the R package “karyoploteR”^67^. Mutation density was calculated within 1 megabase window and added to rainfall plots in Supplementary Figs. 4–8. Furthermore, within each tumor type, mutation density per chromosome was calculated for each sample to then compare densities within chromosomes.

### Attributing mutations to mutagenic processes

Mutations previously tagged as not AID were subjected to signature attribution to 46 (we excluded signatures SBS: 27, 39, 43, 45–60 since they are attributed to sequencing artifacts) of the 78 COSMIC mutational signatures (v3.2) using *Palimpsest* package with default parameters^6,8,68^. To avoid over-fitting, signatures not contributing with at least one mutation within 50% of the samples per tumor type (Median-SBS_n_^TumorX^ < 1) were removed and mutations were re-fitted using the remaining signatures. Furthermore, signatures proportions per sample were re-calculated adding the number of previously identified AID mutations to the signature data. Cosine similarity scores of comparing our tetranucleotide-based c-AID mutations to the 78 COSMIC signatures, were computed using the ‘compare_results’ and ‘deconvolution_compare’ functions from the *Palimpsest* package. Signatures were not calculated from the MSKCC-Composites and ICI cohorts because they were already available or not used.

### Clonality analysis

Clonal and subclonal categorization of mutations was done only on the TCGA and PCAWG (only somatic chromosomes) cohorts from which allele-specific integer copy numbers, ploidy, and purity estimates were available (*n* = 7216 and *n* = 2707, respectively). As previously described, for each mutation the cancer cell fraction (CCF) along with the 95% CI (binomial distribution) was determined through the variant allele fraction, tumor purity, and local copy numbers, then we assigned a mutation (using *Palimpsest* package) as subclonal if the upper boundary of the 95% CI CCF value was inferior to 0.95, or clonal otherwise^68^.

### Attributing mutations as driver genes

Positively-selected genes per tumor type within each cohort, excluding MSKCC-Composites from which we used the already curated list from the original article^39^, were obtained by calculating dN/dS likelihood ratios (*dNdScv* package) through negative binomial regression modeling of the background mutation rate of each gene using distinct genomic covariates including variation in mutation density across genes, context-dependent substitutions (mutational signatures), transcriptional strand bias, chromatin state, expression and replication time. Additionally, we removed ultra-hypermutator samples and extremely mutated genes per sample, to avoid loss of sensitivity. Genes were considered as drivers if having q-values < 0.01 (Benjamini–Hochberg’s multiple testing correction of p-values)^69^. In addition, in the ICGC cohort, we also used the selection intensity of every particular mutation related to APOBEC or c-AID mutations by deconvolution of prevalence by mutation rates for recurrent amino acid mutations within three oncoproteins caused by single-nucleotide changes using cancereffectsizeR v2.1.3 package. The observed substitution rates were divided by the expected substitution rates in the absence of selection. The expected substitution rates in the absence of selection were calculated as the average per-site synonymous mutation rate of the gene, normalized for the average weight of trinucleotide mutational signature burden for that signature. The quotient of observed to expected numbers of substitutions was the selection intensity, as previously described^25^.

### Mutation distribution around TAD boundaries

To compare the mutational load distribution generated by different mutational signatures, we calculated the ratio of mutational burden in transcriptionally inactive domains versus transcriptionally active domains. First, for each sample, we binned the mutations in 25 kb nonoverlapping windows along the genome. Next, we calculated the sum of mutations at inactive and active domains and normalized this value by the length of active and inactive domains. We calculated the total number of mutations in each domain and divided by the domain length (heterochromatin: 180; inactive: 1219; repressed: 969; active: 1086; active-2: 593) for box plot representations (Fig. 2b right), as previously described^28^.

### Mutations distribution within R-loops and transcription correlation

Genomic regions coordinates file with GC skew enrichment, which is associated with R-loop formation, downloaded from a previous study as GRCh38 assembly, was transformed to GRCh19 assembly using USCS genome browser liftover function^26,70^. This gave rise to 16,223 regions (median 626 bp) from which only G skewed regions (8059; associated with R-loops formation during transcription) were used to find overlaps with the genomic position of each mutation and to label them as “in R-loops” or “out R-loops”.

Transcription correlations with c-AID-related mutations were performed by the Spearman correlation method. For each tumor type, we correlated the number of c-AID-related mutations occurring in gene i (AID_Muts_gi_) to the expression of the same gene i (Exp_gi_). The AID_Muts_gi_ were considered associated with the transcription process in a specific tumor type if the adjusted p-value was inferior to 0.05 and the Spearman Rho value superior to 0. Functional enrichment analysis of the associated genes was performed using the DAVID database^71^.

### Replication timing

Replication timing measurements of 11 different cell lines (SK-N-SH = neuroblastoma; MCF-7 = mammary gland adenocarcinoma; BJ = skin fibroblast; NHEK = epidermal keratinocytes; HepG2 = liver carcinoma; IMR90 = fetal lung fibroblasts; K562 = leukemia; HeLa-S3 = cervical carcinoma; GM12878 = lymphoblastoid; HUVEC = umbilical vein endothelial cells; BG02ES = embryonic stem cell) were downloaded as wavelet-smoothed signal and then used to compute the median Repliseq signal within 1 Mb or 25 Kb (for TADS) windows which resulted in values from 0–100 indicating late to earlier replication times. We further proceeded to divide the genome into 1 Mb regions (or 25 Kb) by first masking out all regions in the genome that requires 36-mer to be unique in the genome even after allowing for two differing nucleotides and also the Duke and DAC blacklisted regions (highly possible anomalous signals) using BEDTools^72^, this led to 3,053 1 Mb windows. Then we calculated the mean number of mutations attributed to c-AID or APOBEC (COSMIC SBS2 and SBS13), per tumor type, as the mutation number within each bin divided by the number of samples within the corresponding tumor type, then we coupled the corresponding repliseq data (as decile distributed) for each bin.

### Neoepitope analysis

TCGA neoepitope list and HLA haplotypes were retrieved from previous studies, briefly 4-digit HLA type for each sample was inferred using POLYSOLVER, and neoepitopes were predicted using NetMHCpan (v2.4) (only for MHC class I) as novel 9–10mers that resulted from mutations in expressed genes (>10 TPM) and affinity <500 nM^73,74^. We coupled this information with the data resulting from our analysis to obtain neoepitopes data (*n* = 3370 patients) with clonality, signature origin, expression, PolyPhen/SIFT effect on coding protein, among others. We furthered filtered for >1 FPKM expression on the genes originating the neopeptide and excluded patients with: I) incomplete HLA information; II) microsatellite instability (retrieved from Ding et al.)^75^; III) altered antigen presentation related genes *HLA-A, HLA-B, HLA-C, CIITA, IRF1, PSME1, PSME2, PSME3, ERAP1, ERAP2, HSPA, HSPC, TAP1, TAP2, TAPBP, CALR, CNX, PDIA3,* and *B2M* (HLA enhanceosome, peptide generation, chaperones, and the MHC complex) which was based if presence of PolyPhen/SIFT damaging predictions (“probably_damaging”/“possibly_damaging” or “deleterious”/“deleterious_low_confidence”, respectively) or of copy number loss. These filters left 2143 patients that were considered for Fig. 4a and b and Supplementary Fig. 15. To account for immunogenicity based on the prediction of neopeptide TCR recognition and neopeptide HLA binding, PRIME software was run with default parameters. Neoepitopes were classified as “Immunogenic” if having a PRIME %rank score (the fraction of random 700,000 8- to 14-mers that would have a score higher than the peptide provided in input) lower or equal to 0.5% for the corresponding HLA haplotype of the patient where the neopeptide occurred, or as “Non-Immunogenic” otherwise^35^. For simplicity some cosmic signatures were grouped as: MMR (SBS6, SBS15, SBS20, SBS21, SBS26, and SBS44); Smoking-associated (SBS4, SBS18, SBS24, and SBS29); POLE (SBS10a, SBS10b, and SBS14), and APOBEC (SBS2 and SBS13). This data combined with the clonality of the mutation giving rise to a specific neopeptide was used to classify them as immunogenic clonal neoepitope (ICN); samples were further classified as “Presence” if having at least one ICN due to a specific mutational signature or “Absence” otherwise. TCGA RNA counts were retrieved from GEO GSE62944 and normalized using the variance-stabilizing transformation (VST) function from DESeq2^76^.

For the advanced melanoma anti-PD-1 treated cohort^34^ mutational signatures, clonality, immunogenicity, and RNA counts normalization was assessed as described above. Samples were classified as having high ICN AID load if their load was superior to the cohort’s median. Furthermore, differential gene expression analysis (Ipi-Naive samples only) was executed with DESeq2 comparing high ICN AID load versus low within pre-therapy samples only (Supplementary Fig. 16) or comparing pre-therapy to on-therapy adjusting for AID ICN load groups (high or low) to identify expression changes on-therapy (design = ~CIN_AID_binary+PreOn). For each comparison, DEGs (adjusted *p*-value <0.2) were used as input for hierarchical clustering (Euclidean distance followed by complete-linkage agglomeration algorithm) to obtain gene clusters used for enrichment analysis (Fig. 4d). GSEA analysis was performed using the GO database through R package clusterProfiler^77^ or DAVID database^71^ applying Bonferroni correction (q-value <0.05).

### Gene and residue-specific composite mutation enrichment testing

The main code for analysis was obtained from the original article and then subjected to minor modifications for identification of c-AID-related mutation’s contribution^39^. In brief, the number of samples harboring a composite mutation in permutation i (n_i_) was obtained by permuting 100,00 times the z component of a matrix of m x z (m = total number of nonsynonymous somatic mutations; z = mutation id as a mutation in ‘*x’* gene occurring in an ‘*x’* sample). A *p*-value was calculated as the fraction of permutations satisfying n_i_ ≥ n^pos^.

Enrichment of AID composites per gene was assessed using a binomial test to evaluate, for each gene, that the proportion of observed AID composite mutated samples differs from the proportion of predicted AID composite mutated samples arising by chance (n_c_). Negative binomial regression was used for estimating n_c_ per gene by modeling the observed number of AID composite-mutant samples adjusted for multiple genomic covariates like coding sequence length (l), GC content percentage (g, Biomart GRCh37), replication time (r), chromatin state (h)^78^, MSK-IMPACT targeted assay version (i) and the average total DNA copy number of the gene across its mutated samples (t). Additionally, an offset term was added to the model that represents the log number of tumor samples harboring mutations in the gene of interest. Enrichment at individual mutant residues arising as AID composite mutations were assessed using a right-sided Fisher’s exact test comparing if it arose significantly more frequently than all other mutant residues within the same gene. Residues and genes were considered significant if having an FDR corrected *p*-value less than 0.01.

### Analysis of single-cell RNA sequencing data

We downloaded the expression matrix of the raw count, transcript per million (TPM), or Fragments Per Kilobase of transcript per Million mapped reads (FPKM) from Jerby-Arnon et al. (2018)^44^. We collected sample information such as the patient ID, tissue origin, treatment condition, and response groups. For processing all the collected datasets, including quality control, batch effect removal, cell clustering, differential expression analysis, cell-type annotation, malignant cell classification, and gene set enrichment analysis that internally uses the Seurat package^79^. The raw count, TPM or FPKM table was used as input for the standardized workflow. The quality of cells was determined by two metrics: the number of total counts (UMI) per cell (library size) and the number of detected genes per cell. Low-quality cells were filtered out if the library size was <1000, or the number of detected genes was <300.

### Statistical analyses and figures

All statistical analyses were performed using the R statistical programming environment (version 4.0). Figures were generated using either base R or the ggplot2 library. Differences in proportions were calculated from Fisher’s exact test or two-sample Z-tests. Error bars indicate the 95% binomial CIs calculated using the Pearson-Klopper method. Kruskal–Wallis test was used to test for a difference in distribution between three or more independent groups, and Mann–Whitney U test was used for differences in distributions between two population groups unless otherwise noted. Spearman correlations were calculated by the cor.test function in R. *P*-values were corrected for multiple comparisons using the Benjamini–Hochberg method when applicable. For heatmap representation (ComplexHeatmap R package)^80^, VST gene expression values were first quantile normalized and log2 transformed and then converted to Z-scores. Overall survival analysis to ICI was assessed using log-rank Kaplan–Meier curves and univariate/multivariate Cox proportional hazards regression modeling. We have assessed several Cox proportional models for every study (i.e., analyzing the deciles, from 10th to 90th, of the fraction of c-AID mutations in every included study, unadjusted, using the median of the fraction of AID mutations, and also these models were adjusted by TMB ≥ 10mut/Mb). To combine the different survival models, we used a random-effects model with the meta v4.18-1 package^81^, using log hazard ratio and standard errors of each model per study. The inverse variance method was used for pooling. The random-effects estimate was based on the DerSimonian-Laird method^82^. The meta-analysis results were represented in a forestplot using the *forestplot* function of the ggforestplot v0.1.0 package.

### Reporting summary

Further information on research design is available in the Nature Research Reporting Summary linked to this article.

## Supplementary information

Supplemental material

REPORTING SUMMARY

## Data Availability

Our findings are supported by data that are available from public online repositories. The sources of each of the datasets, as well as Accession Codes or other unique identifiers, are available in the Supplementary Information within the Key Resources table (online version of this article).

## Code availability

Software versions used in this study are specified in the Supplementary Information within the Key Resources table. Each Software was run with default parameters, unless specified otherwise within the methods section. The code for the main analyses/figures generated during this study, as well as the R-based code to detect the c-AID-related mutations are available at https://github.com/iS4i4S/Landscape-AICDA-mutations.
