# Supplementary material for: Pan-cancer landscape of AID-related mutations, composite mutations, and their potential role in the ICI response
Source: NPJ Precis Oncol. 2022 Dec 1;6:89. doi: 10.1038/s41698-022-00331-2 (PMC9715662; doi:10.1038/s41698-022-00331-2)
Supplement: Supplementary file 1 — Supplemental material [file 41698_2022_331_MOESM1_ESM.pdf]

Supplementary Information

Supplementary Figures

a

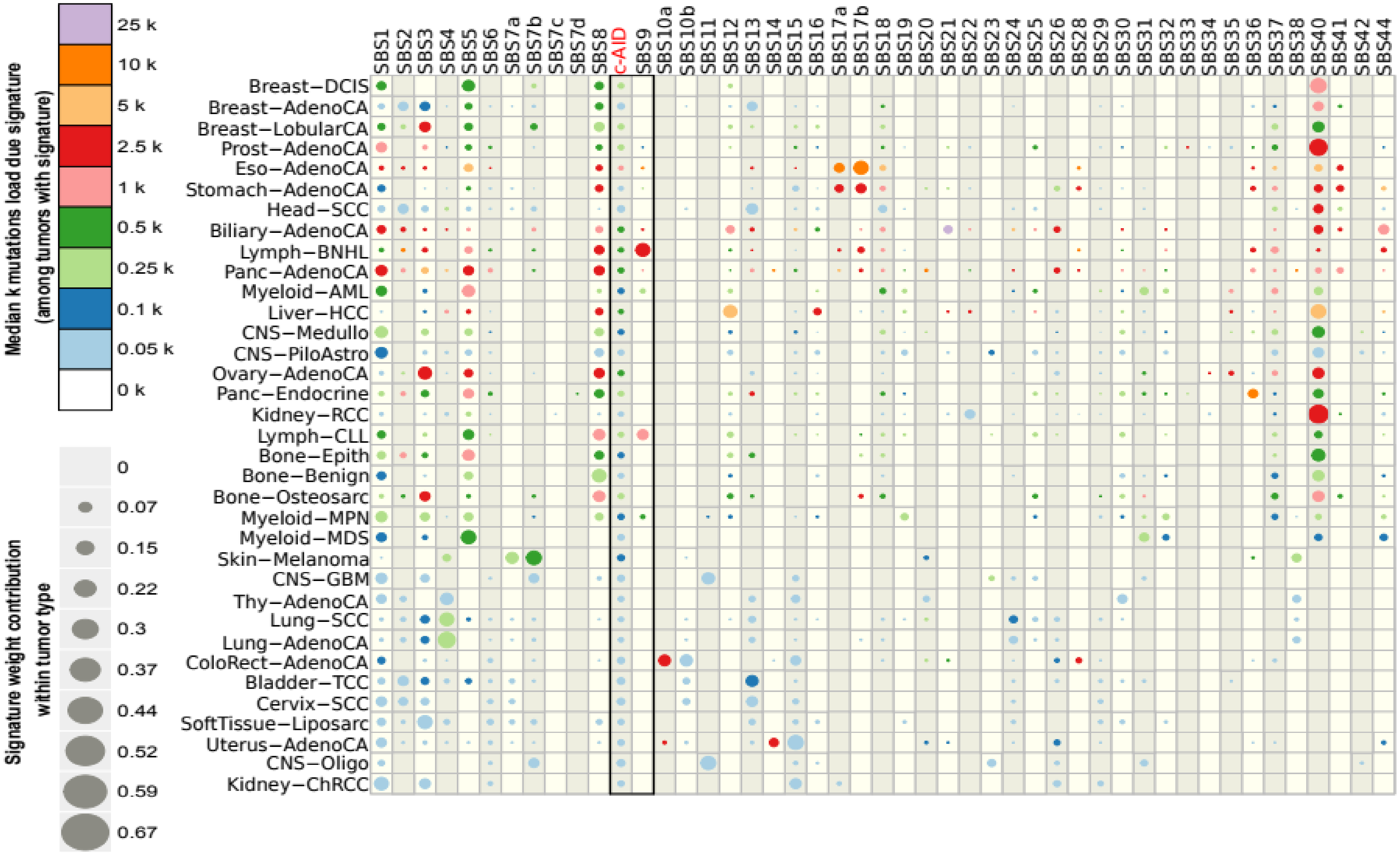
Median k mutations load due signature (among tumors with signature)
25 k
10 k
5 k
2.5 k
1 k
0.5 k
0.25 k
0.1 k
0.05 k
0 k
Signature weight contribution within tumor type
0
0.07
0.15
0.22
0.3
0.37
0.44
0.52
0.59
0.67
SBS1
SBS2
SBS3
SBS4
SBS5
SBS6
SBS7a
SBS7b
SBS7c
SBS7d
SBS8
c-AID
SBS9
SBS10a
SBS10b
SBS11
SBS12
SBS13
SBS14
SBS15
SBS16
SBS17a
SBS17b
SBS18
SBS19
SBS20
SBS21
SBS22
SBS23
SBS24
SBS25
SBS26
SBS28
SBS29
SBS30
SBS31
SBS32
SBS33
SBS34
SBS35
SBS36
SBS37
SBS38
SBS40
SBS41
SBS42
SBS44
Breast−DCIS
Breast−AdenoCA
Breast−LobularCA
Prost−AdenoCA
Eso−AdenoCA
Stomach−AdenoCA
Head−SCC
Biliary−AdenoCA
Lymph−BNHL
Panc−AdenoCA
Myeloid−AML
Liver−HCC
CNS−Medullo
CNS−PiloAstro
Ovary−AdenoCA
Panc−Endocrine
Kidney−RCC
Lymph−CLL
Bone−Epith
Bone−Benign
Bone−Osteosarc
Myeloid−MPN
Myeloid−MDS
Skin−Melanoma
CNS−GBM
Thy−AdenoCA
Lung−SCC
Lung−AdenoCA
ColoRect−AdenoCA
Bladder−TCC
Cervix−SCC
SoftTissue−Liposarc
Uterus−AdenoCA
CNS−Oligo
Kidney−ChRCC

b

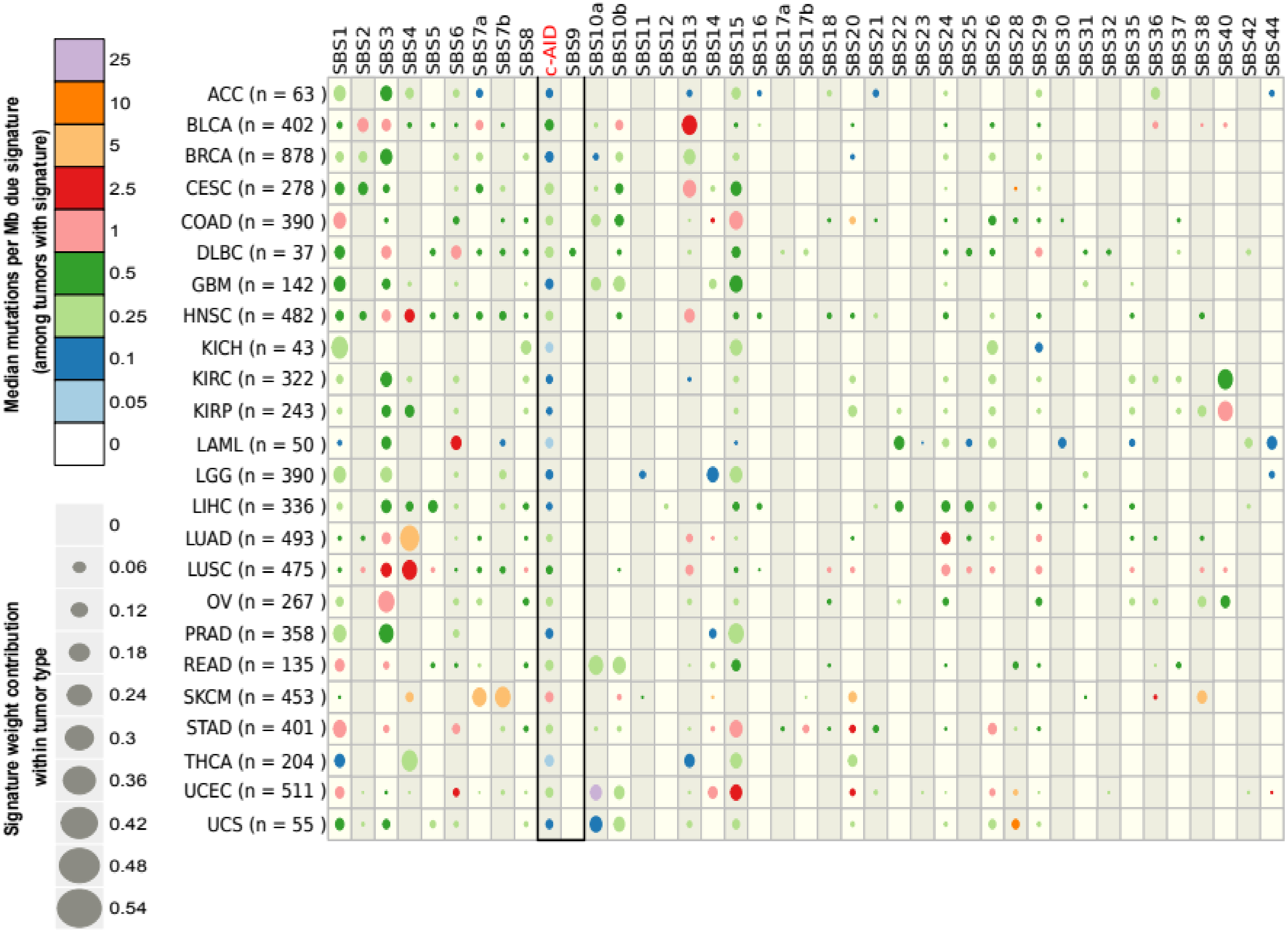

Median mutations per Mb due signature
(among tumors with signature)
25
10
5
2.5
1
0.5
0.25
0.1
0.05
0
Signature weight contribution
within tumor type
0
0.06
0.12
0.18
0.24
0.3
0.36
0.42
0.48
0.54
SBS1 SBS2 SBS3 SBS4 SBS5 SBS6 SBS7a SBS7b SBS8 c-AID SBS9 SBS10a SBS10b SBS11 SBS12 SBS13 SBS14 SBS15 SBS16 SBS17a SBS17b SBS18 SBS20 SBS21 SBS22 SBS23 SBS24 SBS25 SBS26 SBS28 SBS29 SBS30 SBS31 SBS32 SBS35 SBS36 SBS37 SBS38 SBS40 SBS42 SBS44
ACC (n = 63)
BLCA (n = 402)
BRCA (n = 878)
CESC (n = 278)
COAD (n = 390)
DLBC (n = 37)
GBM (n = 142)
HNSC (n = 482)
KICH (n = 43)
KIRC (n = 322)
KIRP (n = 243)
LAML (n = 50)
LGG (n = 390)
LIHC (n = 336)
LUAD (n = 493)
LUSC (n = 475)
OV (n = 267)
PRAD (n = 358)
READ (n = 135)
SKCM (n = 453)
STAD (n = 401)
THCA (n = 204)
UCEC (n = 511)
UCS (n = 55)

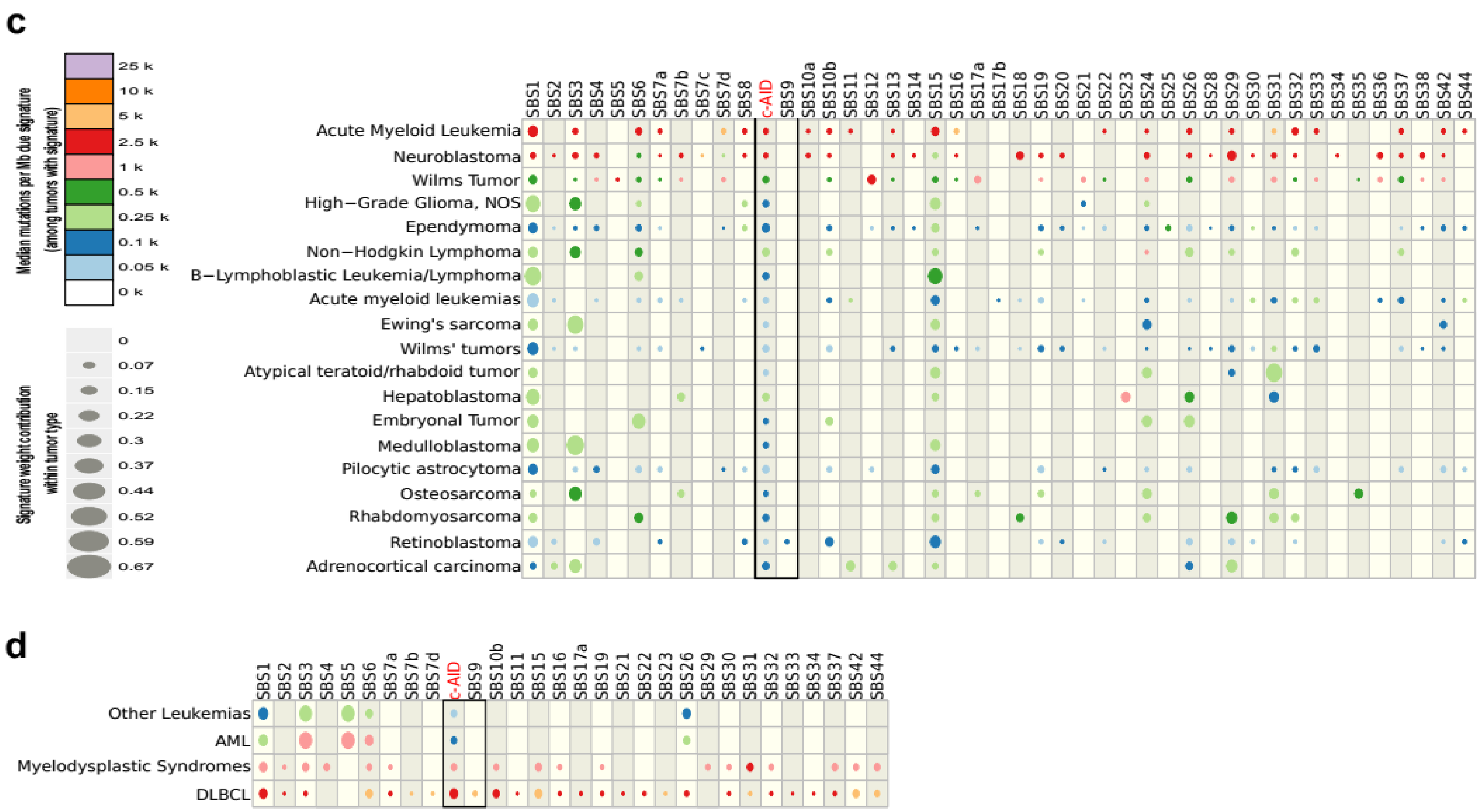

Supplementary Figure 1. AID-related mutations distribution across cohorts

Summary of the frequency of the different COSMIC SBS signatures, including the signature related with AID within the ICGC (**a**), TCGA (**b**), Pediatric (**c**), and hematological (**d**).

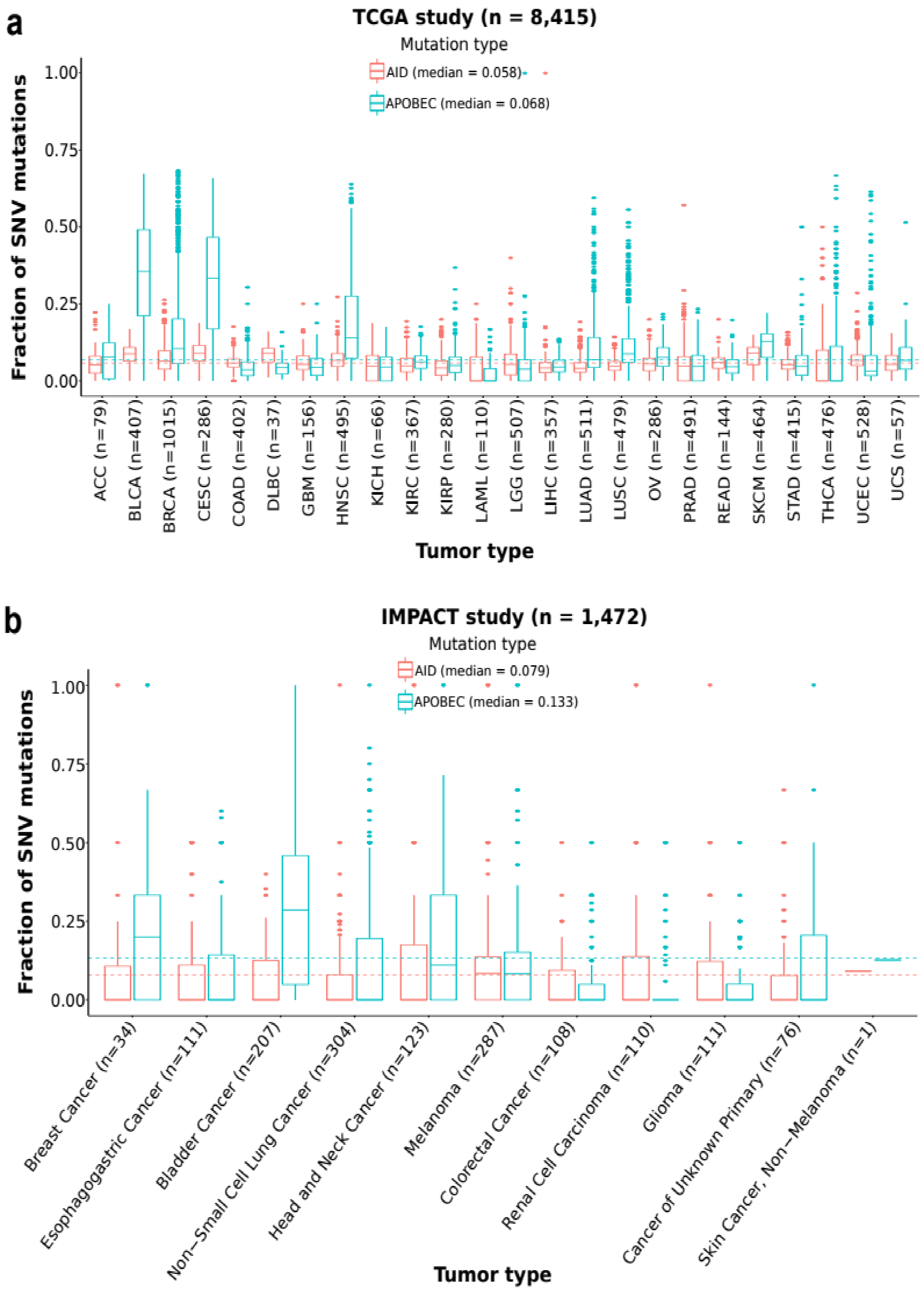
a
TCGA study (n = 8,415)
Mutation type
AID (median = 0.058)
APOBEC (median = 0.068)
Fraction of SNV mutations
1.00
0.75
0.50
0.25
0.00
ACC (n=79)
BLCA (n=407)
BRCA (n=1015)
CESC (n=286)
COAD (n=402)
DLBC (n=37)
GBM (n=156)
HNSC (n=495)
KICH (n=66)
KIRC (n=367)
KIRP (n=280)
LAML (n=110)
LGG (n=507)
LIHC (n=357)
LUAD (n=511)
LUSC (n=479)
OV (n=286)
PRAD (n=491)
READ (n=144)
SKCM (n=464)
STAD (n=415)
THCA (n=476)
UCEC (n=528)
UCS (n=57)
Tumor type
b
IMPACT study (n = 1,472)
Mutation type
AID (median = 0.079)
APOBEC (median = 0.133)
Fraction of SNV mutations
1.00
0.75
0.50
0.25
0.00
Breast Cancer (n=34)
Esophagogastric Cancer (n=111)
Bladder Cancer (n=207)
Non−Small Cell Lung Cancer (n=304)
Head and Neck Cancer (n=123)
Melanoma (n=287)
Colorectal Cancer (n=108)
Renal Cell Carcinoma (n=110)
Glioma (n=111)
Cancer of Unknown Primary (n=76)
Skin Cancer, Non−Melanoma (n=1)
Tumor type

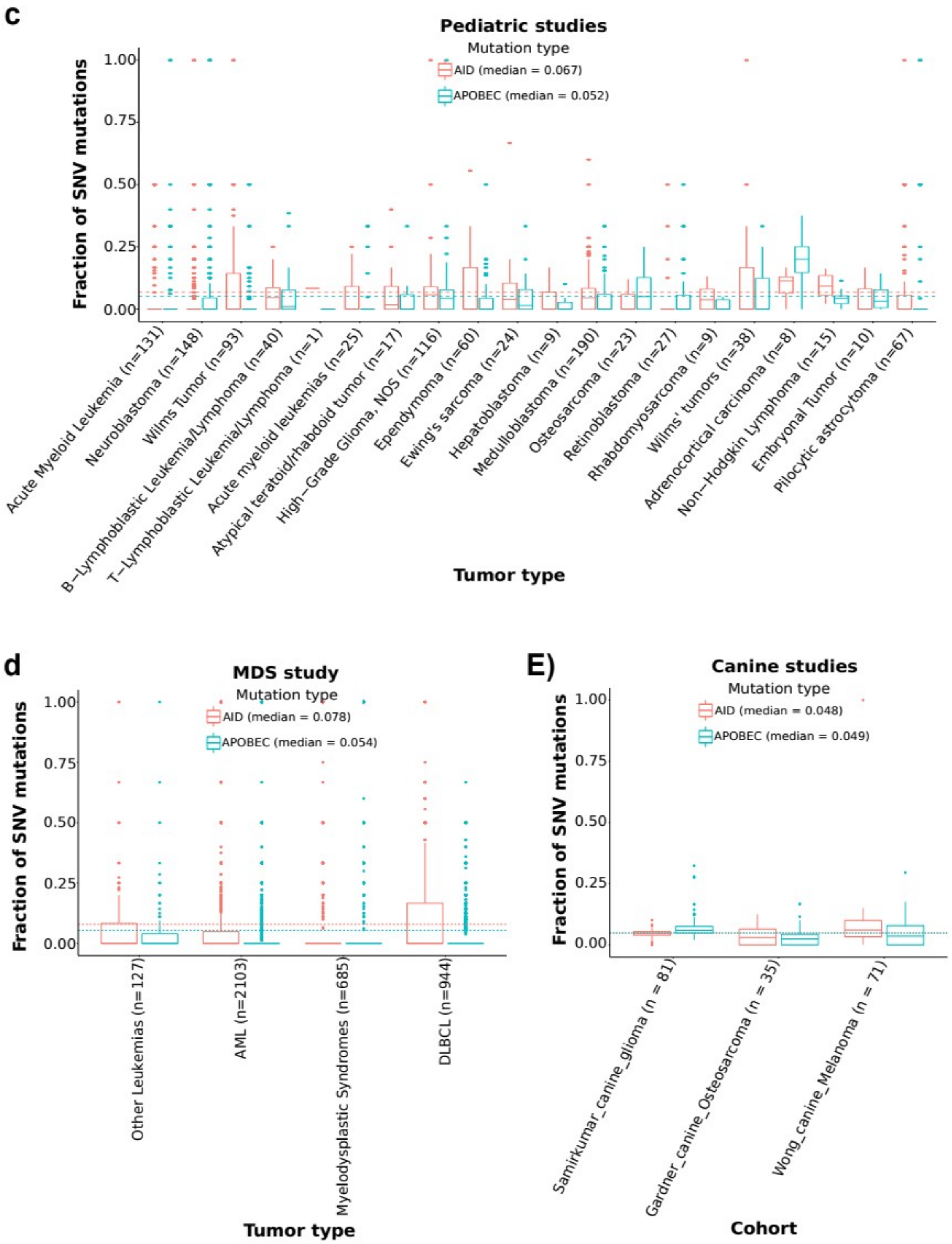

Supplementary Figure 2. Mutational signatures distribution and AID/APOBEC mutations frequency across cohorts

Frequency of AID and APOBEC-related mutations in the TCGA (**a**), MSKCC (**b**), Pediatric (**c**), Hematological (**d**), and canine (**e**) cohorts. Centre line represents the median values; error bars represent the upper and lower quartiles and whiskers define the minimal and maximum values.

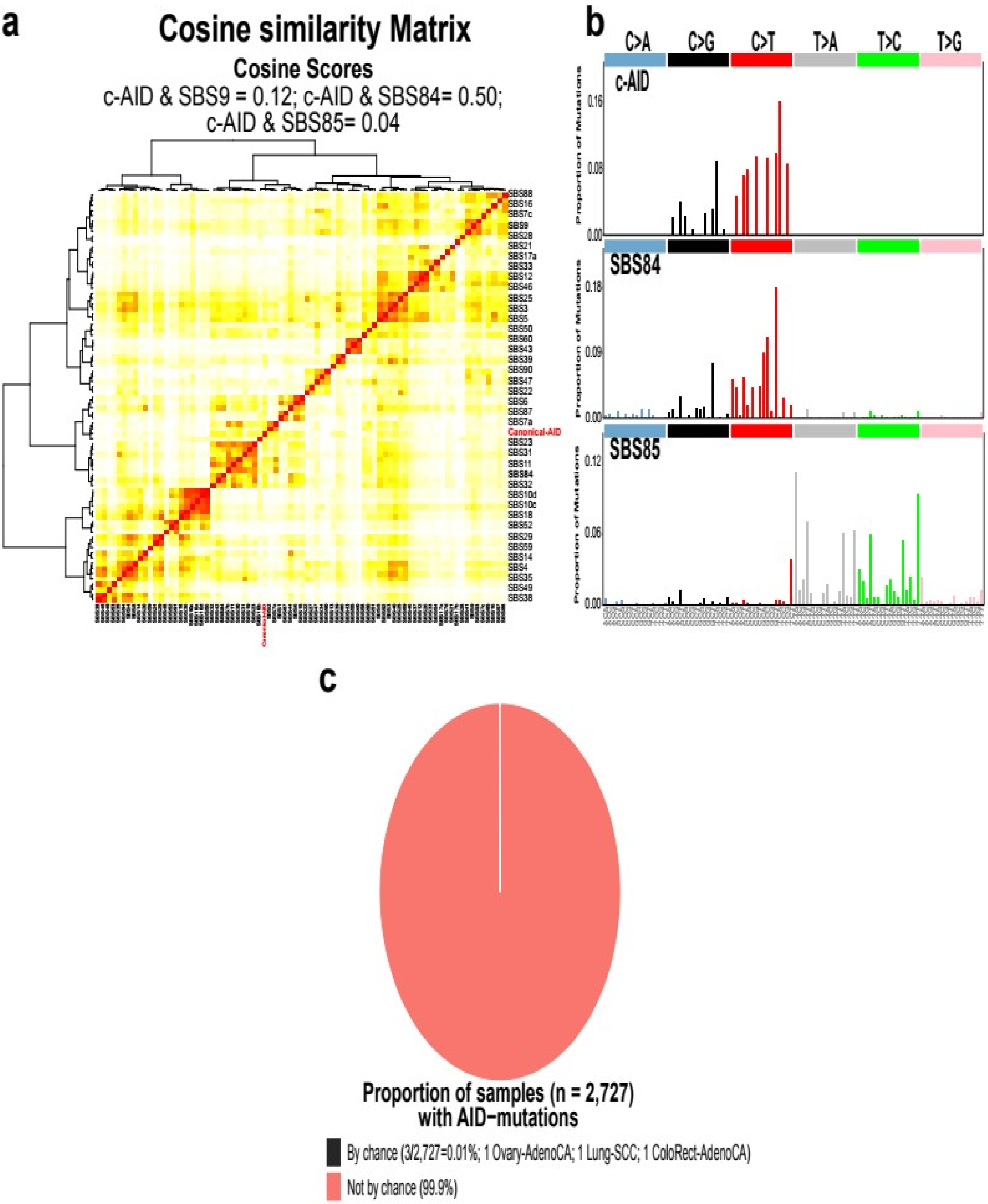

Supplementary Figure 3. Evaluation of tetranucleotide-based c-AID mutations with tri-nucleotide based (COSMIC signatures)

Cosine similarity matrix between our tetranucleotide-based c-AID signature and the cosmic SBS9, SBS84 and SBS85 signatures (**a**). 96-trinucleotide combinations resulting from each signature (**b**). Proportion of samples having significantly more c-AID mutations by chance (two-sided Fisher exact test) by simulating each sample's mutations 1,000 times, while maintaining the mutational patterns at pentanucleotide resolution (SBS-1536, see Methods).

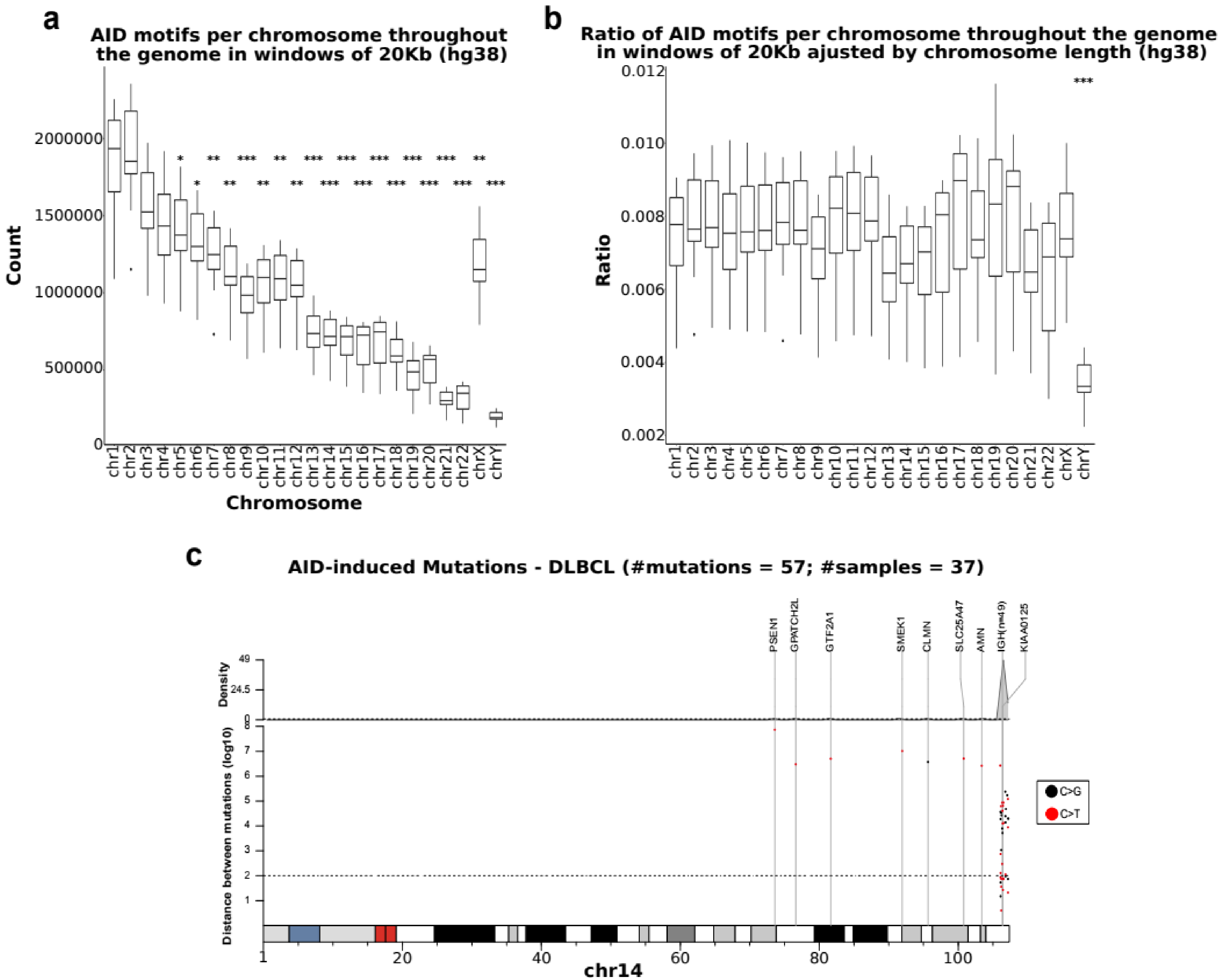
a
AID motifs per chromosome throughout the genome in windows of 20Kb (hg38)
Count
Chromosome
b
Ratio of AID motifs per chromosome throughout the genome in windows of 20Kb ajusted by chromosome length (hg38)
Ratio
c
AID-induced Mutations - DLBCL (#mutations = 57; #samples = 37)
Density
Distance between mutations (log10)
PSEN1
GPATCH2L
GTF2A1
SMEK1
CLMN
SLC25A47
AMN
IGH(n=49)
KIAA0125
C>G
C>T
chr14

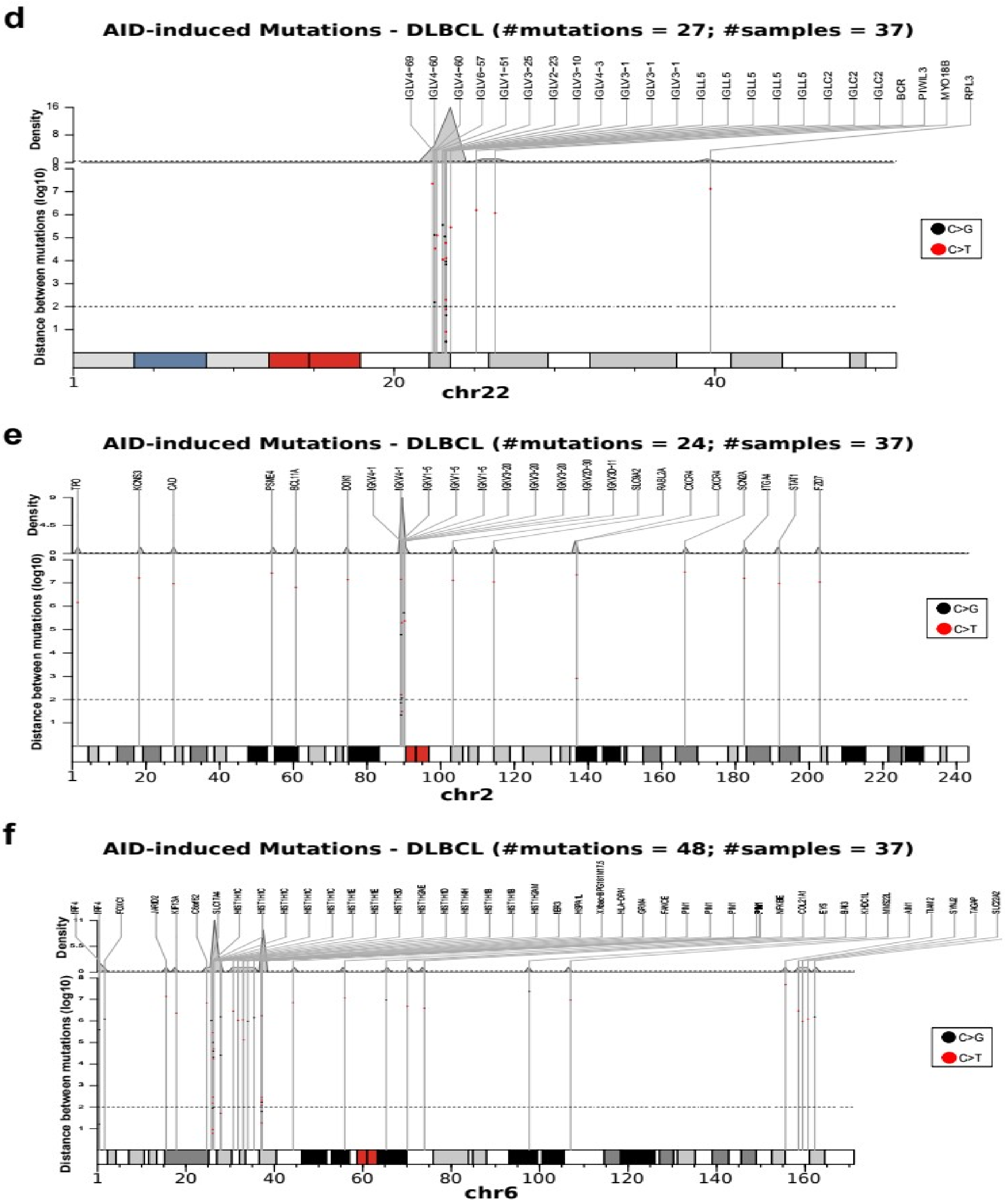

Supplementary Figure 4. AID motifs/mutations genomic distribution in normal genome or DLBCL tumors

Genomic distribution of AID related motifs across chromosomes as 20 kb window raw counts (**a**) or counts adjusted by chromosome length (**b**). Chromosome 2 was used as the reference group for Wilcoxon-test (FDR adjusted p-values). Centre line represents the median values; error bars represent the upper and lower quartiles and whiskers define the minimal and maximum values. **c-f** Rainfall plots of the AID mutations' distribution in DLBCL tumors (TCGA cohort) on chromosomes 14, 22, 2, and 6, respectively.

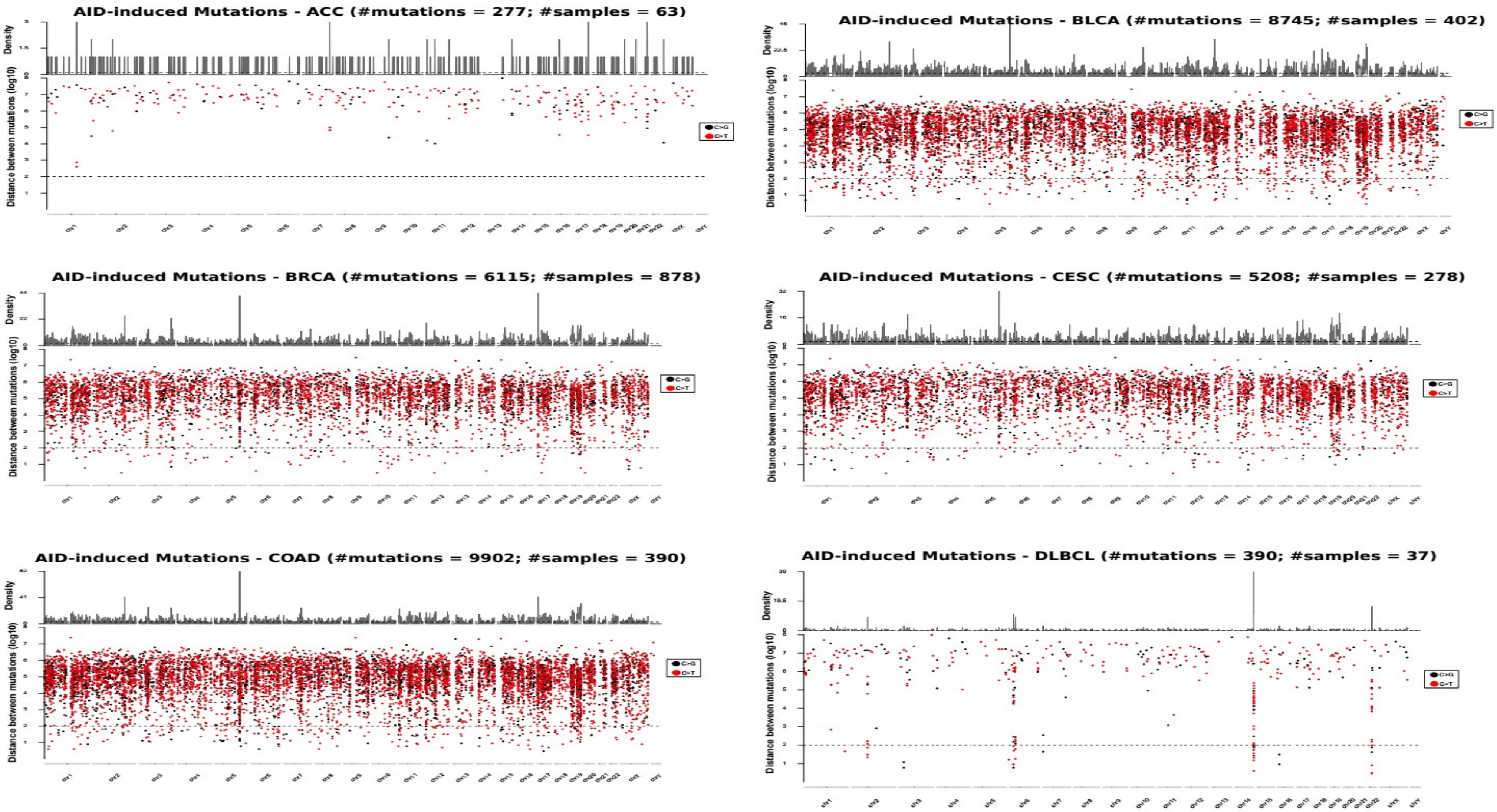

Supplementary Figure 5. AID mutations genomic distribution TCGA (Tumors ACC, BLCA, BRCA, CESC, COAD, and DLBCL)

Rainfall plots of the AID mutations' distribution across chromosomes on TCGA samples by tumor types as a function of logarithmic (10 scale) genomic distance. Black points represent C to G mutations and red points C to T mutations. Top barplot shows the sum of mutations (density) across chromosomes.

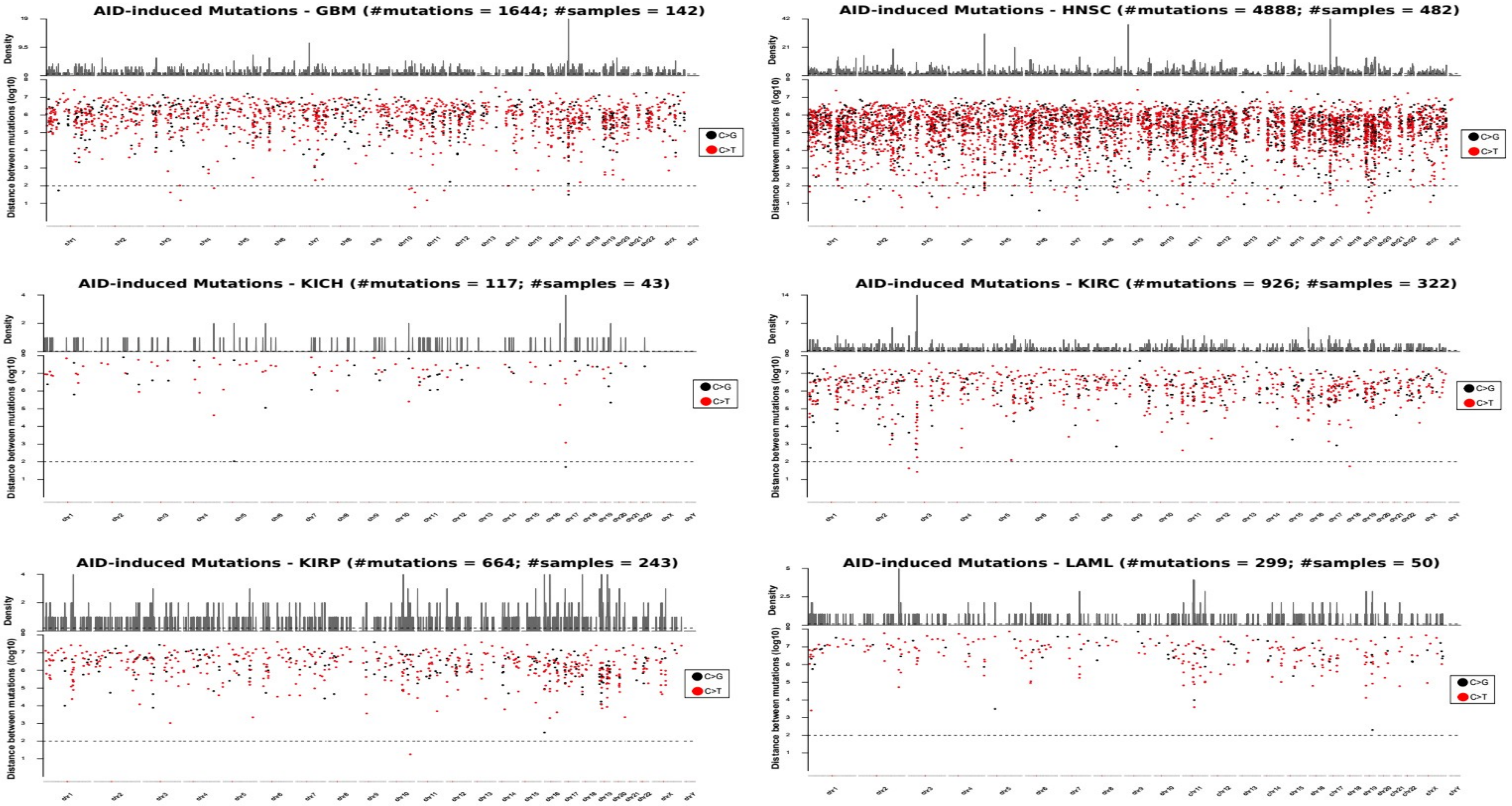

Supplementary Figure 6. AID mutations genomic distribution TCGA (Tumors GBM, HNSC, KICH, KIRC, KIRP, and LAML)
Rainfall plots of the AID mutations' distribution across chromosomes on TCGA samples by tumor types as a function of logarithmic (10 scale) genomic distance. Black points represent C to G mutations and red points C to T mutations. Top barplot shows the sum of mutations (density) across chromosomes.

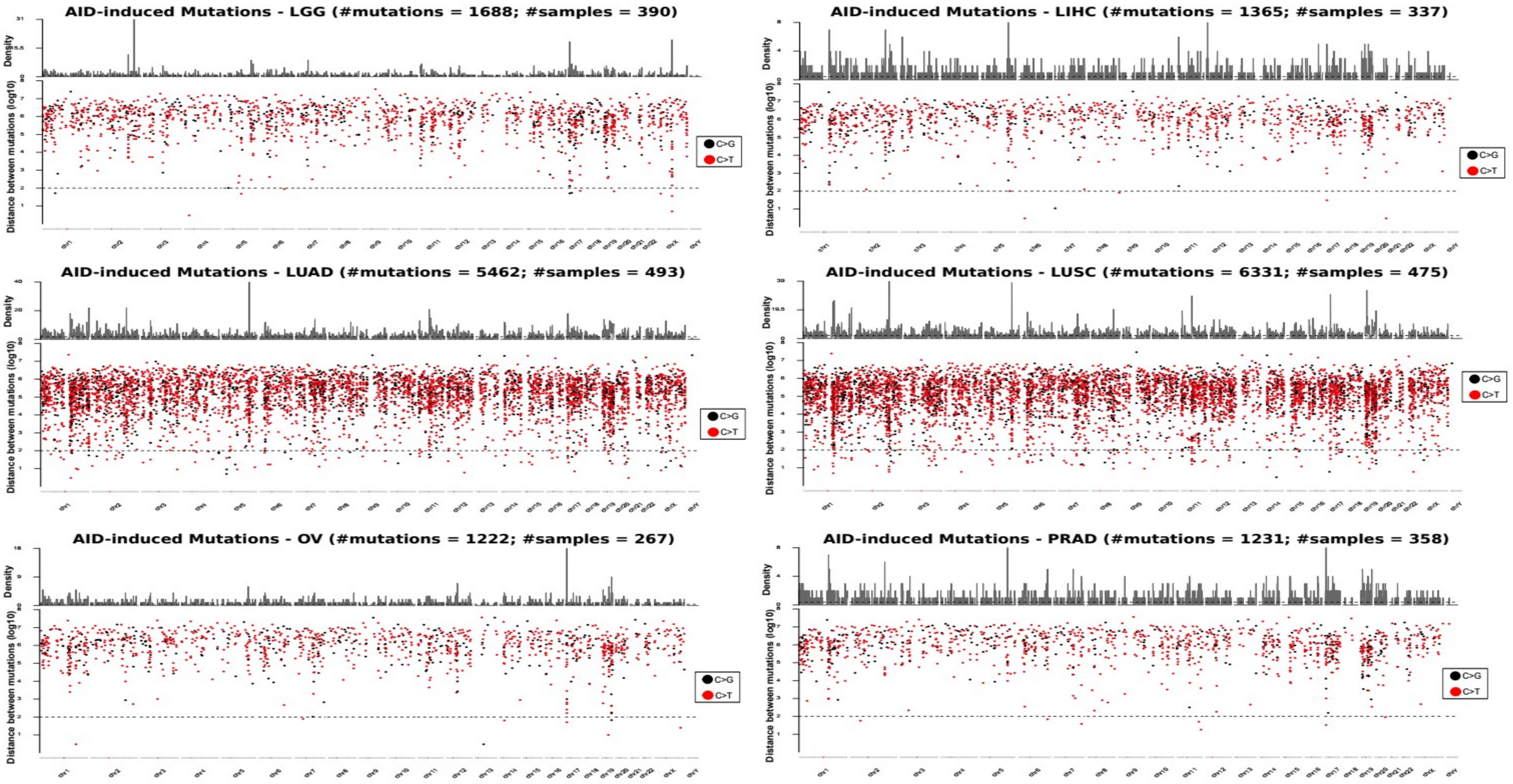

Supplementary Figure 7. AID mutations genomic distribution TCGA (Tumors LGG, LIHC, LUAD, LUSC, OV, and PRAD)
Rainfall plots of the AID mutations' distribution across chromosomes on TCGA samples by tumor types as a function of logarithmic (10 scale) genomic distance. Black points represent C to G mutations and red points C to T mutations. Top barplot shows the sum of mutations (density) across chromosomes.

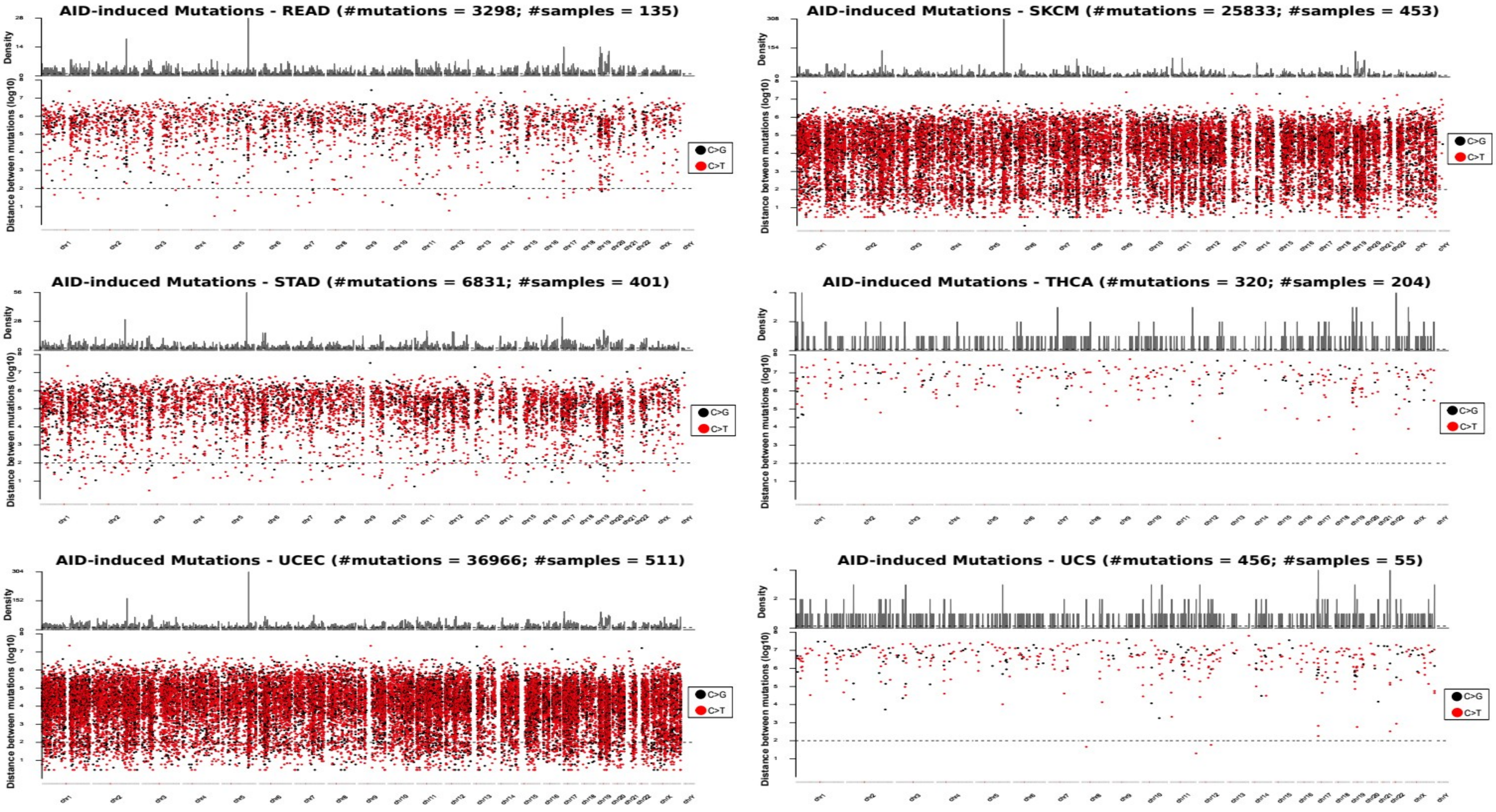

Supplementary Figure 8. AID mutations genomic distribution TCGA (Tumors READ, SKCM, STAD, THCA, UCEC, and UCS)
Rainfall plots of the AID mutations' distribution across chromosomes on TCGA samples by tumor types as a function of logarithmic (10 scale) genomic distance. Black points represent C to G mutations and red points C to T mutations. Top barplot shows the sum of mutations (density) across chromosomes.

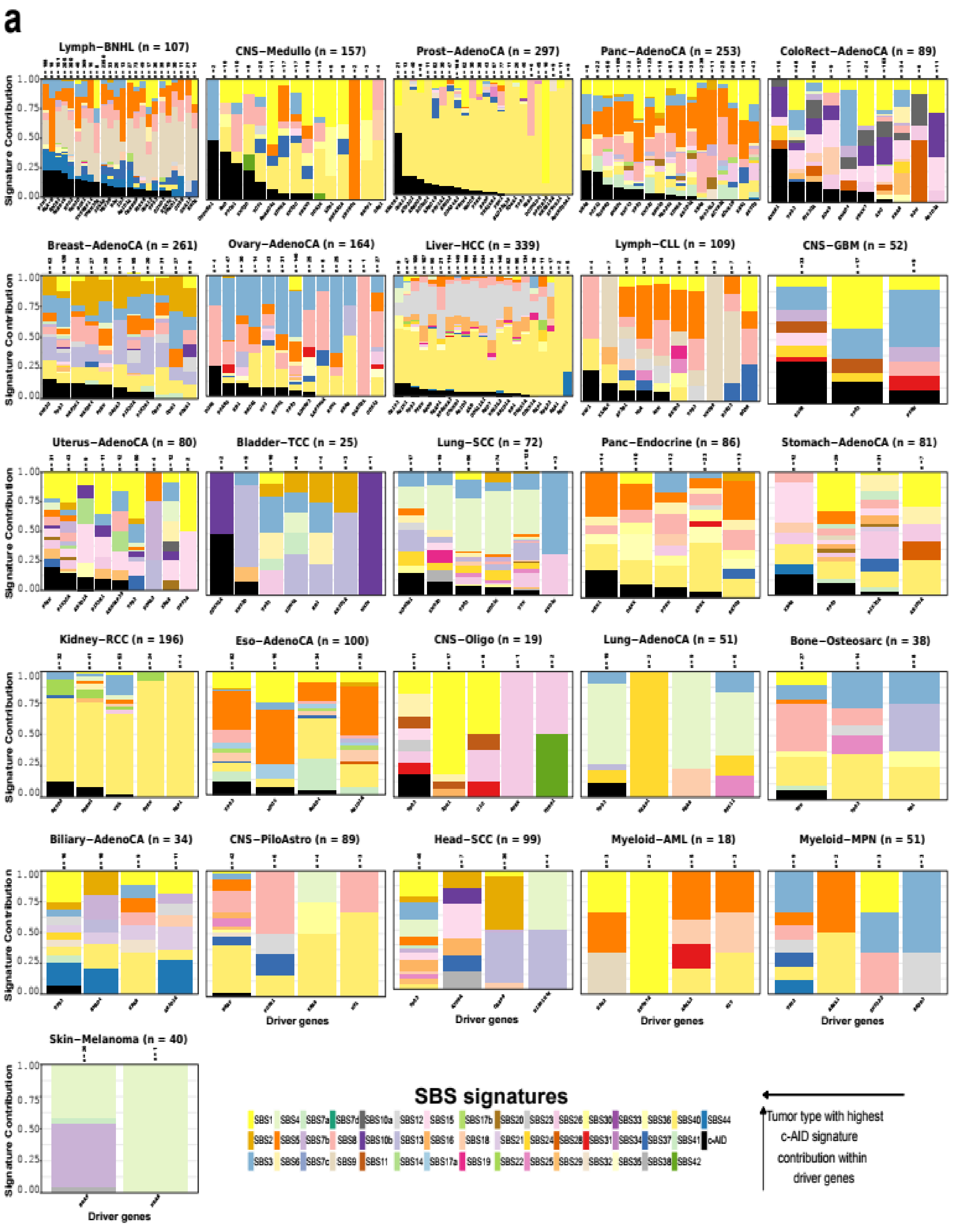
a
Lymph−BNHL (n = 107)
CNS−Medullo (n = 157)
Prost−AdenoCA (n = 297)
Panc−AdenoCA (n = 253)
ColoRect−AdenoCA (n = 89)
Breast−AdenoCA (n = 261)
Ovary−AdenoCA (n = 164)
Liver−HCC (n = 339)
Lymph−CLL (n = 109)
CNS−GBM (n = 52)
Uterus−AdenoCA (n = 80)
Bladder−TCC (n = 25)
Lung−SCC (n = 72)
Panc−Endocrine (n = 86)
Stomach−AdenoCA (n = 81)
Kidney−RCC (n = 196)
Eso−AdenoCA (n = 100)
CNS−Oligo (n = 19)
Lung−AdenoCA (n = 51)
Bone−Osteosarc (n = 38)
Biliary−AdenoCA (n = 34)
CNS−PiloAstro (n = 89)
Head−SCC (n = 99)
Myeloid−AML (n = 18)
Myeloid−MPN (n = 51)
Skin−Melanoma (n = 40)
Signature Contribution
0.00
0.25
0.50
0.75
1.00
Driver genes
SBS signatures
SBS1 SBS4 SBS7a SBS7d SBS10a SBS12 SBS15 SBS17b SBS20 SBS23 SBS26 SBS30 SBS33 SBS36 SBS40 SBS44
SBS2 SBS5 SBS7b SBS8 SBS10b SBS13 SBS16 SBS18 SBS21 SBS24 SBS28 SBS31 SBS34 SBS37 SBS41 c-AID
SBS3 SBS6 SBS7c SBS9 SBS11 SBS14 SBS17a SBS19 SBS22 SBS25 SBS29 SBS32 SBS35 SBS38 SBS42
Tumor type with highest c-AID signature contribution within driver genes

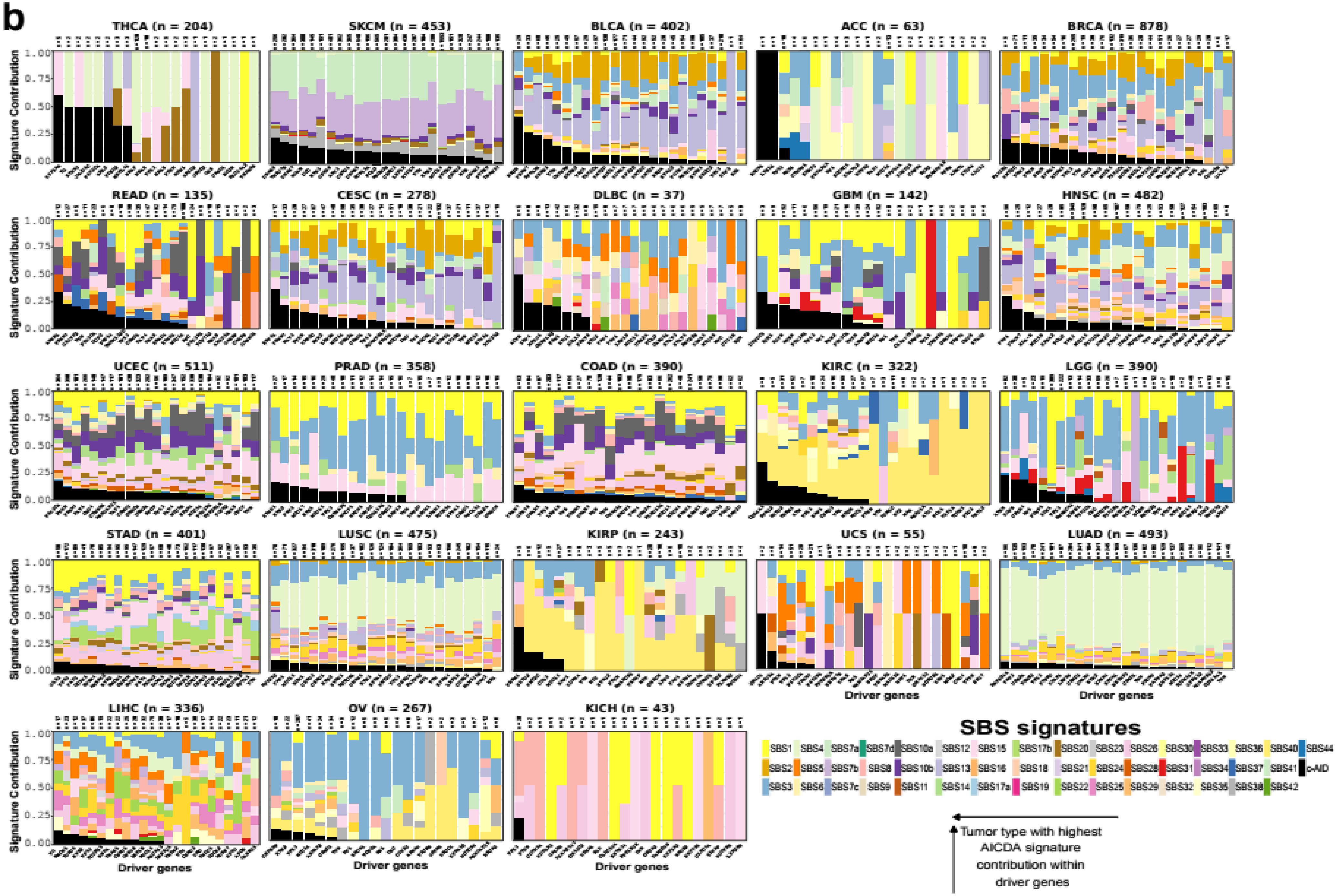
b
THCA (n = 204)
SKCM (n = 453)
BLCA (n = 402)
ACC (n = 63)
BRCA (n = 878)
READ (n = 135)
CESC (n = 278)
DLBC (n = 37)
GBM (n = 142)
HNSC (n = 482)
UCEC (n = 511)
PRAD (n = 358)
COAD (n = 390)
KIRC (n = 322)
LGG (n = 390)
STAD (n = 401)
LUSC (n = 475)
KIRP (n = 243)
UCS (n = 55)
LUAD (n = 493)
LIHC (n = 336)
OV (n = 267)
KICH (n = 43)
Signature Contribution
1.00
0.75
0.50
0.25
0.00
Driver genes
SBS signatures
SBS1
SBS2
SBS3
SBS4
SBS5
SBS6
SBS7a
SBS7b
SBS7c
SBS7d
SBS8
SBS9
SBS10a
SBS10b
SBS11
SBS12
SBS13
SBS14
SBS15
SBS16
SBS17a
SBS17b
SBS18
SBS19
SBS20
SBS21
SBS22
SBS23
SBS24
SBS25
SBS26
SBS28
SBS29
SBS30
SBS31
SBS32
SBS33
SBS34
SBS35
SBS36
SBS37
SBS38
SBS40
SBS41
SBS42
SBS44
c-AID
Tumor type with highest AICDA signature contribution within driver genes

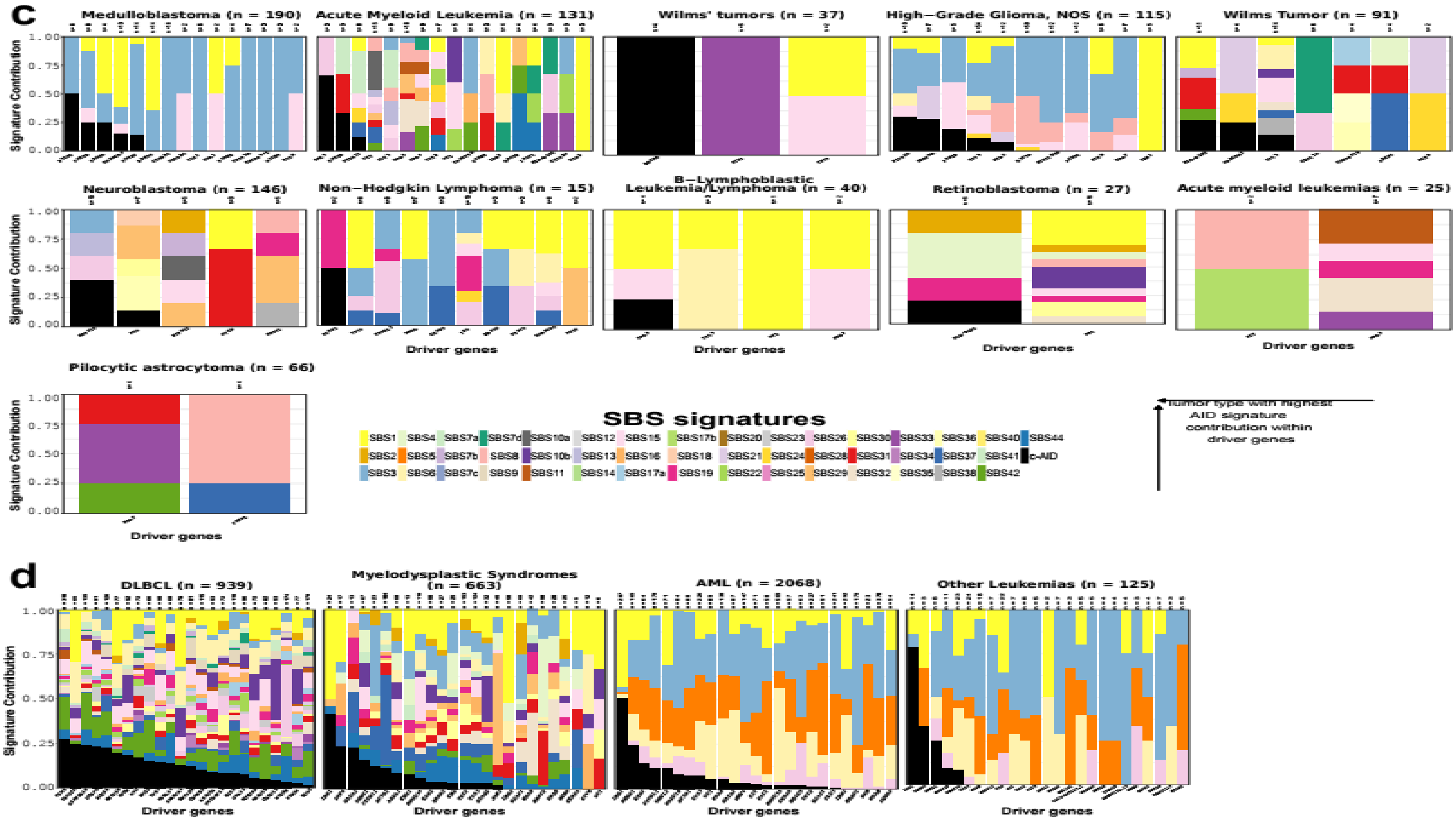

Supplementary Figure 9. AID mutations contribution on driver genes

Mutational signatures' contribution (COSMIC signatures and AID mutations) to driver genes on the ICGC (**a**), TCGA (**b**), Pediatric (**c**) and Hematological (**d**) cohorts across tumor types where black bars represent AID mutations.

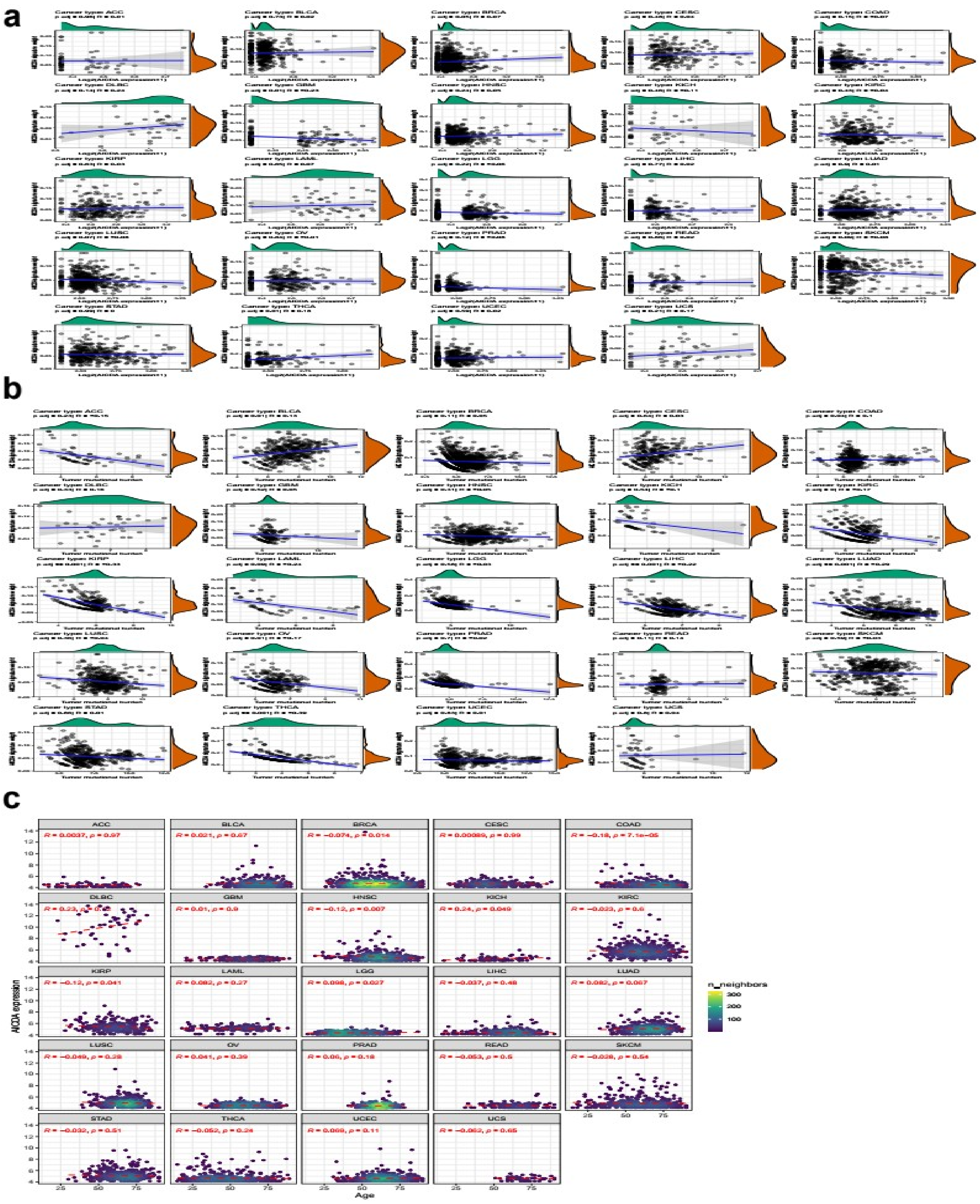

Supplementary Figure 10. *AICDA* expression or AID mutations correlations with other features (TCGA cohort)

Spearman correlations of *AICDA* expression with AID mutations (**a**) and age (**c**) or AID mutations with TMB (**b**).

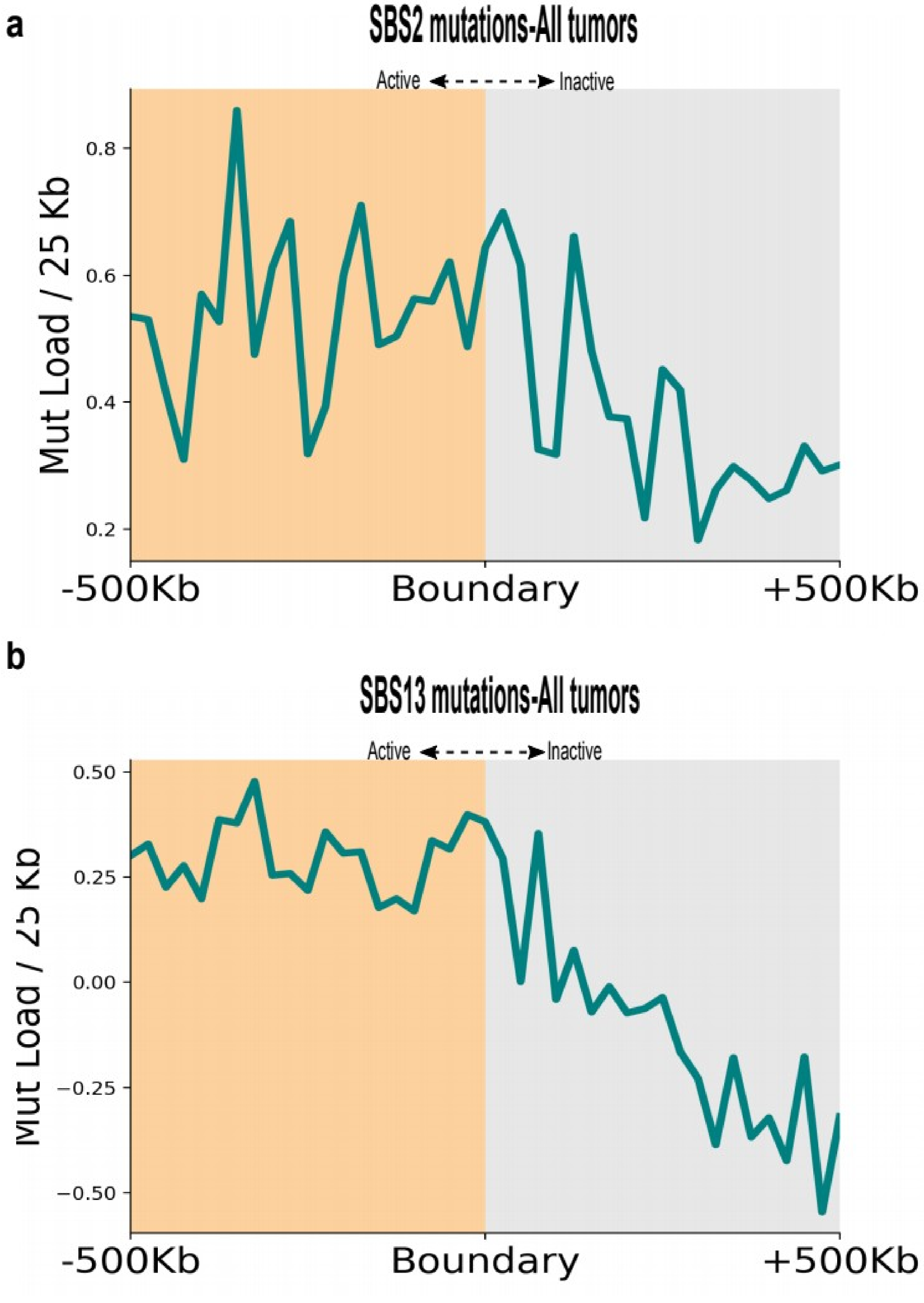

Supplementary Figure 11. APOBEC mutations interplay with TADs

Average profile of SBS2 (**a**) and SBS13 (**b**) somatic mutations accumulation in 2,775 cancer samples and replication timing across 500 kb of TAD boundaries delineating active to inactive domains. Related to Figure 4.

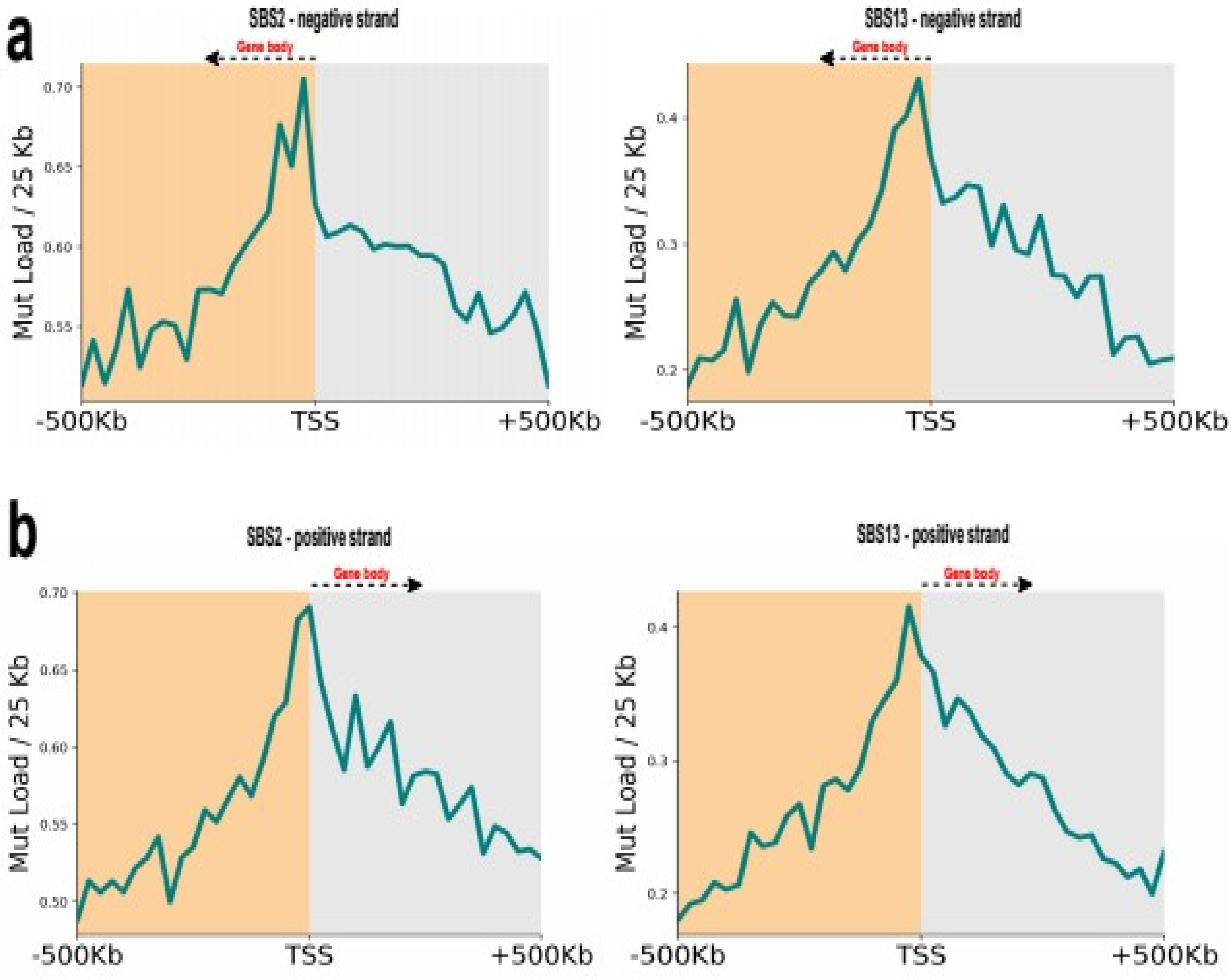

Supplementary Figure 12. APOBEC mutations interplay transcription directionality

a,**c** Average profiles of SBS2 or SBS13 induced mutations accumulation in 2,775 cancer samples across 500 kb of TSS for negative strand genes (top) or positive strand genes (bottom).

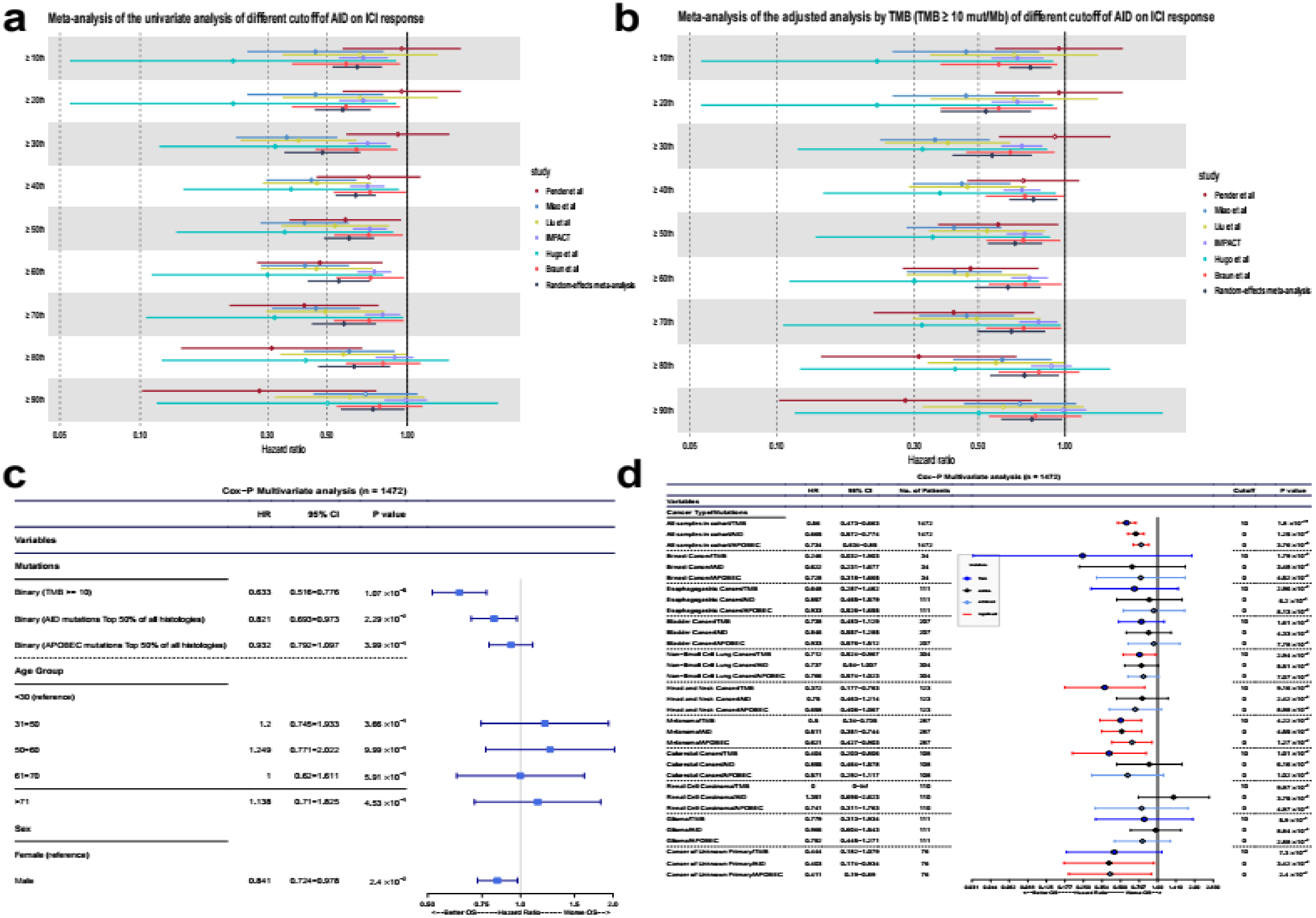

a
Meta-analysis of the univariate analysis of different cutoff of AID on ICI response
≥ 10th
≥ 20th
≥ 30th
≥ 40th
≥ 50th
≥ 60th
≥ 70th
≥ 80th
≥ 90th
Hazard ratio
study
Pender et al
Miao et al
Liu et al
IMPACT
Hugo et al
Braun et al
Random-effects meta-analysis
b
Meta-analysis of the adjusted analysis by TMB (TMB ≥ 10 mut/Mb) of different cutoff of AID on ICI response
c
Cox−P Multivariate analysis (n = 1472)
HR
95% CI
P value
Variables
Mutations
Binary (TMB >= 10)
Binary (AID mutations Top 50% of all histologies)
Binary (APOBEC mutations Top 50% of all histologies)
Age Group
<30 (reference)
31~50
50~60
61~70
>71
Sex
Female (reference)
Male
d
Cox−P Multivariate analysis (n = 1472)

Supplementary Figure 13. The impact of AID/APOBEC mutations on ICI response

Meta-analysis of the survival impact of the fraction of AID mutations in different studies. **a** Effect using all the deciles of the fractions of AID mutations at univariate level, the overall impact of AID with a better OS is present independently of the cut-off (relative to Figure 6A). **b** Using all the deciles of the fractions of AID mutations (adjusting every decile of fractions of AID mutations per TMB ≥ 10 mut / Mb), the overall impact of AID with a better OS is present independently of the cut-off. Assessment of the prognostic value of the fraction of AID mutations in the IMPACT study. **c**,**d** Assessment of the prognostic value of the fraction of AID mutations in the IMPACT study. Forest plot of a Cox model of either the global impact, after adjustment by TMB (≥10 mut/Mb), median APOBEC mutations, age and gender (**c**) or the impact by tumor subtype (**d**), relative to Figures 6B and 6C.

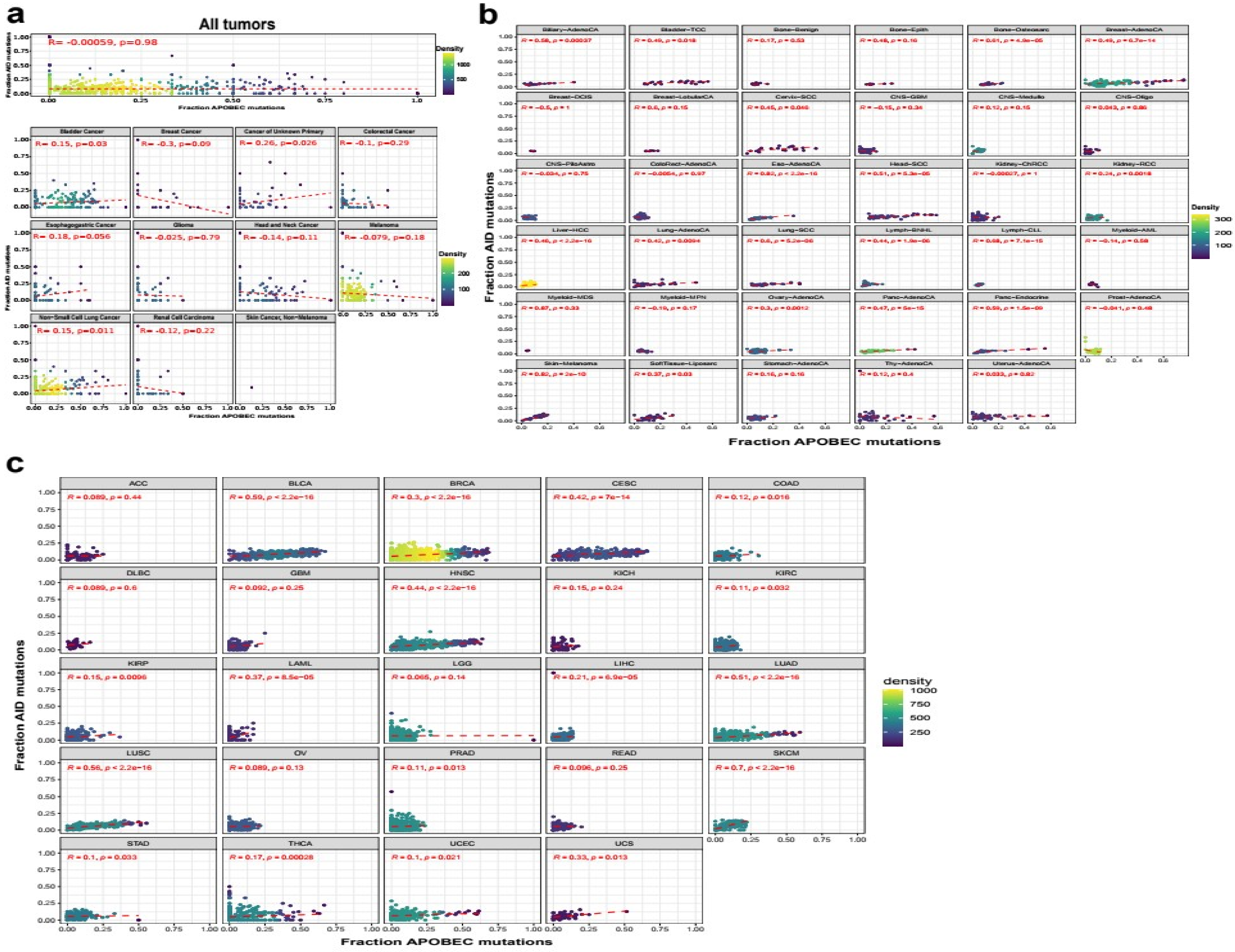
a
All tumors
R= -0.00059, p=0.98
Fraction AID mutations
Fraction APOBEC mutations
Density
Bladder Cancer
R= 0.15, p=0.03
Breast Cancer
R= -0.3, p=0.09
Cancer of Unknown Primary
R= 0.26, p=0.026
Colorectal Cancer
R= -0.1, p=0.29
Esophagogastric Cancer
R= 0.18, p=0.056
Glioma
R= -0.025, p=0.79
Head and Neck Cancer
R= -0.14, p=0.11
Melanoma
R= -0.079, p=0.18
Non-Small Cell Lung Cancer
R= 0.15, p=0.011
Renal Cell Carcinoma
R= -0.12, p=0.22
Skin Cancer, Non-Melanoma
b
Biliary−AdenoCA
Bladder−TCC
Bone−Benign
Bone−Epith
Bone−Osteosarc
Breast−AdenoCA
Breast−DCIS
Breast−LobularCA
Cervix−SCC
CNS−GBM
CNS−Medullo
CNS−Oligo
CNS−PiloAstro
ColoRect−AdenoCA
Eso−AdenoCA
Head−SCC
Kidney−ChRCC
Kidney−RCC
Liver−HCC
Lung−AdenoCA
Lung−SCC
Lymph−BNHL
Lymph−CLL
Myeloid−AML
Myeloid−MDS
Myeloid−MPN
Ovary−AdenoCA
Panc−AdenoCA
Panc−Endocrine
Prost−AdenoCA
Skin−Melanoma
SoftTissue−Liposarc
Stomach−AdenoCA
Thy−AdenoCA
Uterus−AdenoCA
Density
Fraction AID mutations
Fraction APOBEC mutations
c
ACC
R = 0.089, p = 0.44
BLCA
R = 0.59, p < 2.2e−16
BRCA
R = 0.3, p < 2.2e−16
CESC
R = 0.42, p = 7e−14
COAD
R = 0.12, p = 0.016
DLBC
R = 0.089, p = 0.6
GBM
R = 0.092, p = 0.25
HNSC
R = 0.44, p < 2.2e−16
KICH
R = 0.15, p = 0.24
KIRC
R = 0.11, p = 0.032
KIRP
R = 0.15, p = 0.0096
LAML
R = 0.37, p = 8.5e−05
LGG
R = 0.065, p = 0.14
LIHC
R = 0.21, p = 6.9e−05
LUAD
R = 0.51, p < 2.2e−16
LUSC
R = 0.56, p < 2.2e−16
OV
R = 0.089, p = 0.13
PRAD
R = 0.11, p = 0.013
READ
R = 0.096, p = 0.25
SKCM
R = 0.7, p < 2.2e−16
STAD
R = 0.1, p = 0.033
THCA
R = 0.17, p = 0.00028
UCEC
R = 0.1, p = 0.021
UCS
R = 0.33, p = 0.013
density
Fraction AID mutations
Fraction APOBEC mutations

Supplementary Figure 14. Correlation of AID mutations with APOBEC mutations
Correlation between the fraction of AID mutations and the fraction of APOBEC mutations in the IMPACT (**a**), ICGC (**b**) or TCGA (**c**) datasets.

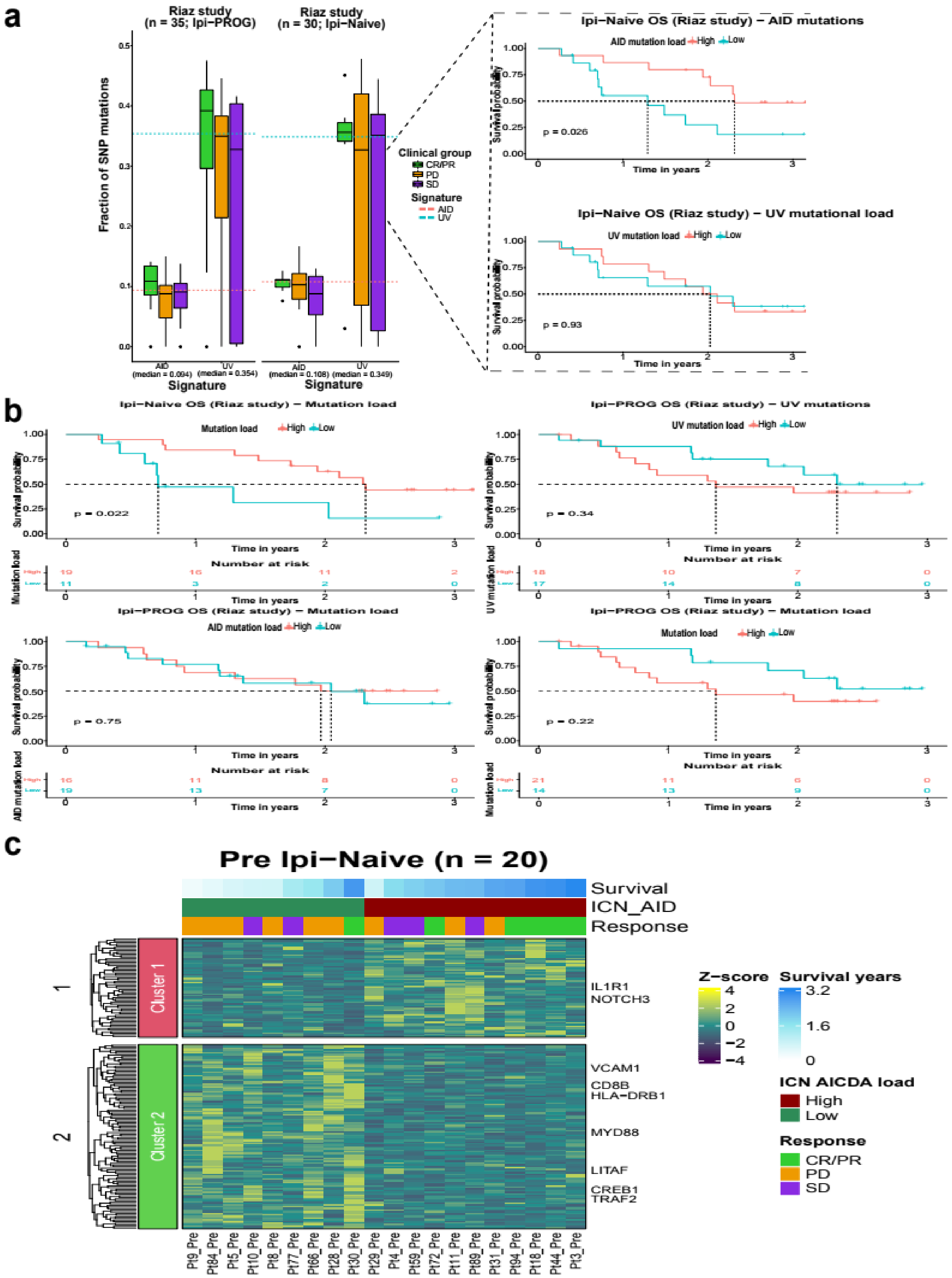
a
Riaz study (n = 35; Ipi−PROG)
Riaz study (n = 30; Ipi−Naive)
Fraction of SNP mutations
AID (median = 0.094)
UV (median = 0.354)
AID (median = 0.108)
UV (median = 0.349)
Signature
Clinical group
CR/PR
PD
SD
Signature
AID
UV
Ipi−Naive OS (Riaz study) − AID mutations
AID mutation load High Low
Survival probability
p = 0.026
Time in years
Ipi−Naive OS (Riaz study) − UV mutational load
UV mutation load High Low
p = 0.93
b
Ipi−Naive OS (Riaz study) − Mutation load
Mutation load High Low
p = 0.022
Number at risk
Ipi−PROG OS (Riaz study) − UV mutations
UV mutation load High Low
p = 0.34
Ipi−PROG OS (Riaz study) − Mutation load
AID mutation load High Low
p = 0.75
Ipi−PROG OS (Riaz study) − Mutation load
Mutation load High Low
p = 0.22
c
Pre Ipi−Naive (n = 20)
Survival
ICN_AID
Response
Cluster 1
Cluster 2
IL1R1
NOTCH3
VCAM1
CD8B
HLA−DRB1
MYD88
LITAF
CREB1
TRAF2
Z−score
Survival years
ICN AICDA load
High
Low
Response
CR/PR
PD
SD
Pt9_Pre
Pt84_Pre
Pt5_Pre
Pt10_Pre
Pt8_Pre
Pt77_Pre
Pt66_Pre
Pt28_Pre
Pt30_Pre
Pt29_Pre
Pt4_Pre
Pt59_Pre
Pt72_Pre
Pt11_Pre
Pt89_Pre
Pt31_Pre
Pt94_Pre
Pt18_Pre
Pt44_Pre
Pt3_Pre

Supplementary Figure 15. AID-related neoepitopes role in ICI response (Riaz et al cohort)

a AID and UV related mutations are higher within responders (Complete-response/Partial-Response, CR/PR) compared to non-responder (stable disease [SD] or progrssive disease [PD]) in both Ipi-Prog and Ipi-Naive cohorts (left, data are presented as median and interquartile range) but OS only associates with AID mutations within Ipi-Naive patients (right). Centre line represents the median values; error bars represent the upper and lower quartiles and whiskers define the minimal and maximum values. **b** OS prediction within Ipi-Naive patients using mutational load (top left) or within Ipi-Progressive patients using UV mutational load (top right), AID mutational load (bottom left) or global mutational load (bottom right). **c** Heatmap and hierarchical clustering of DEGs between high and low ICN AID load within pre-therapy Ipi-Naive patients.

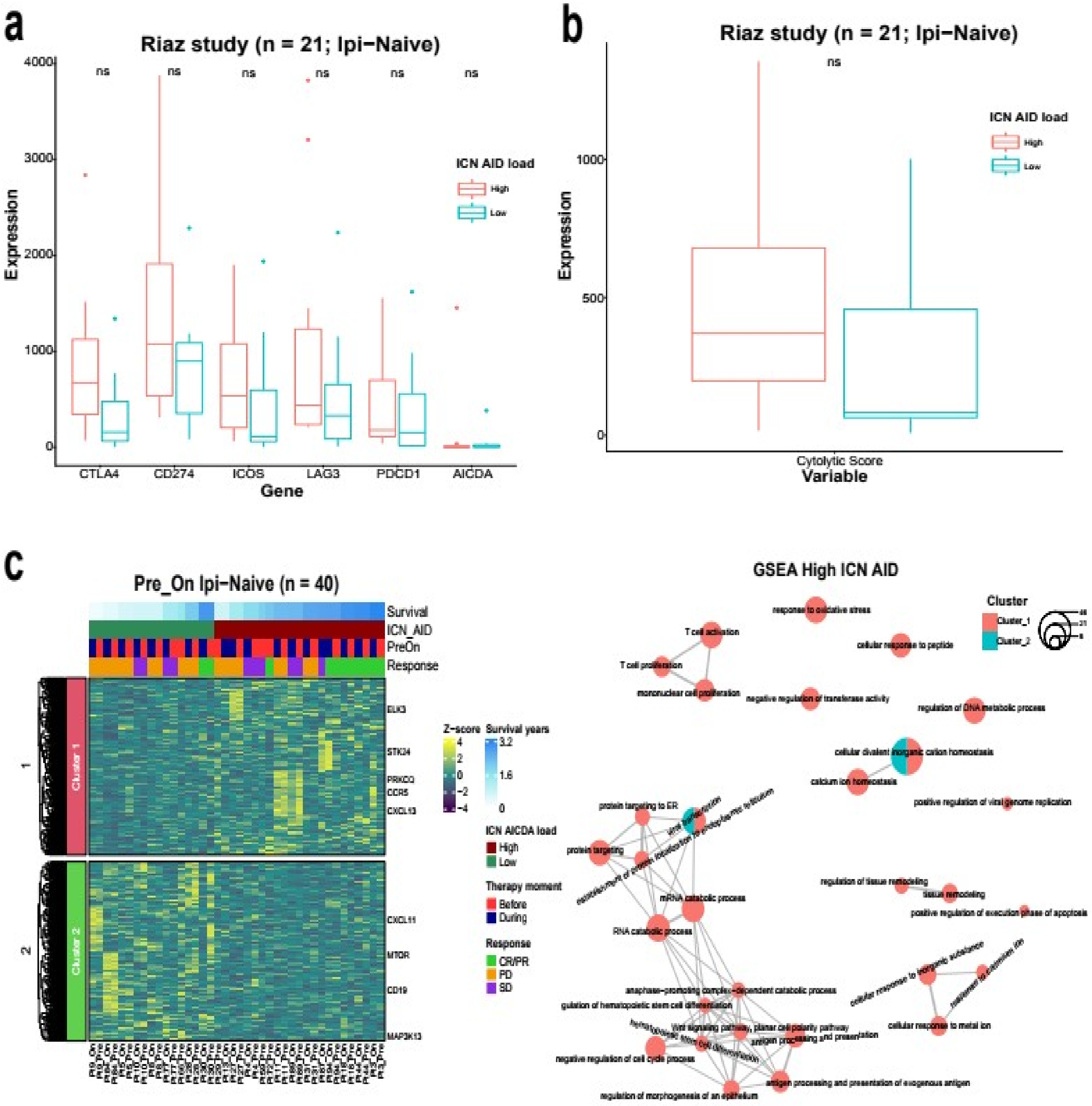

Supplementary Figure 16. AID-related neoepitopes role in ICI response (Riaz et al cohort)

a, **b** Boxplots comparison showing increased expression trend of either inhibitory immune checkpoint molecules or cytolytic activity, respectively (Wilcoxon-test). **c** Heatmap and hierarchical clustering of DEGs between pre and on-therapy grouped according to high/low ICN AID load (top). GO enrichment analysis ($q < 0.05$) of the gene clusters found in the heatmap.

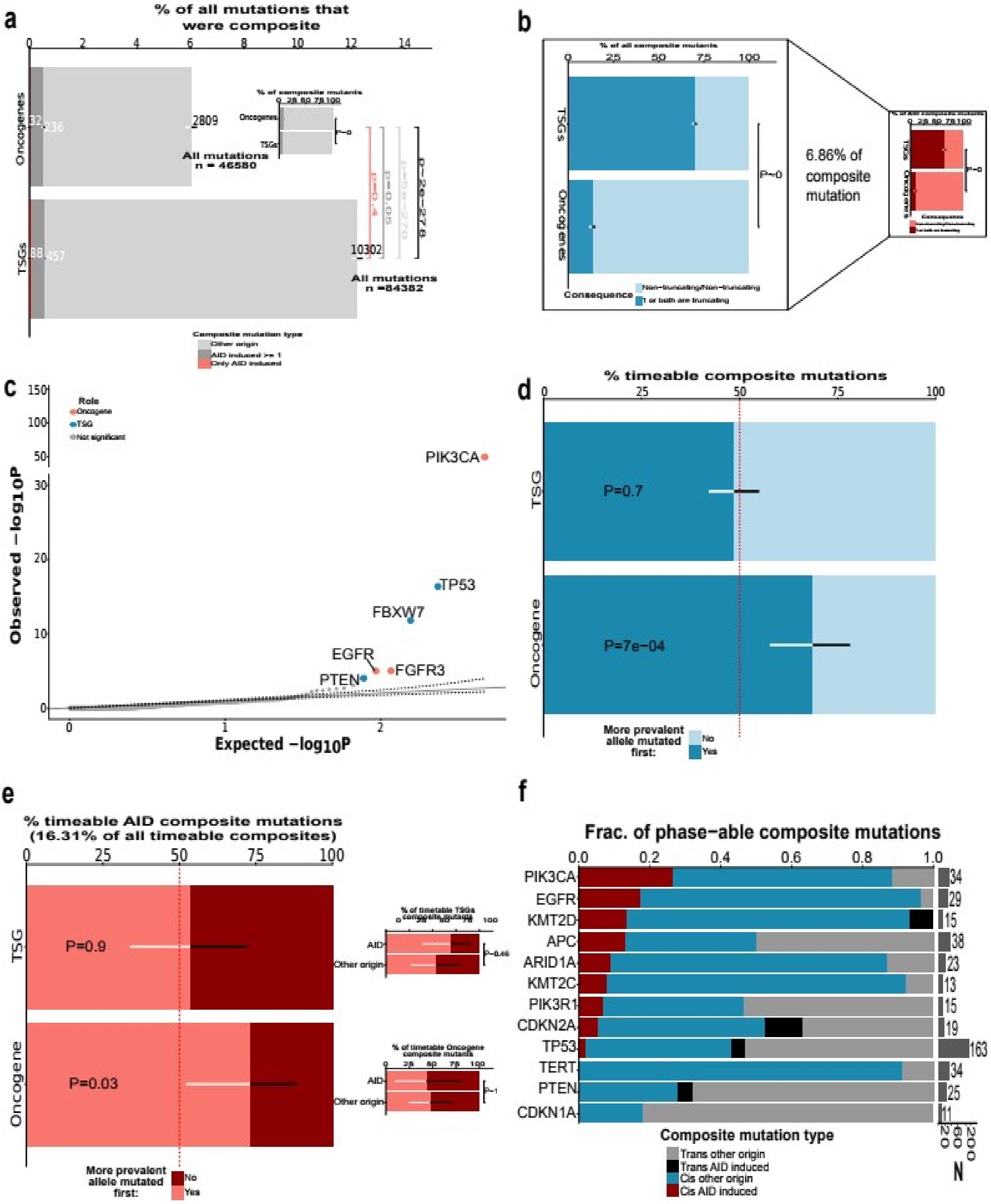
a
% of all mutations that were composite
Oncogenes
TSGs
2809
10302
All mutations n = 46580
All mutations n =84382
% of composite mutants
Composite mutation type
Other origin
AID induced >= 1
Only AID induced
b
% of all composite mutants
TSGs
Oncogenes
P~0
6.86% of composite mutation
Consequence
Non-truncating/Non-truncating
1 or both are truncating
c
Role
Oncogene
TSG
Not significant
PIK3CA
TP53
FBXW7
EGFR
FGFR3
PTEN
Observed −log10P
Expected −log10P
d
% timeable composite mutations
TSG
Oncogene
P=0.7
P=7e−04
More prevalent allele mutated first:
No
Yes
e
% timeable AID composite mutations (16.31% of all timeable composites)
TSG
Oncogene
P=0.9
P=0.03
% of timetable TSGs composite mutants
% of timetable Oncogene composite mutants
AID
Other origin
More prevalent allele mutated first:
No
Yes
f
Frac. of phase−able composite mutations
PIK3CA 34
EGFR 29
KMT2D 15
APC 38
ARID1A 23
KMT2C 13
PIK3R1 15
CDKN2A 19
TP53 163
TERT 34
PTEN 25
CDKN1A 11
Composite mutation type
Trans other origin
Trans AID induced
Cis other origin
Cis AID induced
N

Supplementary Figure 17. AID mutations role in composite mutations

a Percentage of total mutations (n = 130,962) that were composite by cancer gene function and composite mutation type (Two sided two-sample Z-test for equal proportions; color indicates the composite mutation type being compared; numbers on bars indicate absolute numbers of each mutation type). Inside plot represents the relative contribution of at least one AID mutation within the composite or other origin to the composite mutations. **b** Percentage of composite mutations by cancer gene function and consequence (Two sided Fisher's exact test) in global composite mutations (n = 6,681, left panel) or only AID composite mutations (n = 472, right panel). **c** Significant enrichment for AID composite mutations in cancer genes (FDR-adjusted P values from one-sided binomial test for enrichment, n=29,461). **d** Temporal order of acquisition of global composite mutations by cancer gene function using clonality and allelic configuration (Two-sided binomial test). **e** Temporal order of acquisition of AID composite mutations by cancer gene function (n = 54 evaluable AID composites) using clonality and allelic configuration. Two-sided binomial test (left panel). Relative contribution of at least one AID mutation within the composite or other origin to each cancer gene function (Two sided Fisher's exact test, right panel). **f** Phase-able composite mutations by composite mutation type on different genes (left panel). Cases with phase-able composite mutations (right panel).

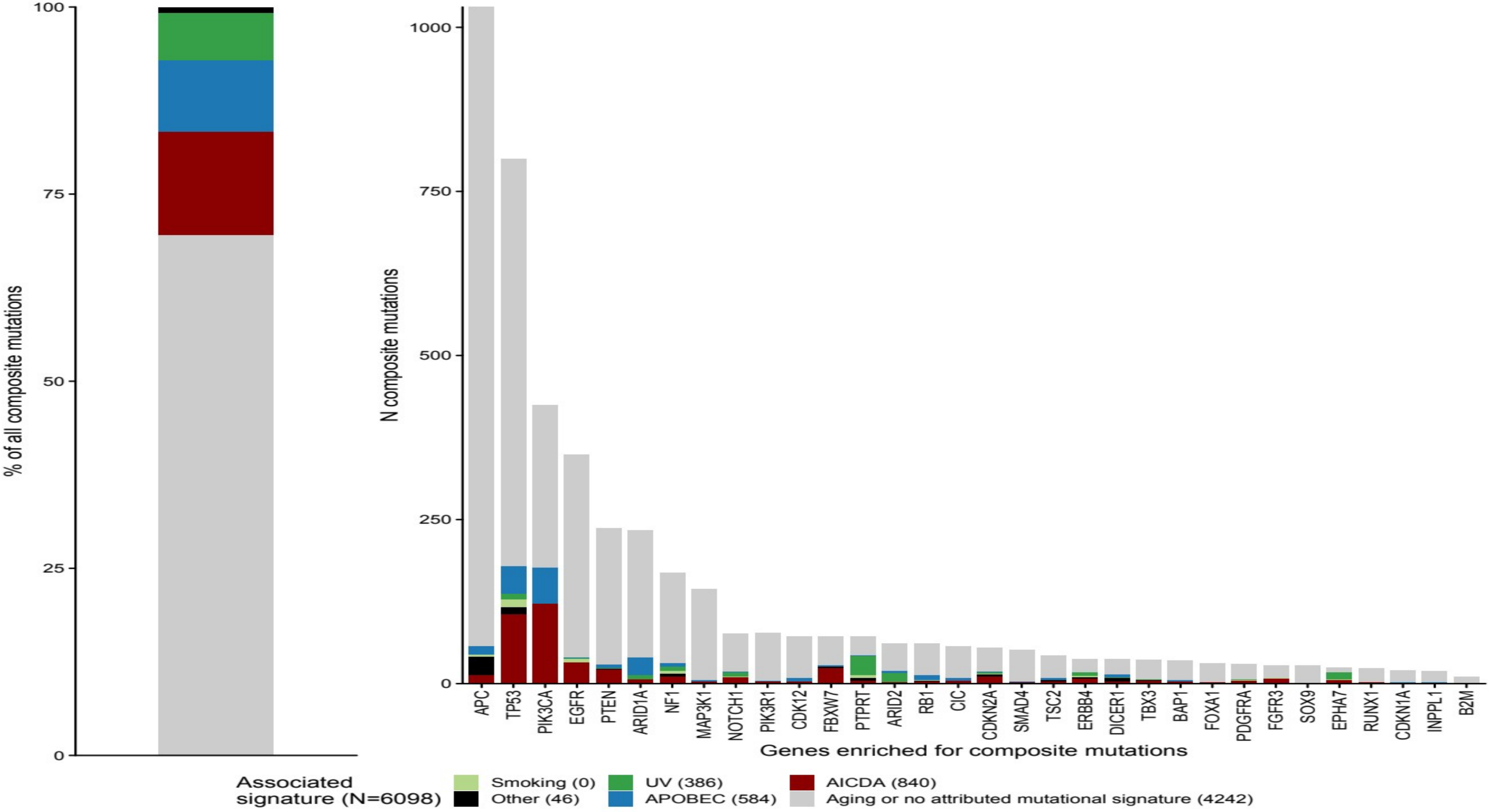

Supplementary Figure 18. Composite mutations and mutational signatures

Mutational signatures' contribution to composite mutations globally (left) and per driver gene (right).

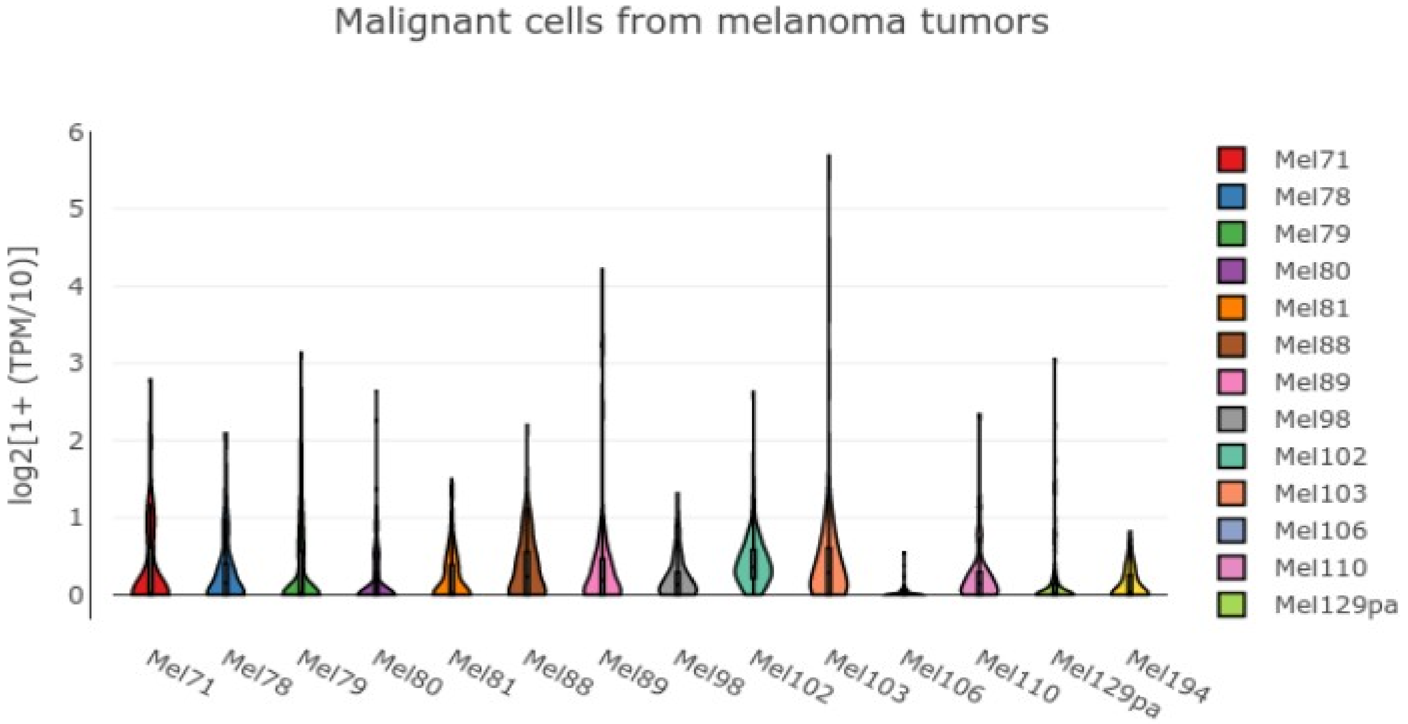

Supplementary Figure 19. *AICDA* expression in melanoma malignant cells by single cell RNA-seq.
Violin plot showing the expression of AICDA of different subpopulations of malignant melanoma cells from Jerby-Arnon et al. (2018). Centre line represents the median values; error bars represent the upper and lower quartiles and whiskers define the range within the 1.5 interquartile range.

Supplementary Tables

KEY RESOURCES TABLE

REAGENT or RESOURCE	SOURCE (DOI)	IDENTIFIER
**Biological Samples**		
TCGA RNA-Seq data (read counts)	NCBI GEO GSE62944 (/10.1093/bioinformatics/btv377)	https://www.ncbi.nlm.nih.gov/geo/query/acc.cgi?acc=GSE62944
TCGA Mutation (SNV) Data	The Cancer Genome Atlas Research Network	https://api.gdc.cancer.gov/data/1c8cfe5f-e52d-41ba-94da-f15ea1337efc
TCGA Copy number variations (CNV) Data	The Cancer Genome Atlas Research Network	http://api.gdc.cancer.gov/data/00a32f7a-c85f-4f86-850d-be53973cbc4d
TCGA Clinical data	The Cancer Genome Atlas Research Network	https://api.gdc.cancer.gov/data/1b5f413e-a8d1-4d10-92eb-7c4ae739ed81
TCGA immune related data	The Cancer Genome Atlas Research Network	https://cavei.github.io/example-datasets/panCancerAnnotation.RData
TCGA viral reads per million reads mapped to the human genome	[1] (10.1016/j.cell.2014.12.033)	https://www.cell.com/cms/10.1016/j.cell.2014.12.033/attachment/02c50887-85ca-46bd-ae9a-915ccc36bbf4/mmc2.xlsx
TCGA predicted neoepitopes	[1] (10.1016/j.cell.2014.12.033)	https://www.cell.com/cms/10.1016/j.cell.2014.12.033/attachment/603562c6-566b-4891-b940-7134b9f7367b/mmc3.txt
TCGA HLA haplotyping	[2] (10.1158/2326-6066.CIR-19-0464)	https://cancerimmunolres.aacrjournals.org/highwire/filestream/38473/field_highwire_adjunct_files/1/224371_2_supp_5920266_q2tjr3.xlsx
International Cancer Genome Consortium - The PanCancer Analysis of Whole Genomes (PCAWG) Mutation (SNV) data	ICGC Data Portal	https://dcc.icgc.org/releases/PCAWG/consensus_snv_indel/final_consensus_snv_indel_pa sso nly_icgc.pu blic.tgz
International Cancer Genome Consortium - PCAWG Copy number alterations (CNA)	ICGC Data Portal	https://dcc.icgc.org/releases/PCAWG/consensus_cnv/consensus.2 0170119.so

		matic.cna.ic gc.controlle d.tar.gz
International Cancer Genome Consortium - PCAWG RNA expression data	ICGC Data Portal	https://dcc.icgc.org/api/v1/download?fn=/PCAWG/transcriptome/gene_expression/tophat_star_fpkm_uq.v2_aliquot_gl.tsv.gz
International Cancer Genome Consortium - PCAWG purity and ploidy calls	ICGC Data Portal	https://dcc.icgc.org/releases/PCAWG/consensus_cnv/consensus.20170217.purity.ploidy.txt.gz
International Cancer Genome Consortium - PCAWG MSI status	ICGC Data Portal	https://dcc.icgc.org/releases/PCAWG/msi/MS_analysis.PCAWG_release_v1.RIKEN.xlsx
International Cancer Genome Consortium - PCAWG donor clinical data	ICGC Data Portal	https://dcc.icgc.org/releases/PCAWG/clinical_and_histologypcawg_spe cimen_hist ology_August2016_v9.xlsx
ENCODE - Replication timing data of 11 cell lines	NCBI GEO GSE34399	https://www.ncbi.nlm.nih.gov/geo/query/acc.cgi?acc=GSE34399
Duke Cancer Institute and Center for Genomic and Computational Biology - DLBCL mutations	cBioPortal (10.1016/j.cell.2017.09.027)	https://www.cbioportal.org/study/summary?id=dlbcl_duke_2017
Oregon Health & Science University - AML and MDS mutations	cBioPortal (10.1038/s41586-018-0623-z)	https://www.cbioportal.org/study/summary?id=aml_ohsu_2018
The University of Tokyo - MDS mutations	cBioPortal (10.1038/nature10496)	https://www.cbioportal.org/study/summary?id=mds_tokyo_2011
Cambridge Institute for Medical Research - MDS mutations	cBioPortal (10.1056/NEJMoa1312542)	https://www.cbioportal.org/study/summary?id=mpn_cimr_2013
Washington University School of Medicine, and McDonnell Genome Institute - AML and MDS mutations	cBioPortal (10.1056/NEJMoa1605949)	https://www.cbioportal.org/study/summary?id=mnm_washu_2016
St. Jude Children's Research Hospital - ALL mutations	cBioPortal (10.1038/ng.2532)	https://www.cbioportal.org/study/summary?id=all_stjude_2013

St. Jude Children's Research Hospital - ALL mutations	cBioPortal (10.1038/ng.3230)	https://www.cbioportal.org/study/summary?id=all_stjude_2015
St. Jude Children's Research Hospital - ALL mutations	cBioPortal (10.1038/ng.3691)	https://www.cbioportal.org/study/summary?id=all_stjude_2016
Broad Institute of MIT - Multiple Myeloma mutations	cBioPortal (10.1016/j.ccr.2013.12.015)	https://www.cbioportal.org/study/summary?id=mm_broad
Broad Institute of MIT - CLL mutations	cBioPortal (10.1038/nature15395)	https://www.cbioportal.org/study/summary?id=cll_broad_2015
Broad Institute of MIT - CLL mutations	cBioPortal (10.1016/j.cell.2013.01.019)	https://www.cbioportal.org/study/summary?id=lcll_broad_2013
Oviedo University (IUOPA) - CLL mutations	cBioPortal (10.1038/ng.1032)	https://www.cbioportal.org/study/summary?id=cllsll_icgc_2011
Oviedo University (IUOPA) - CLL mutations	cBioPortal (10.1038/nature14666)	https://www.cbioportal.org/study/summary?id=cll_iuopa_2015
German Cancer Research Center (DKFZ) - Pediatric pan-cancer mutations	cBioPortal (10.1038/nature25480)	https://www.cbioportal.org/study/summary?id=pediatric_dkfz_2017
TARGET - Pediatric Neuroblastoma - mutations	cBioPortal (10.1038/nature25480)	https://www.cbioportal.org/study/summary?id=nbl_target_2018_pub
TARGET - Pediatric AML - mutations	cBioPortal (10.1038/nature25480)	https://www.cbioportal.org/study/summary?id=aml_target_2018_pub
TARGET - Pediatric Wilm's tumors - mutations	cBioPortal (10.1038/nature25480)	https://www.cbioportal.org/study/summary?id=wt_target_2018_pub
Chromatin state data	[3] (10.1038/nature12213)	https://static-content.springer.com/esm/art%3A10.1038%2Fnature12213/MediaObjects/41586_2013_BFnature12213_MOESM256_ESM.xls
Duke and DAC blacklisted regions	UCSC Browser	http://genome.ucsc.edu/cgi-bin/hgTrackUi?hgsid=876251189_lEwmtar4Jcr1MJgh92cL5gL9Anaa&g=wgEncodeDacMapabilityConsensus

		Excludable
“CRG Alignability 36” track30	UCSC Browser	https://genome.ucsc.edu/cgi-bin/hgTrackUi?db=hg18&g=wgEncodeMapability
GC skew (R- loops) genomic coordinates data	[4] (10.1016/j.molcel.2012.01.017)	https://www.cell.com/cms/10.1016/j.molcel.2012.01.017/attachment/2cc58e81-606a-47fe-9109-69717cc4baa3/mmc2.zip
Memorial Sloan Kettering Cancer Center (MSKCC) - Composite mutations	cBioPortal (10.1038/s41586-020-2315-8)	http://download.cbioportal.org/composite_mutations_maf.txt.gz https://github.com/taylor-lab/composite-mutations/tree/master/data
Pender et al ICI study	[5] (10.1158/1078-0432.CCR-20-1163)	https://www.bcgsc.ca/downloads/immunoPOG/
MSK-IMPACT ICI study	[6] (10.1038/s41588-018-0312-8)	http://www.cbioportal.org/study?id=tmb_mskcc_2018
Miao et al ICI study	[7] (10.1038/s41588-018-0200-2)	https://static-content.springer.com/esm/art%3A10.1038%2Fs41588-018-0200-2/MediaObjects/41588_2018_200_MOESM6_ESM.txt
Liu et al ICI study	[8] (10.1038/s41591-019-0654-5)	https://www.nature.com/articles/s41591-020-0839-y#MOESM2
Hugo et al ICI study	[9] 10.1016/j.cell.2016.02.065)	https://www.ncbi.nlm.nih.gov/pmc/articles/PMC4808437/bin/NIHMS765463-supplement-5.xlsx
Braun et al ICI study	[10] (10.1038/s41591-020-0839-y)	https://www.ncbi.nlm.nih.gov/pmc/articles/PMC6898788/bin/41591_2019_654_MOESM2_ESM.xlsx
Riaz et al ICI study - mutations, predicted neoepitopes, RNA counts matrix and clinical data	[11] (10.1016/j.cell.2017.09.028)	https://github.com/riazn/bms038_analysis/tree/master/data
Amin et al - Canine Glioma Mutations and clinical data	[12] (10.1016/j.ccell.2020.01.004)	https://github.com/TheJacksonLaboratory/canineglioma

Gardner et al - Canine Osteosarcoma Mutations and clinical data	[13] (10.1038/s42003-019-0487-2)	https://static-content.springer.com/esm/art%3A10.1038%2Fs42003-019-0487-2/MediaObjects/42003_2019_487_MOESM12_ESM.xlsx
Wong et al - Canine Melanoma mutations and clinical data	[14] (10.1038/s41467-018-08081-1)	https://static-content.springer.com/esm/art%3A10.1038%2Fs41467-018-08081-1/MediaObjects/41467_2018_8081_MOESM5_ESM.xlsx
**Software and Algorithms**		
Palimpsest 2.0.0	[15]	https://github.com/FunGeST/Palimpsest
ABSOLUTE 1.0.6	[16]	https://github.com/ShixiangWang/DoAbsolute
dndscv 0.0.1.0	[17]	https://github.com/im3sanger/dndscv/
Maftools 2.6.0	[18]	https://github.com/PoisonAlien/maftools
e1071 1.7-5	N/A	https://CRAN.R-project.org/package=e1071
Lawstat 3.4	[19]	https://CRAN.R-project.org/package=lawstat
BeedTools 2.26.0	[20]	https://github.com/arq5x/bedtools2
PRIME 1.0	[21]	https://github.com/GfellerLab/PRIME
DESeq2 1.30.1	[22]	https://github.com/mikelove/DESeq2
ComplexHeatmap 2.6.2	[23]	https://github.com/jokergoo/ComplexHeatmap
Circlize 0.4.12	[24]	https://github.com/jokergoo/circlize
clusterProfiler 3.18.1	[25]	https://github.com/YuLab-SMU/clusterProfiler
survival 3.2.11	[26]	https://CRAN.R-project.org/package=survival
GSVA 1.38.2	[27]	https://github.com/rcastelo/GSVA

karyoploteR 1.16.0	[28]	https://github.com/bernatgel/karyoploteR
MutationalPatterns 3.0.1	[29]	https://github.com/UMCUGenetics/MutationalPatterns
Meta 4.18-1	[30]	https://cran.r-project.org/web/packages/meta/index.html
ggforestplot 0.1.0	N/A	https://github.com/NightingaleHealth/ggforestplot
cancereffectsizeR 2.1.3	[31]	https://github.com/Townsend-Lab-Yale/cancereffectsizeR
Biostrings 2.60.0	N/A	https://bioconductor.org/packages/release/bioc/html/Biostrings.html
MutationalDistribution	[32]	https://github.com/kcakdemir/MutationalDistribution
Seurat 4.0.2	[33]	https://github.com/satijalab/seurat/
SigProfilerSimulator 1.1	[34]	https://github.com/AlexandrovLab/SigProfilerSimulator

Supplementary Table 1. Cohorts characteristics. Number of samples per tumor type included within each cohort. The subgroup column indicates in which type of analysis the samples were used.

Subgroup	Tumor type	Samples	Cohort	Fraction
Mutations/CNV	BTC	34	ICGC	1.22522522522523
Mutations/CNV	BLCA	23	ICGC	0.828828828828829
Mutations/CNV	BBT	16	ICGC	0.576576576576577
Mutations/CNV	Bone-Epith	10	ICGC	0.36036036036036
Mutations/CNV	OGS	38	ICGC	1.36936936936937
Mutations/CNV	Breast-AdenoCA	205	ICGC	7.38738738738739
Mutations/CNV	Breast-DCIS	3	ICGC	0.108108108108108
Mutations/CNV	Breast-LobularCA	7	ICGC	0.252252252252252
Mutations/CNV	CESC	20	ICGC	0.720720720720721
Mutations/CNV	GBM	41	ICGC	1.47747747747748
Mutations/CNV	CNS-Medullo	157	ICGC	5.65765765765766
Mutations/CNV	CNS-Oligo	18	ICGC	0.648648648648649
Mutations/CNV	CNS-PiloAstro	89	ICGC	3.20720720720721
Mutations/CNV	COAD	60	ICGC	2.16216216216216
Mutations/CNV	ESCA	99	ICGC	3.56756756756757
Mutations/CNV	HNSC	56	ICGC	2.01801801801802
Mutations/CNV	KICH	45	ICGC	1.62162162162162
Mutations/CNV	KIRC	165	ICGC	5.94594594594595
Mutations/CNV	LIHC	336	ICGC	12.1081081081081
Mutations/CNV	LUAD	38	ICGC	1.36936936936937
Mutations/CNV	LUSC	49	ICGC	1.76576576576577
Mutations/CNV	Lymph-BNHL	107	ICGC	3.85585585585586
Mutations/CNV	Lymph-CLL	100	ICGC	3.6036036036036
Mutations/CNV	LAML	18	ICGC	0.648648648648649
Mutations/CNV	Myeloid-MDS	3	ICGC	0.108108108108108
Mutations/CNV	Myeloid-MPN	51	ICGC	1.83783783783784
Mutations/CNV	Ovary-AdenoCA	115	ICGC	4.14414414414414
Mutations/CNV	PAAD	246	ICGC	8.86486486486486
Mutations/CNV	Panc-Endocrine	86	ICGC	3.0990990990991
Mutations/CNV	PRAD	293	ICGC	10.5585585585586
Mutations/CNV	SKCM	38	ICGC	1.36936936936937
Mutations/CNV	STS	34	ICGC	1.22522522522523
Mutations/CNV	STAD	76	ICGC	2.73873873873874
Mutations/CNV	THCA	48	ICGC	1.72972972972973
Mutations/CNV	UCEC	51	ICGC	1.83783783783784
Expression	BTC	18	ICGC	0.648648648648649
Expression	BLCA	20	ICGC	0.720720720720721
Expression	Breast-AdenoCA	82	ICGC	2.95495495495495
Expression	CESC	20	ICGC	0.720720720720721
Expression	GBM	23	ICGC	0.828828828828829
Expression	CNS-Oligo	15	ICGC	0.540540540540541
Expression	COAD	52	ICGC	1.87387387387387
Expression	ESCA	7	ICGC	0.252252252252252

Expression	HNSC	36	ICGC	1.2972972972973
Expression	KICH	40	ICGC	1.44144144144144
Expression	KIRC	113	ICGC	4.07207207207207
Expression	LIHC	95	ICGC	3.42342342342342
Expression	LUAD	36	ICGC	1.2972972972973
Expression	LUSC	46	ICGC	1.65765765765766
Expression	Lymph-BNHL	105	ICGC	3.78378378378378
Expression	Lymph-CLL	69	ICGC	2.48648648648649
Expression	Ovary-AdenoCA	84	ICGC	3.02702702702703
Expression	PAAD	71	ICGC	2.55855855855856
Expression	PRAD	16	ICGC	0.576576576576577
Expression	SKCM	35	ICGC	1.26126126126126
Expression	STS	29	ICGC	1.04504504504505
Expression	STAD	28	ICGC	1.00900900900901
Expression	THCA	42	ICGC	1.51351351351351
Expression	UCEC	48	ICGC	1.72972972972973
Expression	ACC	79	TCGA	0.868036479507746
Expression	BLCA	408	TCGA	4.48302384353368
Expression	BRCA	1094	TCGA	12.0206570706516
Expression	CESC	304	TCGA	3.34029227557411
Expression	COAD	456	TCGA	5.01043841336117
Expression	DLBC	48	TCGA	0.527414569827491
Expression	GBM	161	TCGA	1.76903636962971
Expression	HNSC	502	TCGA	5.51587737611252
Expression	KICH	66	TCGA	0.725195033512801
Expression	KIRC	533	TCGA	5.85649928579277
Expression	KIRP	290	TCGA	3.18646302604109
Expression	LAML	178	TCGA	1.95582902977695
Expression	LGG	513	TCGA	5.63674321503132
Expression	LIHC	371	TCGA	4.07647511262499
Expression	LUAD	516	TCGA	5.66970662564553
Expression	LUSC	501	TCGA	5.50488957257444
Expression	OV	423	TCGA	4.64784089660477
Expression	PRAD	497	TCGA	5.46093835842215
Expression	READ	165	TCGA	1.812987583782
Expression	SKCM	469	TCGA	5.15327985935611
Expression	STAD	420	TCGA	4.61487748599055
Expression	THCA	505	TCGA	5.54884078672673
Expression	UCEC	545	TCGA	5.98835292824964
Expression	UCS	57	TCGA	0.626304801670146
Mutations	ACC	79	TCGA	0.868036479507746
Mutations	BLCA	407	TCGA	4.47203603999561
Mutations	BRCA	1015	TCGA	11.1526205911438
Mutations	CESC	286	TCGA	3.1425118118888
Mutations	COAD	402	TCGA	4.41709702230524
Mutations	DLBC	37	TCGA	0.406548730908691
Mutations	GBM	156	TCGA	1.71409735193935
Mutations	HNSC	495	TCGA	5.43896275134601

Mutations	KICH	66	TCGA	0.725195033512801
Mutations	KIRC	367	TCGA	4.03252389847269
Mutations	KIRP	280	TCGA	3.07658499066037
Mutations	LAML	110	TCGA	1.208658389188
Mutations	LGG	507	TCGA	5.57081639380288
Mutations	LIHC	357	TCGA	3.92264586309197
Mutations	LUAD	511	TCGA	5.61476760795517
Mutations	LUSC	479	TCGA	5.26315789473684
Mutations	OV	286	TCGA	3.1425118118888
Mutations	PRAD	491	TCGA	5.39501153719371
Mutations	READ	144	TCGA	1.58224370948247
Mutations	SKCM	464	TCGA	5.09834084166575
Mutations	STAD	415	TCGA	4.55993846830019
Mutations	THCA	476	TCGA	5.23019448412262
Mutations	UCEC	528	TCGA	5.80156026810241
Mutations	UCS	57	TCGA	0.626304801670146
CNV	ACC	61	TCGA	0.670256015822437
CNV	BLCA	392	TCGA	4.30721898692451
CNV	BRCA	843	TCGA	9.26271838259532
CNV	CESC	268	TCGA	2.94473134820349
CNV	COAD	381	TCGA	4.18635314800571
CNV	DLBC	37	TCGA	0.406548730908691
CNV	GBM	136	TCGA	1.49434128117789
CNV	HNSC	472	TCGA	5.18624326997033
CNV	KICH	43	TCGA	0.472475552137128
CNV	KIRC	310	TCGA	3.40621909680255
CNV	KIRP	241	TCGA	2.64806065267553
CNV	LAML	42	TCGA	0.461487748599055
CNV	LGG	388	TCGA	4.26326777277222
CNV	LIHC	326	TCGA	3.58202395341171
CNV	LUAD	481	TCGA	5.28513350181299
CNV	LUSC	471	TCGA	5.17525546643226
CNV	OV	261	TCGA	2.86781672343698
CNV	PRAD	346	TCGA	3.80178002417317
CNV	READ	132	TCGA	1.4503900670256
CNV	SKCM	449	TCGA	4.93352378859466
CNV	STAD	393	TCGA	4.31820679046259
CNV	THCA	190	TCGA	2.08768267223382
CNV	UCEC	499	TCGA	5.4829139654983
CNV	UCS	54	TCGA	0.593341391055928
Viral counts	BLCA	247	TCGA	2.71398747390397
Viral counts	BRCA	816	TCGA	8.96604768706736
Viral counts	CESC	164	TCGA	1.80199978024393
Viral counts	COAD	358	TCGA	3.93363366663004
Viral counts	GBM	141	TCGA	1.54928029886826
Viral counts	HNSC	384	TCGA	4.21931655861993
Viral counts	KIRC	297	TCGA	3.2633776508076
Viral counts	KIRP	165	TCGA	1.812987583782

Viral counts	LGG	348	TCGA	3.82375563124931
Viral counts	LIHC	172	TCGA	1.88990220854851
Viral counts	LUAD	465	TCGA	5.10932864520382
Viral counts	LUSC	451	TCGA	4.95549939567081
Viral counts	OV	257	TCGA	2.82386550928469
Viral counts	PRAD	234	TCGA	2.57114602790902
Viral counts	READ	124	TCGA	1.36248763872102
Viral counts	SKCM	171	TCGA	1.87891440501044
Viral counts	STAD	267	TCGA	2.93374354466542
Viral counts	THCA	198	TCGA	2.1755851005384
Viral counts	UCEC	482	TCGA	5.29612130535106
Neoepitopes	BLCA	119	TCGA	1.30754862103066
Neoepitopes	BRCA	358	TCGA	3.93363366663004
Neoepitopes	CESC	142	TCGA	1.56026810240633
Neoepitopes	GBM	106	TCGA	1.16470717503571
Neoepitopes	HNSC	241	TCGA	2.64806065267553
Neoepitopes	KIRC	216	TCGA	2.37336556422371
Neoepitopes	KIRP	117	TCGA	1.28557301395451
Neoepitopes	LGG	120	TCGA	1.31853642456873
Neoepitopes	LIHC	145	TCGA	1.59323151302055
Neoepitopes	LUAD	138	TCGA	1.51631688825404
Neoepitopes	LUSC	143	TCGA	1.5712559059444
Neoepitopes	PRAD	147	TCGA	1.61520712009669
Neoepitopes	THCA	73	TCGA	0.80210965827931
Neoepitopes	UCEC	78	TCGA	0.857048675969674
MSKCC-IMPACT	BLCA	207	ICI	9.15524104378594
MSKCC-IMPACT	BRCA	34	ICI	1.50375939849624
MSKCC-IMPACT	CUP	76	ICI	3.36134453781513
MSKCC-IMPACT	COAD	108	ICI	4.77664750110571
MSKCC-IMPACT	ESCA	111	ICI	4.9093321539142
MSKCC-IMPACT	Glioma	111	ICI	4.9093321539142
MSKCC-IMPACT	HNSC	123	ICI	5.44007076514816
MSKCC-IMPACT	SKCM	287	ICI	12.6934984520124
MSKCC-IMPACT	NSCLC	304	ICI	13.4453781512605
MSKCC-IMPACT	KIRC	110	ICI	4.86510393631137
MSKCC-IMPACT	NMSC	1	ICI	0.044228217602831
Pender et al	LUSC	26	ICI	1.1499336576736
Pender et al	BRCA	13	ICI	0.574966828836798
Pender et al	SKCM	11	ICI	0.486510393631137
Pender et al	COAD	6	ICI	0.265369305616984
Pender et al	SARC	5	ICI	0.221141088014153
Pender et al	HNSC	5	ICI	0.221141088014153
Pender et al	CHOL	3	ICI	0.132684652808492
Pender et al	UCEC	3	ICI	0.132684652808492
Pender et al	AECA	2	ICI	0.088456435205661
Pender et al	KIRC	2	ICI	0.088456435205661
Pender et al	PAAD	2	ICI	0.088456435205661
Pender et al	STAD	2	ICI	0.088456435205661

Pender et al	UVM	9	ICI	0.398053958425475
Pender et al	ACC	2	ICI	0.088456435205661
Pender et al	ESCA	1	ICI	0.044228217602831
Pender et al	GCT	1	ICI	0.044228217602831
Pender et al	LYMP	1	ICI	0.044228217602831
Pender et al	OV	2	ICI	0.088456435205661
Pender et al	THYM	1	ICI	0.044228217602831
Pender et al	CESC	1	ICI	0.044228217602831
Miao et al	Anal Cancer	1	ICI	0.044228217602831
Miao et al	BLCA	27	ICI	1.19416187527643
Miao et al	HNSC	12	ICI	0.530738611233967
Miao et al	LUSC	57	ICI	2.52100840336134
Miao et al	SKCM	151	ICI	6.67846085802742
Miao et al	SARC	1	ICI	0.044228217602831
Braun et al	ccRCC	261	ICI	11.5435647943388
Liu et al	SKCM	144	ICI	6.36886333480761
Hugo et al	SKCM	37	ICI	1.63644405130473
Riaz et al	NIV3-NAIVE	33	Riaz et al	48.5294117647059
Riaz et al	NIV3-PROG	35	Riaz et al	51.4705882352941
Hematological	LAML	2103	Hematological	54.4959834153926
Hematological	DLBCL	944	Hematological	24.4622959315885
Hematological	Myelodysplastic Syndromes	685	Hematological	17.7507126198497
Hematological	Other Leukemias	127	Hematological	3.29100803316921
Pediatric	LAML	131	Pediatric	12.4643196955281
Pediatric	Acute Myeloid Leukemia	25	Pediatric	2.37868696479543
Pediatric	ACC	8	Pediatric	0.761179828734539
Pediatric	ATRT	17	Pediatric	1.61750713606089
Pediatric	B-ALL	40	Pediatric	3.80589914367269
Pediatric	Embryonal Tumor	10	Pediatric	0.951474785918173
Pediatric	Ependymoma	60	Pediatric	5.70884871550904
Pediatric	Ewing's sarcoma	24	Pediatric	2.28353948620362
Pediatric	HB	9	Pediatric	0.856327307326356
Pediatric	GBM	116	Pediatric	11.0371075166508
Pediatric	MB	190	Pediatric	18.0780209324453
Pediatric	NB	148	Pediatric	14.081826831589
Pediatric	NHL	15	Pediatric	1.42721217887726
Pediatric	OSG	23	Pediatric	2.1883920076118
Pediatric	CNS-PiloAstro	67	Pediatric	6.37488106565176
Pediatric	RB	27	Pediatric	2.56898192197907
Pediatric	RMS	9	Pediatric	0.856327307326356
Pediatric	T-ALL	1	Pediatric	0.095147478591817
Pediatric	Wilms Tumor	93	Pediatric	8.84871550903901
Pediatric	Other	38	Pediatric	3.61560418648906
Composite	ACC	56	Composite	0.178611297164546
Composite	AVC	55	Composite	0.175421809715179
Composite	Anal Cancer	75	Composite	0.239211558702517
Composite	Appendiceal Cancer	185	Composite	0.590055178132874
Composite	BLCA	949	Composite	3.02682358944918

Composite	OGS	252	Composite	0.803750837240455
Composite	BRCA	4822	Composite	15.3797084808471
Composite	Breast-AdenoCA	48	Composite	0.153095397569611
Composite	CUP	950	Composite	3.03001307689854
Composite	CESC	197	Composite	0.628329027525277
Composite	CNS	101	Composite	0.322138232386056
Composite	COAD	3012	Composite	9.60673619749306
Composite	UCEC	1261	Composite	4.02194367365164
Composite	ESCA	900	Composite	2.8705387044302
Composite	GEP-NET	109	Composite	0.347654131980991
Composite	GIST	408	Composite	1.30131087934169
Composite	GCT	383	Composite	1.22157369310752
Composite	Glioma	1476	Composite	4.70768347526552
Composite	HNSC	446	Composite	1.42251140241763
Composite	HCC	873	Composite	2.78442254329729
Composite	SKCM	1126	Composite	3.59136286798711
Composite	Mesothelioma	245	Composite	0.781424425094887
Composite	Nerve Sheath Tumor	58	Composite	0.184990272063279
Composite	NSCLC	4885	Composite	15.5806461901572
Composite	Other	259	Composite	0.826077249386024
Composite	OV	1036	Composite	3.30430899754409
Composite	PAAD	1782	Composite	5.68366663477179
Composite	PCS	159	Composite	0.507128504449335
Composite	PRAD	1906	Composite	6.07916307849329
Composite	KIRC	658	Composite	2.09868274168341
Composite	RB	41	Composite	0.130768985424042
Composite	SGC	239	Composite	0.762287500398686
Composite	Sellar	24	Composite	0.076547698784805
Composite	SCSTs	52	Composite	0.165853347367078
Composite	NMSC	247	Composite	0.787803399993621
Composite	SBA	114	Composite	0.363601569227825
Composite	SCLC	273	Composite	0.87073007367716
Composite	STS	910	Composite	2.90243357892387
Composite	THYM	40	Composite	0.127579497974675
Composite	THCA	547	Composite	1.74464963480369
Composite	UCS	194	Composite	0.618760565177176

Supplementary Table 2. Tumor types' abbreviations.

Tumor type	Abbreviation
Adrenocortical carcinoma	ACC
Acute yeloid leukemia	Acute Myeloid Leukemia
Apocrine eccrine carcinoma/sweat gland carcinoma	AECA
Anal cancer	Anal Cancer
Appendiceal cancer	Appendiceal Cancer
Atypical teratoid/rhabdoid tumor	ATRT
Ampullary cancer	AVC
B-Lymphoblastic leukemia/lymphoma	B-ALL
Bone-Benig	BBT

Bladder urothelial carcinoma	BLCA
Bone-Epith	Bone-Epith
Breast invasive carcinoma	BRCA
Breast adenocarcinoma	Breast-AdenoCA
Breast ductal carcinoma in situ	Breast-DCIS
Breast lobular carcinoma	Breast-LobularCA
Biliary adenocarcinoma	BTC
Clear cell renal cell carcinoma	ccRCC
Cervical squamous cell carcinoma and endocervical adenocarcinoma	CESC
Cholangiocarcinoma	CHOL
Central nervous system cancer	CNS
CNS medulloblastoma	CNS-Medullo
CNS oligodendroglioma	CNS-Oligo
CNS pilocytic astrocytoma	CNS-PiloAstro
Colon adenocarcinoma	COAD
Cancer of unknown primary	CUP
Diffuse large B-cell lymphoma	DLBCL
EMbryonal tumor	Embryonal Tumor
Ependymoma	Ependymoma
Esophageal carcinoma	ESCA
Ewing's sarcoma	Ewing's sarcoma
Glioblastoma multiforme	GBM
Germ cell tumor	GCT
Gastrointestinal neuroendocrine Tumor	GEP-NET
Gastrointestinal stromal tumor	GIST
Glioma	Glioma
Hepatoblastoma	HB
Hepatobiliary cancer	HCC
Head and neck squamous cell carcinoma	HNSC
Kidney Chromophobe	KICH
Kidney renal clear cell carcinoma	KIRC
Kidney renal papillary cell carcinoma	KIRP
Acute myeloid leukemia	LAML
Low grade glioma	LGG
Liver hepatocellular carcinoma	LIHC
Lung adenocarcinoma	LUAD
Lung squamous cell carcinoma	LUSC
Lymphoma	LYMP
Non-Hodgkin lymphoma	Lymph-BNHL
Chronic lymphocytic leukemia	Lymph-CLL
Medulloblastoma	MB
Mesothelioma	Mesothelioma
Myelodysplastic syndromes	Myelodysplastic Syndromes
Myeloid myelodysplasia	Myeloid-MDS
Myeloid myeloproliferative neoplasm	Myeloid-MPN
Neuroblastoma	NB
Nerve sheath tumor	Nerve Sheath Tumor
Melanoma Nivolumab treated	NIV3-NAIVE

Melanoma Nivolumab/Ipilimumab treated	NIV3-PROG
Non-melanoma skin cancer	NMSC
Non-Small cell lung cancer	NSCLC
Bone osteosarcoma	OGS/OSG
Other	Other
Other leukemias	Other Leukemias
Ovarian serous cystadenocarcinoma	OV
Ovary adenocarcinoma	Ovary-AdenoCA
Pancreatic adenocarcinoma	PAAD
Pancreatic endocrine	Panc-Endocrine
Peripheral nervous system	PCS
Prostate adenocarcinoma	PRAD
Retinoblastoma	RB
Rectum adenocarcinoma	READ
Rhabdomyosarcoma	RMS
Sarcoma	SARC
Small bowel cancer	SBA
Small cell lung cancer	SCLC
Sex cord stromal tumor	SCSTs
Sellar cancer	Sellar
Salivary gland cancer	SGC
Skin Cutaneous Melanoma	SKCM
Stomach adenocarcinoma	STAD
Soft-tissue liposarcoma	STS
T-Lymphoblastic Leukemia/Lymphoma	T-ALL
Thyroid carcinoma	THCA
Thymoma	THYM
Uterine Corpus Endometrial Carcinoma	UCEC
Uterine Carcinosarcoma	UCS
Uveal Melanoma	UVM
Wilms tumor	Wilms Tumor

Supplementary Table 3. R-loops statistics. Number of mutations falling “in” or “out” R-loops that are associated to different mutational processes. Last two columns represent the mutations using simulated mutational profiles (see methods). Fisher test is always set to compare c-AID mutations (second column) versus any other column.

**Rloops**	**AID_canonical (c-AID)**	**SBS13**	**SBS2**	**Other_than_AID**	**AID_canonical in simulated data**	**Other_than_AID in simulated data**
out_Rloops	628741	204522	241239	13434280	233838	13828460
in_Rloops	1130	400	456	22648	315	24188
Fisher.test_AID_vs_	1	0.153	0.369	0.037	**3.74E-06**	0.371

Supplementary Table 4. Differential expressed genes (p-adj <0.20) between high ICN

load patients versus low ICN load for pre-therapy. This is related to Fig. 4d.

baseMean	log2FoldChange	lfcSE	stat	pvalue	padj	weight	symbol
36.7228	-2.3373	0.4610	-5.0706	0.0000	0.0022	1.0965	CREB1
420.6771	5.8530	1.2639	4.6309	0.0000	0.0060	1.8332	DCTN2
482.9950	-5.9236	1.2754	-4.6446	0.0000	0.0068	1.0000	IL12RB1
80.6830	-4.0539	0.9181	-4.4157	0.0000	0.0087	1.7437	KCNN2
481.5198	6.0016	1.3130	4.5711	0.0000	0.0102	0.5718	FPR3
88.6086	5.3046	1.2538	4.2308	0.0000	0.0129	1.8087	PTPRR
8.2360	5.3607	1.2433	4.3117	0.0000	0.0139	1.0000	HK1
65.3985	-2.6995	0.6878	-3.9247	0.0001	0.0244	1.8087	MBNL2
276.2939	3.7743	0.9579	3.9401	0.0001	0.0244	1.7437	GPR37
4.5701	4.8447	1.1666	4.1528	0.0000	0.0244	1.0000	MCAM
164.6372	5.2388	1.3294	3.9406	0.0001	0.0244	1.8332	PPEF2
358.2882	3.1398	0.7765	4.0434	0.0001	0.0244	1.0000	PGLYRP1
421.9155	-1.0836	0.2774	-3.9057	0.0001	0.0244	1.8087	PITPNM1
130.7295	2.4003	0.6226	3.8553	0.0001	0.0266	1.7437	HSF1
422.8403	-2.8627	0.7426	-3.8548	0.0001	0.0266	1.8332	TRAF2
117.0324	-1.9805	0.5181	-3.8224	0.0001	0.0271	1.8332	SNRPD2
93.4333	5.4009	1.4600	3.6992	0.0002	0.0374	1.8332	RASGRP2
2340.3790	-0.7720	0.1961	-3.9376	0.0001	0.0374	0.7159	FNTA
118.3659	-2.1928	0.5916	-3.7064	0.0002	0.0374	1.8087	ZNF80
203.5373	-1.0931	0.2981	-3.6667	0.0002	0.0400	1.8087	MLANA
385.5912	-0.6719	0.1839	-3.6527	0.0003	0.0400	1.8087	NDUFB3
22.4635	-4.6118	1.1944	-3.8612	0.0001	0.0400	0.7716	EIPR1
57.2168	-2.4991	0.6889	-3.6277	0.0003	0.0414	1.8087	DDO
21.4327	4.1438	1.0948	3.7850	0.0002	0.0480	0.7716	CSTF2
752.1751	3.4681	0.8970	3.8665	0.0001	0.0480	0.5700	PIN1P1
523.6555	3.1172	0.8432	3.6970	0.0002	0.0484	1.0000	CLEC3A
210.5894	-3.4456	0.9360	-3.6813	0.0002	0.0484	1.0000	NISCH
17.1012	-3.6884	1.0012	-3.6840	0.0002	0.0484	1.0965	PSMA1
284.6012	-1.3734	0.3732	-3.6803	0.0002	0.0484	1.0000	CLINT1
152.7975	-1.6789	0.4818	-3.4849	0.0005	0.0505	1.7437	TOMM34
52.9802	-2.7765	0.7625	-3.6412	0.0003	0.0505	1.0000	PTCH1
1510.9496	-0.6621	0.1768	-3.7444	0.0002	0.0505	0.7159	SH3GL1P1
2739.7126	-2.1634	0.5962	-3.6284	0.0003	0.0505	1.0000	VCAM1
297.0295	-4.2756	1.1707	-3.6521	0.0003	0.0505	1.0000	CDK1
31.6913	5.4860	1.5241	3.5996	0.0003	0.0519	1.0000	HRH3
50.5588	-1.9805	0.5527	-3.5833	0.0003	0.0519	1.0965	SCN9A
1472.6938	-0.6793	0.1814	-3.7443	0.0002	0.0519	0.5718	WNT10B
167.4052	2.9676	0.8684	3.4175	0.0006	0.0546	1.8332	TPST1
511.8743	3.5698	0.9994	3.5721	0.0004	0.0547	1.0000	B4GALT1
73.7943	-2.0140	0.5936	-3.3931	0.0007	0.0567	1.8332	EPHB4
151.4727	-0.8666	0.2463	-3.5184	0.0004	0.0637	1.0000	HOXC5
170.9531	-2.6194	0.7809	-3.3541	0.0008	0.0654	1.7437	NDUFS6
78.0090	-2.4818	0.7425	-3.3425	0.0008	0.0667	1.7437	PDC
22.1980	-1.9480	0.5626	-3.4622	0.0005	0.0668	1.0965	ARSL
2272.7494	2.2160	0.6159	3.5982	0.0003	0.0685	0.5700	CDK2AP2
158.9476	-2.0898	0.6404	-3.2631	0.0011	0.0685	1.8087	APC2

3927.0740	-1.4897	0.4327	-3.4431	0.0006	0.0685	1.0000	FANCE
298.6623	-2.6805	0.8188	-3.2735	0.0011	0.0685	1.7437	GRINA
28.4483	4.5807	1.3407	3.4166	0.0006	0.0685	1.0000	ITIH2
94.9018	-1.5555	0.4775	-3.2576	0.0011	0.0685	1.8087	KCNA4
85.2015	4.0690	1.2451	3.2680	0.0011	0.0685	1.8332	NOTCH3
47.7616	-0.9840	0.2903	-3.3903	0.0007	0.0685	1.0965	PRKAB1
735.1542	-0.6418	0.1864	-3.4425	0.0006	0.0685	1.0000	RGS10
66.4478	2.9222	0.8853	3.3006	0.0010	0.0685	1.8332	SYCP1
356.8471	-0.9137	0.2665	-3.4283	0.0006	0.0685	1.0000	STX10
1678.0185	3.9042	1.1345	3.4415	0.0006	0.0685	1.0000	GOLGA5
89.1853	-2.9099	0.9046	-3.2167	0.0013	0.0707	1.8087	CFL1
104.2978	-1.9023	0.5915	-3.2158	0.0013	0.0707	1.8332	MYD88
1691.7720	1.9254	0.5456	3.5293	0.0004	0.0707	0.5718	EXOSC9
957.3389	2.5421	0.7189	3.5362	0.0004	0.0707	0.5700	MAP3K12
142.3874	-1.3731	0.4267	-3.2183	0.0013	0.0707	1.7437	CDC16
3436.0565	1.4661	0.4344	3.3751	0.0007	0.0707	1.0000	NEMF
227.0175	6.1361	1.9071	3.2175	0.0013	0.0707	1.8087	LITAF
477.8773	-2.5258	0.7190	-3.5128	0.0004	0.0729	0.5718	NUDT4
3790.7348	-0.8672	0.2472	-3.5074	0.0005	0.0733	0.5718	CNGA2
52.4339	-4.8691	1.4580	-3.3396	0.0008	0.0763	1.0000	ADAMTS7
46.1120	-1.3013	0.3819	-3.4077	0.0007	0.0763	0.7716	APOA4
674.1096	-0.9243	0.2655	-3.4809	0.0005	0.0774	0.5718	HSPA1B
11.1969	2.1840	0.6595	3.3116	0.0009	0.0809	1.0000	LYST
22.9026	-1.1914	0.3605	-3.3052	0.0009	0.0816	1.0000	KIF1A
121.0604	-2.1766	0.7009	-3.1056	0.0019	0.0890	1.8087	PGM5
20.0148	-1.5044	0.4614	-3.2607	0.0011	0.0917	1.0000	YME1L1
765.2115	1.2945	0.3966	3.2640	0.0011	0.0917	1.0000	ETFA
6265.2011	2.0758	0.6221	3.3366	0.0008	0.0964	0.7159	RGS7
40.8744	-3.7250	1.1521	-3.2331	0.0012	0.0977	1.0000	CFDP1
102.9902	-1.1641	0.3603	-3.2311	0.0012	0.0977	1.0000	SPACA9
248.4917	2.1025	0.6902	3.0461	0.0023	0.0989	1.8332	VAPB
10379.8058	0.5924	0.1843	3.2145	0.0013	0.1008	1.0000	DLX3
1959.4491	-1.8708	0.5832	-3.2078	0.0013	0.1019	1.0000	OCM2
30.8707	3.7665	1.1807	3.1901	0.0014	0.1024	1.0456	PFAS
121.6834	3.5043	1.1627	3.0140	0.0026	0.1045	1.8332	SRP68
6603.2948	3.2253	0.9827	3.2820	0.0010	0.1054	0.7159	PMS2P1
241.0947	-1.0905	0.3625	-3.0081	0.0026	0.1054	1.8087	H4C3
155.3335	-1.4150	0.4466	-3.1683	0.0015	0.1099	1.0000	MEGF8
503.1074	-2.3346	0.7402	-3.1539	0.0016	0.1141	1.0000	EOMES
274.9179	-0.7669	0.2593	-2.9578	0.0031	0.1183	1.8332	ADGRE5
91.9687	-1.8322	0.6172	-2.9687	0.0030	0.1187	1.7437	MYBPC1
224.0363	-1.2837	0.4124	-3.1126	0.0019	0.1241	1.0000	SET
46.3734	-2.3448	0.7351	-3.1897	0.0014	0.1241	0.7716	ST3GAL3
137.1359	-1.1179	0.3803	-2.9397	0.0033	0.1241	1.8087	BFSP2
771.0329	-0.9702	0.2969	-3.2680	0.0011	0.1257	0.5700	HOXB5
619.6234	2.3160	0.7099	3.2627	0.0011	0.1258	0.5718	SDS
344.3404	-0.8662	0.2974	-2.9121	0.0036	0.1258	1.8087	CELF3
64.9796	-2.3705	0.7652	-3.0979	0.0019	0.1258	1.0000	SULT2B1

96.0177	-2.9211	0.9441	-3.0942	0.0020	0.1258	1.0000	CYTH3
5543.8400	-0.8892	0.2891	-3.0757	0.0021	0.1290	1.0000	TPPP
471.5271	-2.2436	0.7068	-3.1741	0.0015	0.1290	0.7159	IL5RA
5.1259	3.9774	1.2604	3.1556	0.0016	0.1290	0.7716	FIG4
921.6653	2.6120	0.8513	3.0682	0.0022	0.1296	1.0000	IL1R1
24.2830	-1.5672	0.5153	-3.0411	0.0024	0.1296	1.0965	CEP57
95.5343	-1.3874	0.4534	-3.0602	0.0022	0.1313	1.0000	PDCD7
104.2487	-1.2260	0.4009	-3.0585	0.0022	0.1313	1.0000	FHL3
5021.7531	-0.6040	0.1977	-3.0553	0.0022	0.1314	1.0000	DRD5
297.8214	-0.5717	0.1874	-3.0511	0.0023	0.1320	1.0000	JAKMIP2
64.9984	2.6374	0.8658	3.0463	0.0023	0.1328	1.0000	MYB
220.5021	3.8746	1.3543	2.8610	0.0042	0.1375	1.7437	HCLS1
130.5915	-0.9759	0.3230	-3.0211	0.0025	0.1378	1.0000	DEFA1
1273.3658	0.4423	0.1386	3.1913	0.0014	0.1378	0.5718	EPHA4
5497.1296	1.5892	0.4986	3.1871	0.0014	0.1378	0.5718	PMM2
147.6496	-2.3232	0.8136	-2.8554	0.0043	0.1378	1.7437	TNNC1
7.8371	-3.7111	1.2422	-2.9875	0.0028	0.1391	1.0965	NKX2-5
82.7629	-1.8763	0.6253	-3.0004	0.0027	0.1436	1.0000	CST6
4672.6709	-0.7802	0.2600	-3.0010	0.0027	0.1436	1.0000	GTF2H1
31.6918	1.7904	0.6041	2.9638	0.0030	0.1470	1.0456	PRSS23
130.4796	1.9358	0.6913	2.8001	0.0051	0.1470	1.7437	PAWR
509.3338	0.7290	0.2316	3.1483	0.0016	0.1470	0.5700	ST14
374.3275	-0.8808	0.2955	-2.9811	0.0029	0.1470	1.0000	H4C9
43.7704	1.4478	0.4727	3.0630	0.0022	0.1470	0.7716	NOL3
8.9311	-3.5841	1.1693	-3.0651	0.0022	0.1470	0.7716	NPEPPS
3610.3551	-3.4268	1.1111	-3.0842	0.0020	0.1470	0.7159	RAB11FIP3
1121.4811	-3.0948	1.0434	-2.9662	0.0030	0.1478	1.0000	WFDC2
3805.7786	-0.4580	0.1545	-2.9642	0.0030	0.1478	1.0000	DYNC1I2
174.6715	-1.9067	0.6871	-2.7750	0.0055	0.1478	1.8332	PFKFB4
193.0899	-0.8137	0.2919	-2.7877	0.0053	0.1478	1.7437	IL1R2
142.4311	-0.8846	0.2990	-2.9586	0.0031	0.1488	1.0000	CD19
118.6457	-0.5722	0.2070	-2.7637	0.0057	0.1510	1.8087	NR2E1
49.4682	-0.9760	0.3313	-2.9463	0.0032	0.1525	1.0000	USH1C
415.9744	-0.4224	0.1536	-2.7498	0.0060	0.1551	1.8087	UQCRC1
120.9671	-1.2693	0.4614	-2.7511	0.0059	0.1589	1.7437	UCK2
144.0997	0.9928	0.3396	2.9237	0.0035	0.1602	1.0000	IFITM1
226.7116	2.2928	0.8365	2.7408	0.0061	0.1603	1.7437	PIK3R3
1717.1747	-1.0865	0.3514	-3.0915	0.0020	0.1603	0.5700	NUP155
107.1163	0.9072	0.3134	2.8948	0.0038	0.1693	1.0000	HBM
15.4122	-1.2050	0.4162	-2.8951	0.0038	0.1693	1.0000	HLA-DRB1
947.0503	3.6232	1.1815	3.0667	0.0022	0.1693	0.5700	ZBTB17
2187.9013	-0.6387	0.2086	-3.0620	0.0022	0.1695	0.5700	KLF8
145.2630	3.1937	1.1900	2.6838	0.0073	0.1695	1.8332	MOCS2
14.2645	-1.8391	0.6413	-2.8677	0.0041	0.1695	1.0456	TSHR
179.5194	2.8046	0.9720	2.8854	0.0039	0.1695	1.0000	TSNAX
115.5863	2.2714	0.7873	2.8852	0.0039	0.1695	1.0000	WNT9B
57.7633	-0.7546	0.2620	-2.8806	0.0040	0.1695	1.0000	NCOA1
28.6764	4.5227	1.5737	2.8739	0.0041	0.1719	1.0000	MRC1

12.0796	-1.5197	0.5307	-2.8636	0.0042	0.1763	1.0000	APOC1P1
259.3917	0.7321	0.2738	2.6735	0.0075	0.1800	1.7437	SCML2
75.4623	-0.6163	0.2324	-2.6521	0.0080	0.1812	1.8332	NAT1
270.2177	-0.8445	0.3193	-2.6450	0.0082	0.1813	1.8332	CITED2
3.8597	-3.0963	1.0995	-2.8161	0.0049	0.1813	1.0965	PLXNA2
1880.6731	-0.9926	0.3287	-3.0198	0.0025	0.1813	0.5718	KMT2D
83.6933	2.4952	0.8796	2.8369	0.0046	0.1814	1.0000	ATP5PD
4650.8762	0.5914	0.2009	2.9440	0.0032	0.1814	0.7159	EZH1
635.9725	-0.8340	0.2940	-2.8364	0.0046	0.1814	1.0000	FYN
2457.2007	-0.6877	0.2426	-2.8352	0.0046	0.1814	1.0000	NUMB
167.4014	-2.0577	0.7268	-2.8314	0.0046	0.1824	1.0000	CD8B
398.8994	-0.4190	0.1598	-2.6218	0.0087	0.1890	1.8087	PSG1
33.2880	-1.4008	0.5038	-2.7807	0.0054	0.1918	1.0965	CHAT
15.7578	-2.3794	0.8477	-2.8069	0.0050	0.1918	1.0000	VAMP7
174.6139	-0.8384	0.3196	-2.6230	0.0087	0.1918	1.7437	COIL
102.3333	2.6298	1.0117	2.5995	0.0093	0.1967	1.8087	DNM1
1070.8120	1.8376	0.6239	2.9455	0.0032	0.1970	0.5718	SGK2
597.4451	-2.0969	0.7273	-2.8831	0.0039	0.1970	0.7159	BCAS2
10498.1180	1.3664	0.4611	2.9632	0.0030	0.1970	0.5700	RNF13
216.4094	2.0478	0.7350	2.7862	0.0053	0.1970	1.0000	DSPP
159.5228	4.7393	1.8381	2.5784	0.0099	0.1970	1.8332	GTF2H2
147.4735	2.2295	0.8617	2.5875	0.0097	0.1970	1.7437	H3-3A
49.5918	-3.1992	1.1604	-2.7570	0.0058	0.1970	1.0965	APBA2
348.7440	-0.4270	0.1656	-2.5783	0.0099	0.1970	1.7437	IRF8
81.5337	-1.4403	0.5585	-2.5786	0.0099	0.1970	1.8087	RPL10A
30.9458	2.3826	0.8609	2.7676	0.0056	0.1970	1.0000	PCBP2
148.9859	2.1078	0.7555	2.7898	0.0053	0.1970	1.0000	RGS2
48.2991	-1.2183	0.4364	-2.7919	0.0052	0.1970	1.0000	BYSL
5.4983	-2.6936	0.9435	-2.8550	0.0043	0.1970	0.7716	USP1
123.4034	1.6913	0.6554	2.5805	0.0099	0.1970	1.8332	PLPP3
224.5562	-0.5078	0.1979	-2.5665	0.0103	0.1970	1.8087	EIF4EBP3
100.1103	-1.1488	0.4475	-2.5670	0.0103	0.1970	1.8332	CCNK

Supplementary Table 5. Differential expressed genes (p-adj <0.20) by comparing pre-therapy to on-therapy data from the patients (n_{pre} = 20; n_{on} = 20). This is related to Fig. 4d.

baseMean	log2FoldChange	lfcSE	stat	pvalue	padj	symbol
9.74641	22.07555	1.92547	11.46500	0.00000	0.00000	PRKCQ
10.86542	22.40440	2.73683	8.18626	0.00000	0.00000	STK24
16.11131	23.06569	2.97443	7.75467	0.00000	0.00000	CXCL13
628.13627	-7.21865	0.97454	-7.40728	0.00000	0.00000	IL12RB1
7.32444	21.64794	2.97643	7.27313	0.00000	0.00000	ELK3
120.47592	6.20423	0.87096	7.12342	0.00000	0.00000	PDK1
453.61667	5.97469	0.97350	6.13730	0.00000	0.00000	FPR3
739.80026	4.99032	0.81819	6.09920	0.00000	0.00000	GAST
278.11015	4.86687	0.82491	5.89986	0.00000	0.00000	KAT2B
98.91328	-3.33216	0.57100	-5.83565	0.00000	0.00000	MBNL2

84.34816	6.78570	1.16238	5.83778	0.00000	0.00000	GLP2R
255.58558	6.04607	1.04077	5.80925	0.00000	0.00000	CDK18
2264.29234	-2.27920	0.39497	-5.77054	0.00000	0.00000	VCAM1
199.72449	-3.37067	0.60527	-5.56884	0.00000	0.00001	NISCH
194.53082	-4.37217	0.78783	-5.54966	0.00000	0.00001	CDK1
73.50207	5.09528	0.92296	5.52058	0.00000	0.00001	DOCK2
135.68470	4.40340	0.80575	5.46494	0.00000	0.00002	SLC9A6
441.57070	-2.77345	0.50672	-5.47334	0.00000	0.00002	IL5RA
318.21871	-2.73718	0.50466	-5.42383	0.00000	0.00002	TRAF2
1085.13161	4.05802	0.75635	5.36529	0.00000	0.00002	CBX3
384.27012	4.02158	0.74942	5.36628	0.00000	0.00002	XPR1
1769.63132	4.58859	0.85874	5.34341	0.00000	0.00003	GOLGA5
359.77282	3.19248	0.60491	5.27761	0.00000	0.00004	PLAGL2
220.31921	2.63454	0.50315	5.23614	0.00000	0.00004	CLDN12
59.58986	-2.84280	0.54592	-5.20736	0.00000	0.00005	SULT2B1
156.70002	4.39640	0.86431	5.08660	0.00000	0.00009	GHRH
439.31214	3.51155	0.69374	5.06179	0.00000	0.00010	TRIM13
146.20906	5.26495	1.06031	4.96550	0.00000	0.00016	PPEF2
83.43552	6.42633	1.30289	4.93235	0.00000	0.00017	GEM
55.21806	-2.22831	0.45160	-4.93429	0.00000	0.00017	DDO
982.44378	3.97989	0.81304	4.89505	0.00000	0.00020	ZBTB17
2150.26359	1.82974	0.37442	4.88691	0.00000	0.00020	CDK2AP2
159.53656	4.64975	0.95463	4.87073	0.00000	0.00021	IFI44L
213.06116	-1.39214	0.28749	-4.84242	0.00000	0.00024	SET
1679.42600	3.86675	0.80465	4.80549	0.00000	0.00028	MED23
108.47889	9.50937	1.98609	4.78797	0.00000	0.00030	FUT5
195.87849	2.57865	0.54371	4.74273	0.00000	0.00036	TRA2B
431.61541	-2.64787	0.56077	-4.72188	0.00000	0.00039	NUDT4
600.29288	2.43933	0.53605	4.55060	0.00001	0.00087	ROCK2
1805.75588	-0.37564	0.08311	-4.51989	0.00001	0.00096	SNRPG
208.58445	2.37375	0.52502	4.52127	0.00001	0.00096	WNT9B
128.24402	3.07036	0.68151	4.50525	0.00001	0.00099	MYB
154.02964	2.58623	0.57432	4.50310	0.00001	0.00099	TPST1
1123.14349	2.36824	0.53021	4.46659	0.00001	0.00110	TP53TG1
85.59154	-1.87863	0.42018	-4.47105	0.00001	0.00110	EPHB4
16.54319	-7.93011	1.77429	-4.46946	0.00001	0.00110	H3C2
104.10472	-2.06661	0.46391	-4.45473	0.00001	0.00114	PGM5
2268.80359	-0.79201	0.17868	-4.43262	0.00001	0.00123	DIAPH2
893.24446	2.48114	0.56151	4.41868	0.00001	0.00129	SDS
28.43646	-1.80406	0.40917	-4.40913	0.00001	0.00130	CREB1
74.09830	-1.61856	0.36716	-4.40834	0.00001	0.00130	MYBPC1
454.82896	-2.63128	0.59827	-4.39815	0.00001	0.00134	TOMM34
638.16977	5.07206	1.15466	4.39267	0.00001	0.00134	RPS2
148.84199	3.49553	0.80165	4.36042	0.00001	0.00153	RAD23B
372.72138	-0.54972	0.12651	-4.34529	0.00001	0.00161	NDUFB3
12.58714	-3.35073	0.77646	-4.31537	0.00002	0.00178	KLF1
12.94376	-5.92779	1.37475	-4.31191	0.00002	0.00178	FGF9
93.46040	4.05507	0.94031	4.31248	0.00002	0.00178	GOT2

157.27000	-2.59378	0.60326	-4.29961	0.00002	0.00184	NDUFS6
2548.07111	-1.92815	0.44957	-4.28889	0.00002	0.00190	TYMS
569.89610	0.77441	0.18080	4.28329	0.00002	0.00192	ST14
831.97482	2.61606	0.61443	4.25769	0.00002	0.00212	CLEC3A
482.97880	3.01961	0.71210	4.24044	0.00002	0.00222	PDIA5
1335.57765	-0.67365	0.15878	-4.24265	0.00002	0.00222	NEK2
1205.83135	3.14668	0.74455	4.22632	0.00002	0.00229	B4GALT1
237.82210	2.11416	0.50028	4.22592	0.00002	0.00229	VAPB
12.32957	6.33440	1.51565	4.17933	0.00003	0.00275	CELSR2
688.53915	-2.02156	0.48383	-4.17821	0.00003	0.00275	H1-4
1440.30661	1.45328	0.34955	4.15751	0.00003	0.00297	EXOSC9
173.51406	4.67087	1.12607	4.14795	0.00003	0.00305	RPL8
56.61316	-2.39799	0.58283	-4.11441	0.00004	0.00344	PDC
284.24811	3.03519	0.73777	4.11403	0.00004	0.00344	TSNAX
1469.05630	-0.59521	0.14532	-4.09597	0.00004	0.00366	SH3GL1P1
117.27404	2.06353	0.50511	4.08531	0.00004	0.00378	BPNT1
38.24484	4.99851	1.23225	4.05640	0.00005	0.00423	RASGRP2
42.04090	4.18011	1.04118	4.01478	0.00006	0.00492	NID1
19.23551	-4.05187	1.00870	-4.01694	0.00006	0.00492	CD5L
1684.28451	-0.86334	0.21527	-4.01042	0.00006	0.00493	KMT2D
228.73838	3.41065	0.85094	4.00807	0.00006	0.00493	ITM2B
19.60623	4.69613	1.17979	3.98048	0.00007	0.00547	HNRNPF
85.59796	-1.16614	0.29519	-3.95044	0.00008	0.00591	PDCD7
22.59826	5.61931	1.42004	3.95714	0.00008	0.00591	FOSB
41.86834	4.71205	1.19242	3.95167	0.00008	0.00591	SLC25A4
8.32549	4.06727	1.02900	3.95263	0.00008	0.00591	HOMER3
13.22069	-3.48605	0.88636	-3.93299	0.00008	0.00628	EIPR1
646.64018	1.03633	0.26476	3.91426	0.00009	0.00656	ETFA
9.97721	-5.66354	1.44540	-3.91831	0.00009	0.00656	NRTN
225.32238	1.99866	0.51046	3.91539	0.00009	0.00656	PIK3R3
142.92380	3.18727	0.81608	3.90558	0.00009	0.00672	PNRC1
477.67865	2.77929	0.71234	3.90162	0.00010	0.00675	ZNF460
63.27996	2.36874	0.60849	3.89282	0.00010	0.00693	NDUFV2
34.72804	2.64646	0.68065	3.88815	0.00010	0.00699	PTTG1
43.06827	2.30445	0.59486	3.87393	0.00011	0.00728	PAK2
401.75741	-2.36317	0.61019	-3.87284	0.00011	0.00728	SMC3
1265.48613	0.38331	0.09925	3.86217	0.00011	0.00745	EPHA4
51.19954	-2.19072	0.56715	-3.86265	0.00011	0.00745	PTCH1
6914.30300	1.29466	0.33589	3.85443	0.00012	0.00761	PSME3
553.35642	0.86938	0.22603	3.84628	0.00012	0.00779	KDM4A
454.01751	-1.24014	0.32285	-3.84119	0.00012	0.00787	KHDRBS1
128.78123	5.57152	1.45615	3.82620	0.00013	0.00828	TPD52L1
143.33167	2.65472	0.69487	3.82045	0.00013	0.00839	MX2
1346.13284	-0.87496	0.22979	-3.80762	0.00014	0.00875	SNCB
164.19668	-2.10617	0.55630	-3.78602	0.00015	0.00936	TNNC1
108.39764	-1.26301	0.33351	-3.78707	0.00015	0.00936	UCK2
75.71511	2.62154	0.69366	3.77929	0.00016	0.00953	TRIM38
61.34618	2.41522	0.63957	3.77631	0.00016	0.00955	MLEC

3165.00883	0.87447	0.23253	3.76073	0.00017	0.01007	SNRPB2
14.10426	-3.08121	0.82281	-3.74475	0.00018	0.01064	GSTM1
22.62069	3.29752	0.88634	3.72039	0.00020	0.01161	NNAT
214.35092	2.49479	0.67225	3.71109	0.00021	0.01194	NIT1
84.45950	2.07709	0.56035	3.70676	0.00021	0.01203	ATP5PD
61.74029	2.63657	0.71504	3.68731	0.00023	0.01281	PRKAR1A
830.13694	-0.47846	0.12979	-3.68629	0.00023	0.01281	SNCAIP
501.92119	2.30956	0.62863	3.67395	0.00024	0.01326	DYNC1LI2
1316.56566	-0.47515	0.12936	-3.67309	0.00024	0.01326	TBXT
222.60473	3.31606	0.90352	3.67017	0.00024	0.01326	DCTN2
48.26840	6.85768	1.86932	3.66854	0.00024	0.01326	OSBPL2
55.00228	4.46207	1.21850	3.66194	0.00025	0.01350	CTSF
42.05283	2.22998	0.60985	3.65658	0.00026	0.01367	IFNAR2
2030.04940	-0.58867	0.16148	-3.64546	0.00027	0.01415	POLR2F
20.95296	3.16313	0.86979	3.63667	0.00028	0.01452	PTPRR
1032.93346	-1.14219	0.31549	-3.62037	0.00029	0.01534	KLKB1
73.62208	2.20447	0.61173	3.60368	0.00031	0.01623	L1CAM
98.55774	-2.09323	0.58125	-3.60126	0.00032	0.01625	MYD88
224.30539	3.12783	0.87016	3.59454	0.00032	0.01654	PSMA4
122.87461	3.99514	1.11233	3.59169	0.00033	0.01659	TMEM59
25.69646	1.98804	0.55531	3.58008	0.00034	0.01721	LTBP3
109.39306	1.72835	0.48490	3.56434	0.00036	0.01799	HSF1
285.68700	1.10364	0.30956	3.56517	0.00036	0.01799	PKN2
375.20495	1.38500	0.38990	3.55219	0.00038	0.01855	HAS2
2.32791	3.15989	0.88939	3.55286	0.00038	0.01855	ASGR2
50.30793	3.08185	0.86920	3.54562	0.00039	0.01888	PSMD11
13.16534	4.24007	1.19890	3.53662	0.00041	0.01939	LTA4H
53.08553	2.07295	0.58747	3.52863	0.00042	0.01954	NCK1
1798.64719	-1.68135	0.47603	-3.53206	0.00041	0.01954	OCM2
569.42463	-2.07024	0.58655	-3.52951	0.00042	0.01954	EOMES
879.77199	-0.95522	0.27200	-3.51185	0.00044	0.02066	KIF22
7.94995	-4.15306	1.18675	-3.49951	0.00047	0.02143	CSF2RB
96.35035	1.89901	0.54283	3.49837	0.00047	0.02143	AP3B1
421.30417	-1.11780	0.32156	-3.47618	0.00051	0.02311	SUCLA2
95.27129	5.97337	1.72522	3.46239	0.00054	0.02349	SPRY2
48.83029	3.76354	1.08611	3.46517	0.00053	0.02349	KMT2A
3.73222	-2.26870	0.65463	-3.46563	0.00053	0.02349	PLXNA2
17.62570	2.48770	0.71725	3.46842	0.00052	0.02349	UMOD
5.73044	2.89197	0.83507	3.46315	0.00053	0.02349	FIG4
32.53050	-2.28568	0.66090	-3.45844	0.00054	0.02359	PFN2
143.89835	2.64076	0.76376	3.45757	0.00055	0.02359	S100A12
3693.62587	-2.93073	0.84849	-3.45406	0.00055	0.02374	RAB11FIP3
589.05147	-0.66013	0.19164	-3.44466	0.00057	0.02441	FYN
59.07973	2.93632	0.85469	3.43551	0.00059	0.02508	DCAF7
347.31464	0.78435	0.22857	3.43154	0.00060	0.02529	SCML2
868.51209	1.03657	0.30255	3.42612	0.00061	0.02551	PLPBP
1160.50879	1.42894	0.41714	3.42556	0.00061	0.02551	KCNS3
124.04385	-0.71419	0.20870	-3.42207	0.00062	0.02567	DEFA1

5313.59944	-0.83108	0.24390	-3.40747	0.00066	0.02640	ZNF12
175.58833	-1.73971	0.51044	-3.40825	0.00065	0.02640	RND2
61.81351	-0.89027	0.26096	-3.41148	0.00065	0.02640	ZNF239
486.94833	-1.24220	0.36429	-3.40994	0.00065	0.02640	CD40LG
5239.20783	0.74998	0.22027	3.40485	0.00066	0.02649	RASSF9
134.15488	-1.63020	0.47980	-3.39767	0.00068	0.02702	CD8B
8384.35341	-0.69631	0.20548	-3.38876	0.00070	0.02774	ENDOU
1276.71489	1.05932	0.31299	3.38450	0.00071	0.02783	RPS5
3900.37222	1.88239	0.55612	3.38486	0.00071	0.02783	GIT2
1961.42499	0.49093	0.14529	3.37899	0.00073	0.02822	H4C14
6261.77888	-2.12637	0.62987	-3.37589	0.00074	0.02837	ELOA
4714.58807	-0.40774	0.12089	-3.37281	0.00074	0.02850	LHFPL2
15.67832	-2.35042	0.69717	-3.37135	0.00075	0.02850	PSMA1
152.00108	-1.61060	0.47851	-3.36586	0.00076	0.02890	ABCG2
2081.53641	-0.73033	0.21752	-3.35751	0.00079	0.02961	EFEMP1
4.58267	-5.11562	1.52861	-3.34658	0.00082	0.03060	PSG5
724.88493	4.60726	1.37731	3.34511	0.00082	0.03060	TDG
33.98830	-1.05334	0.31521	-3.34175	0.00083	0.03079	IL17A
15.72351	2.34116	0.70234	3.33338	0.00086	0.03155	RPS3
23.96568	3.61755	1.08669	3.32896	0.00087	0.03187	SRGAP3
29.86779	2.86512	0.86169	3.32502	0.00088	0.03214	NOTCH3
68.89408	2.32803	0.70083	3.32181	0.00089	0.03233	SRP68
55.38921	-1.68468	0.50927	-3.30806	0.00094	0.03362	STAG2
149.02245	2.01781	0.61003	3.30773	0.00094	0.03362	PLPP3
683.05674	1.89129	0.57219	3.30535	0.00095	0.03371	KRT31
854.93336	-0.51384	0.15563	-3.30161	0.00096	0.03398	CLDN9
50.81670	2.08699	0.63360	3.29384	0.00099	0.03474	CTRC
1596.96608	-0.39644	0.12047	-3.29075	0.00100	0.03493	TCOF1
60.35446	-2.31268	0.70335	-3.28807	0.00101	0.03507	CFL1
3.27049	4.18688	1.27601	3.28124	0.00103	0.03554	RNASE6
2740.61706	2.50490	0.76342	3.28116	0.00103	0.03554	RNF217-AS1
1696.28072	-0.68503	0.20887	-3.27974	0.00104	0.03554	NUP155
47.76296	-1.85831	0.56973	-3.26172	0.00111	0.03747	POMT1
25902.55528	-1.56264	0.47907	-3.26179	0.00111	0.03747	KLK7
961.60339	1.44135	0.44358	3.24933	0.00116	0.03894	SGK2
14.85237	2.88035	0.88696	3.24744	0.00116	0.03899	SGPL1
63.56152	-2.10669	0.65101	-3.23601	0.00121	0.03984	IFITM3
30.82336	-2.47744	0.76481	-3.23930	0.00120	0.03984	NOS1
40.65655	-0.75685	0.23393	-3.23534	0.00121	0.03984	GPR137B
8361.46484	1.18889	0.36746	3.23540	0.00121	0.03984	PTER
81.02504	-1.28375	0.39745	-3.22996	0.00124	0.04039	MAN2A1
2.15459	3.23342	1.00232	3.22595	0.00126	0.04075	AK2
458.22324	-0.52545	0.16304	-3.22290	0.00127	0.04098	KCNJ16
2921.21999	1.24167	0.38614	3.21557	0.00130	0.04183	SLC8A3
41.03044	2.86292	0.89102	3.21308	0.00131	0.04198	CCR5
809.49831	-0.77525	0.24150	-3.21016	0.00133	0.04199	SIGMAR1
96.40844	-2.31989	0.72242	-3.21130	0.00132	0.04199	CYTH3
27.85894	2.61753	0.81590	3.20815	0.00134	0.04207	RRAGA

20.08204	5.43815	1.70019	3.19855	0.00138	0.04329	ZNF224
83.99878	-1.59910	0.50054	-3.19474	0.00140	0.04330	SPIN1
622.14620	-0.66834	0.20928	-3.19352	0.00141	0.04330	HSPA1B
129.95069	-0.93046	0.29142	-3.19286	0.00141	0.04330	ME1
623.01826	1.52139	0.47629	3.19422	0.00140	0.04330	SSX2
488.61323	-0.81288	0.25482	-3.19003	0.00142	0.04351	MAL
28.14951	-3.29761	1.03661	-3.18117	0.00147	0.04445	ADAMTS7
2.40121	-3.78763	1.19067	-3.18108	0.00147	0.04445	CD74
69.49696	1.15678	0.36445	3.17402	0.00150	0.04533	PEX1
447.88469	-2.47845	0.78165	-3.17081	0.00152	0.04562	PTGDR
98.95668	-2.44081	0.77020	-3.16907	0.00153	0.04566	GRIN2A
4958.19757	1.15104	0.36335	3.16781	0.00154	0.04566	PMM2
367.24683	1.50712	0.47710	3.15892	0.00158	0.04671	TOPORS
112.33107	-2.36187	0.74779	-3.15848	0.00159	0.04671	RTN3
1630.25081	-0.47107	0.14941	-3.15274	0.00162	0.04742	PRKRA
1687.64828	-0.48477	0.15445	-3.13863	0.00170	0.04954	SYNGR3
118.49764	2.15771	0.68833	3.13470	0.00172	0.04998	HPR
67.96156	7.98280	2.55077	3.12957	0.00175	0.05019	MAD2L1
48.52210	-2.28150	0.72904	-3.12947	0.00175	0.05019	MTM1
5570.81776	2.28232	0.72921	3.12986	0.00175	0.05019	PMS2P1
24.37528	3.21143	1.02812	3.12359	0.00179	0.05097	LAMC2
2017.81158	-0.35817	0.11472	-3.12227	0.00179	0.05097	H2AC17
28.54164	2.33983	0.74972	3.12093	0.00180	0.05098	HTN1
50.95468	0.83932	0.26929	3.11679	0.00183	0.05145	CCR3
58.63982	-0.96593	0.31003	-3.11561	0.00184	0.05145	MXD1
373.65306	-0.94847	0.30464	-3.11341	0.00185	0.05161	MIA2
102.74404	-1.56718	0.50367	-3.11152	0.00186	0.05167	APC2
26.91519	2.32066	0.74609	3.11044	0.00187	0.05167	NME3
937.50946	1.26252	0.40639	3.10668	0.00189	0.05211	FES
412.56852	-1.56861	0.50514	-3.10532	0.00190	0.05212	BCAS2
191.48993	3.02880	0.97690	3.10043	0.00193	0.05276	PABPC4
29.35681	-1.01686	0.32911	-3.08972	0.00200	0.05447	VPS45
2294.05577	-0.45766	0.14836	-3.08489	0.00204	0.05489	FNTA
407.39252	-0.33344	0.10807	-3.08529	0.00203	0.05489	UQCRC1
7737.13643	0.86969	0.28206	3.08333	0.00205	0.05495	GTPBP1
97.03617	1.57617	0.51155	3.08116	0.00206	0.05512	GSS
3472.95081	-1.63700	0.53234	-3.07509	0.00210	0.05602	RCN1
14.82762	2.06823	0.67420	3.06769	0.00216	0.05625	TRIOBP
9561.80055	-0.62780	0.20450	-3.06985	0.00214	0.05625	H2BC5
4.42818	4.91334	1.59940	3.07199	0.00213	0.05625	MAGEA1
256.64251	-1.39346	0.45419	-3.06802	0.00215	0.05625	RP9
332.21805	0.81060	0.26412	3.06904	0.00215	0.05625	IL18R1
423.18397	1.79446	0.58531	3.06586	0.00217	0.05636	XRCC5
82.61911	-1.76868	0.57752	-3.06255	0.00219	0.05676	GSTA3
629.22070	-0.81742	0.26752	-3.05552	0.00225	0.05763	SP110
123.16795	1.89661	0.62049	3.05662	0.00224	0.05763	NFATC2
10.91380	-2.47128	0.80965	-3.05229	0.00227	0.05779	HK3
70.05864	-0.92585	0.30322	-3.05340	0.00226	0.05779	ENTPD6

284.01822	-0.98972	0.32480	-3.04720	0.00231	0.05808	HDAC5
347.85717	3.42517	1.12394	3.04746	0.00231	0.05808	ITGB2
2268.18379	-0.47637	0.15631	-3.04765	0.00231	0.05808	ATXN1
2.87883	2.56482	0.84262	3.04386	0.00234	0.05819	SH2D3C
424.79385	-0.96412	0.31682	-3.04309	0.00234	0.05819	MBL2
17839.86484	1.57495	0.51731	3.04452	0.00233	0.05819	GMFG
283.51920	1.56212	0.51452	3.03610	0.00240	0.05933	MAP4
13.61655	1.71674	0.56592	3.03355	0.00242	0.05960	ARFGEF2
869.03057	-0.66233	0.21857	-3.03026	0.00244	0.05991	CSN3
6.67866	2.90165	0.95776	3.02963	0.00245	0.05991	ZNF91
4983.70499	-0.44776	0.14803	-3.02475	0.00249	0.06065	TAAR2
171.97812	-1.78273	0.59001	-3.02152	0.00252	0.06107	ATP6AP1
3402.62945	0.95075	0.31519	3.01643	0.00256	0.06164	PSMD14
47.82185	2.07566	0.68807	3.01662	0.00256	0.06164	MED26
41.95497	2.61906	0.86871	3.01487	0.00257	0.06172	FCGBP
1401.11934	1.42100	0.47330	3.00230	0.00268	0.06409	POLE2
101.19598	-0.89243	0.29773	-2.99742	0.00272	0.06488	FHL3
35.01906	1.32958	0.44437	2.99210	0.00277	0.06577	SLC35E2A
158.17979	1.65343	0.55293	2.99030	0.00279	0.06592	RGS2
8855.32950	0.39663	0.13273	2.98815	0.00281	0.06613	RBPMS
1503.82925	-0.46247	0.15484	-2.98680	0.00282	0.06618	WNT10B
23.79570	-4.65818	1.56134	-2.98345	0.00285	0.06638	MAP4K5
67.06385	1.39571	0.46796	2.98251	0.00286	0.06638	H3C11
260.13074	-0.73248	0.24551	-2.98351	0.00285	0.06638	CRYZL1
363.32402	1.10519	0.37078	2.98071	0.00288	0.06645	STAU1
1683.87820	-0.36673	0.12307	-2.97998	0.00288	0.06645	BAG6
1149.97485	-1.17478	0.39470	-2.97642	0.00292	0.06698	NUDT5
1654.32963	0.43742	0.14702	2.97533	0.00293	0.06698	HGS
693.12271	0.60319	0.20294	2.97223	0.00296	0.06742	HBQ1
3480.26410	-0.32103	0.10836	-2.96264	0.00305	0.06930	ZNF33B
24.77356	-1.04883	0.35452	-2.95844	0.00309	0.06998	AP2B1
9.29131	8.75519	2.96140	2.95643	0.00311	0.06998	GSR
4.65492	-1.93148	0.65333	-2.95635	0.00311	0.06998	PTCH2
4018.75431	0.64949	0.21982	2.95462	0.00313	0.07013	HTR3B
204.43622	3.52489	1.19498	2.94974	0.00318	0.07100	PARP2
891.38743	1.40897	0.47825	2.94608	0.00322	0.07159	FASTK
339.00659	2.36079	0.80204	2.94349	0.00325	0.07169	ADRB3
1205.36701	-0.42925	0.14579	-2.94428	0.00324	0.07169	SI
4062.96953	-1.06901	0.36359	-2.94016	0.00328	0.07172	FANCE
15.57923	2.76370	0.93968	2.94109	0.00327	0.07172	GUCY2D
11.96843	-2.19258	0.74547	-2.94119	0.00327	0.07172	TNFRSF17
805.03408	0.49547	0.16865	2.93793	0.00330	0.07174	DLC1
17.89974	1.65306	0.56257	2.93840	0.00330	0.07174	NDUFB4
26.62466	-0.75901	0.25850	-2.93620	0.00332	0.07190	ADAM15
390.71082	-1.19245	0.40715	-2.92880	0.00340	0.07338	LTK
7228.99402	-0.42211	0.14433	-2.92453	0.00345	0.07415	SLC22A3
9.16262	4.89008	1.67309	2.92278	0.00347	0.07426	TRAF3IP2
1083.23248	1.37610	0.47095	2.92195	0.00348	0.07426	DTNA

452.52625	0.90615	0.31076	2.91595	0.00355	0.07545	CHD3
21.66162	-0.87477	0.30064	-2.90966	0.00362	0.07673	PSMD2
8.58821	2.10930	0.72568	2.90664	0.00365	0.07716	AP2A1
39.15916	-0.90447	0.31126	-2.90584	0.00366	0.07716	BYSL
1798.22829	-0.39779	0.13706	-2.90241	0.00370	0.07775	SNRNP70
10.13221	8.58685	2.96252	2.89850	0.00375	0.07847	ABCC9
989.79134	-0.54627	0.18892	-2.89152	0.00383	0.07997	TM4SF5
106.10811	2.43858	0.84392	2.88957	0.00386	0.08006	MOCS2
9.45655	-3.13471	1.08538	-2.88811	0.00388	0.08006	ACTA1
2272.22525	-0.49361	0.17091	-2.88809	0.00388	0.08006	NUMB
14.59138	-2.52527	0.87490	-2.88634	0.00390	0.08025	RFC1
1215.66809	-0.43073	0.14932	-2.88450	0.00392	0.08046	SMCP
6162.49522	0.48703	0.16897	2.88234	0.00395	0.08075	EPHB6
13.92728	8.56414	2.97445	2.87923	0.00399	0.08129	HMBS
3.00382	4.78372	1.66229	2.87778	0.00400	0.08140	CYP19A1
20.48236	2.17243	0.75549	2.87554	0.00403	0.08172	NHS
48.76144	-1.26950	0.44165	-2.87444	0.00405	0.08175	SCN9A
669.25308	1.27205	0.44305	2.87110	0.00409	0.08235	RPL36AL
216.08455	2.85871	0.99629	2.86935	0.00411	0.08255	POLR2B
153.01126	-0.62100	0.21703	-2.86141	0.00422	0.08438	HOXC5
9539.51238	0.40335	0.14106	2.85946	0.00424	0.08439	DLX3
808.35741	0.70128	0.24542	2.85742	0.00427	0.08439	GMDS
33.66315	-2.20982	0.77303	-2.85866	0.00425	0.08439	NFIL3
420.13522	-0.46252	0.16181	-2.85836	0.00426	0.08439	PECAM1
8.63529	3.61523	1.26711	2.85312	0.00433	0.08527	RAB6A
2049.52124	-0.80309	0.28193	-2.84852	0.00439	0.08604	CBLIF
18.93741	-2.19967	0.77228	-2.84829	0.00440	0.08604	RNF112
36.72027	3.00073	1.05459	2.84538	0.00444	0.08657	RPL23
76.70069	-1.32575	0.46682	-2.83997	0.00451	0.08778	CST6
25.41865	3.28493	1.15708	2.83898	0.00453	0.08778	ZNF22
378.43793	-0.40541	0.14291	-2.83673	0.00456	0.08814	PSG1
34.04881	4.65193	1.64366	2.83022	0.00465	0.08968	TAP2
58.99714	-2.06370	0.73030	-2.82581	0.00472	0.09065	PTGS2
39.84553	1.91896	0.67950	2.82407	0.00474	0.09087	SGSM2
1141.92724	-0.42269	0.14985	-2.82078	0.00479	0.09100	ERCC5
800.10716	1.99423	0.70678	2.82155	0.00478	0.09100	RAB2A
14.86899	2.92852	1.03822	2.82071	0.00479	0.09100	ELOC
106582.96110	1.40651	0.49893	2.81903	0.00482	0.09121	SP4
97.69516	1.81694	0.64483	2.81770	0.00484	0.09125	MSH3
4144.80413	-0.67178	0.23848	-2.81697	0.00485	0.09125	PCYT1A
42.27377	2.39562	0.85126	2.81420	0.00489	0.09150	TIMM44
3728.50155	-1.60203	0.56918	-2.81463	0.00488	0.09150	ELN
8.01582	2.43111	0.86628	2.80637	0.00501	0.09346	MRC1
2343.06312	-0.47911	0.17078	-2.80548	0.00502	0.09346	RP1
798.70889	-2.45483	0.87678	-2.79982	0.00511	0.09484	WFDC2
954.64314	1.44094	0.51510	2.79739	0.00515	0.09528	ADRA1D
2087.47517	1.11843	0.40034	2.79371	0.00521	0.09609	PSMB3
14.57085	-1.51983	0.54429	-2.79232	0.00523	0.09622	MAP3K13

52.11962	2.02818	0.72717	2.78916	0.00528	0.09677	GABARAP
97.85051	-0.73127	0.26223	-2.78863	0.00529	0.09677	DHCR24
15.98068	-1.71758	0.61627	-2.78708	0.00532	0.09678	EPHA7
808.58782	-0.47735	0.17129	-2.78674	0.00532	0.09678	PSKH1
685.08357	-0.49360	0.17738	-2.78266	0.00539	0.09751	EEF2
786.10298	-0.58870	0.21174	-2.78022	0.00543	0.09751	MME
2537.89933	-0.40308	0.14499	-2.78012	0.00543	0.09751	PHF2
24.93103	1.45067	0.52219	2.77804	0.00547	0.09751	PLAGL1
479.61427	1.40729	0.50655	2.77821	0.00547	0.09751	PPARG
4081.94704	0.86851	0.31266	2.77786	0.00547	0.09751	SIAH1
942.24242	0.66566	0.23950	2.77937	0.00545	0.09751	TCF20
7.31450	3.39117	1.22232	2.77437	0.00553	0.09829	TLE2
1383.55352	-0.78644	0.28356	-2.77342	0.00555	0.09830	SOX1
320.13158	1.66987	0.60328	2.76797	0.00564	0.09968	HLA-DRB3
1805.75744	-0.46808	0.16919	-2.76660	0.00566	0.09983	RPS21
183.51063	1.78663	0.64679	2.76231	0.00574	0.10039	GPR37
12286.09815	0.48322	0.17484	2.76386	0.00571	0.10039	GUK1
8.62788	-3.20708	1.16111	-2.76208	0.00574	0.10039	HSPA1L
792.93821	1.40382	0.50848	2.76081	0.00577	0.10050	ACTR2
59.30462	1.28080	0.46445	2.75767	0.00582	0.10092	FXR1
603.14930	-0.69767	0.25297	-2.75795	0.00582	0.10092	RIOK3
819.88300	-0.58299	0.21155	-2.75580	0.00585	0.10122	BCS1L
14.56970	-2.53607	0.92127	-2.75280	0.00591	0.10153	AKAP8
192.55840	-0.67362	0.24478	-2.75197	0.00592	0.10153	MLANA
1063.59504	-0.56848	0.20653	-2.75253	0.00591	0.10153	HOXD1
58.54485	-2.20766	0.80242	-2.75126	0.00594	0.10153	PITX1
243.94274	-0.76352	0.27784	-2.74808	0.00599	0.10197	MOBP
2.34515	-3.01627	1.09757	-2.74814	0.00599	0.10197	PRPF3
20.33349	-0.98545	0.35909	-2.74432	0.00606	0.10287	OAT
15.54983	4.85248	1.76883	2.74333	0.00608	0.10291	PIK3C2A
11.23970	-3.17877	1.15973	-2.74097	0.00613	0.10327	GJB6
145.82662	-1.13633	0.41470	-2.74014	0.00614	0.10327	IFNA10
5657.77903	0.53896	0.19679	2.73870	0.00617	0.10327	TLE4
55.43800	1.16161	0.42415	2.73870	0.00617	0.10327	CD38
5.42880	-3.47377	1.26956	-2.73620	0.00622	0.10355	ATP5F1A
22.71611	3.10730	1.13567	2.73609	0.00622	0.10355	ZNF138
442.71825	-1.98266	0.72506	-2.73446	0.00625	0.10372	MMP8
74.29371	1.26482	0.46266	2.73384	0.00626	0.10372	SSPN
5.90753	4.17737	1.53064	2.72917	0.00635	0.10418	AHSA1
3.18239	1.73522	0.63586	2.72895	0.00635	0.10418	MCAM
6.08433	1.69711	0.62182	2.72926	0.00635	0.10418	ATP1B2
3343.04076	0.44310	0.16228	2.73048	0.00632	0.10418	SPP1
2366.15037	-0.57491	0.21081	-2.72712	0.00639	0.10422	POLRMT
12.76885	2.29135	0.83995	2.72795	0.00637	0.10422	UPK1B
998.19986	0.99810	0.36648	2.72349	0.00646	0.10465	SSX3
16.23454	-1.16584	0.42811	-2.72323	0.00646	0.10465	GNAL
394.24614	1.20304	0.44159	2.72431	0.00644	0.10465	NUMBL
929.33448	1.99084	0.73162	2.72113	0.00651	0.10505	PMP22

389.82092	-0.53935	0.19830	-2.71982	0.00653	0.10520	RAD23A
217.65060	-1.57509	0.57954	-2.71783	0.00657	0.10555	DRD1
3167.70188	-0.55623	0.20472	-2.71706	0.00659	0.10555	KPNA2
9689.35911	0.45643	0.16805	2.71601	0.00661	0.10562	HCG9
12.43465	-2.16391	0.79700	-2.71508	0.00663	0.10565	DNALI1
1146.61927	-0.75409	0.27801	-2.71242	0.00668	0.10590	ERBB3
5301.20635	-0.84135	0.31025	-2.71184	0.00669	0.10590	GLDC
321.62847	-0.87146	0.32138	-2.71161	0.00670	0.10590	PPM1D
749.73491	-1.40642	0.51878	-2.71099	0.00671	0.10590	MAGED1
97.34644	-0.76472	0.28255	-2.70649	0.00680	0.10639	ALDOB
291.63397	2.28866	0.84519	2.70788	0.00677	0.10639	CTSA
6454.50583	1.15159	0.42554	2.70619	0.00681	0.10639	RGS7
127.85890	-0.85355	0.31531	-2.70705	0.00679	0.10639	CDC16
787.57069	0.33861	0.12524	2.70376	0.00686	0.10641	DSP
843.91060	-0.61307	0.22676	-2.70368	0.00686	0.10641	HLA-DPB2
316.98866	-0.72034	0.26642	-2.70374	0.00686	0.10641	TECTA
121.99500	-0.71196	0.26354	-2.70157	0.00690	0.10670	MECP2
738.76119	1.16074	0.42972	2.70114	0.00691	0.10670	TRPM1
259.67499	-1.09417	0.40601	-2.69494	0.00704	0.10741	ELF1
60.94928	-1.14732	0.42561	-2.69574	0.00702	0.10741	ABCA3
750.92407	-1.21490	0.45056	-2.69640	0.00701	0.10741	PPARA
777.96161	-0.47133	0.17485	-2.69570	0.00702	0.10741	RAG1
9.98932	3.87685	1.43849	2.69507	0.00704	0.10741	RPS10
259.59715	1.03710	0.38497	2.69399	0.00706	0.10746	CTIF
34.86915	1.51809	0.56460	2.68880	0.00717	0.10870	IL2RG
55.45453	-1.14299	0.42513	-2.68857	0.00718	0.10870	C8A
157.78314	2.58610	0.96229	2.68744	0.00720	0.10881	SMYD5
133.42035	-0.82146	0.30595	-2.68494	0.00725	0.10936	IRAK3
472.97008	1.14075	0.42506	2.68378	0.00728	0.10949	CD151
59.81113	-1.18898	0.44324	-2.68246	0.00731	0.10966	MYO1C
1496.24466	-0.44633	0.16648	-2.68098	0.00734	0.10989	FGF23
699.66963	1.56460	0.58381	2.68001	0.00736	0.10990	IRF6
1448.15727	1.85609	0.69274	2.67936	0.00738	0.10990	CFP
1101.84665	1.05870	0.39548	2.67701	0.00743	0.11004	GLB1
78.50730	-1.80298	0.67361	-2.67660	0.00744	0.11004	BACH1
168.77789	-0.70954	0.26507	-2.67679	0.00743	0.11004	COIL
2958.59656	-0.38775	0.14514	-2.67158	0.00755	0.11067	LHB
2357.59456	0.58073	0.21734	2.67204	0.00754	0.11067	XDH
7.30603	7.00663	2.62119	2.67307	0.00752	0.11067	AKAP17A
17.92671	1.45818	0.54579	2.67166	0.00755	0.11067	PIAS2
2860.86666	-0.37510	0.14075	-2.66491	0.00770	0.11263	GHRHR
9.59321	1.88385	0.70713	2.66406	0.00772	0.11265	H3C8
7.48997	4.12023	1.54766	2.66223	0.00776	0.11301	FOXG1
1734.36437	0.87154	0.32758	2.66052	0.00780	0.11328	MOK
760.12325	-0.63111	0.23727	-2.65989	0.00782	0.11328	SMARCA5
109.62229	-1.55481	0.58477	-2.65883	0.00784	0.11338	CXCL11
1172.30709	-0.93566	0.35237	-2.65538	0.00792	0.11428	ZNF2
2925.94042	-0.41713	0.15713	-2.65463	0.00794	0.11428	MAP3K6

61.49693	1.49540	0.56393	2.65175	0.00801	0.11429	ERCC6
4428.86867	0.44431	0.16755	2.65180	0.00801	0.11429	EZH1
1847.63330	-0.50013	0.18869	-2.65055	0.00804	0.11429	F2RL1
12.92728	-1.04800	0.39542	-2.65031	0.00804	0.11429	GLI2
5.14247	-6.78967	2.56428	-2.64779	0.00810	0.11429	ARL2
22.15237	-1.11182	0.41935	-2.65129	0.00802	0.11429	MIF
2570.20795	-0.45740	0.17266	-2.64907	0.00807	0.11429	SMARCD2
635.12776	-0.36304	0.13701	-2.64973	0.00806	0.11429	XBP1
89.20459	1.48653	0.56131	2.64833	0.00809	0.11429	USO1
162.52607	2.45954	0.92923	2.64686	0.00812	0.11435	USP1
4042.54105	-0.27059	0.10234	-2.64407	0.00819	0.11448	GNAS
1537.81174	0.78895	0.29853	2.64280	0.00822	0.11448	NDUFA8
13.00895	-1.69416	0.64072	-2.64415	0.00819	0.11448	NPAS2
757.44993	2.08744	0.78904	2.64554	0.00816	0.11448	PIN1P1
2628.24373	-0.66844	0.25293	-2.64277	0.00822	0.11448	XKRY
10300.55280	-0.40041	0.15157	-2.64176	0.00825	0.11457	SPG7
163.09014	0.77878	0.29505	2.63952	0.00830	0.11508	RBM10
4107.06478	-0.75064	0.28447	-2.63868	0.00832	0.11511	NEDD8
2950.62814	-0.69209	0.26241	-2.63743	0.00835	0.11528	KDELR3
1056.69382	1.62163	0.61545	2.63488	0.00842	0.11540	LEPR
14417.89138	-0.69711	0.26455	-2.63511	0.00841	0.11540	SCN1A
259.26918	-0.55764	0.21163	-2.63498	0.00841	0.11540	ADGRE5
697.31695	2.15621	0.81875	2.63353	0.00845	0.11543	FOS
2450.05254	-0.60214	0.22866	-2.63335	0.00845	0.11543	RTKN
39.16686	2.78057	1.05655	2.63175	0.00849	0.11556	GJA5
4816.45743	-0.45352	0.17234	-2.63150	0.00850	0.11556	CCL14
1898.23024	0.75940	0.28875	2.62995	0.00854	0.11584	MED17
3.45052	3.97869	1.51412	2.62773	0.00860	0.11635	BAX
2870.78676	-0.42640	0.16255	-2.62321	0.00871	0.11741	XPC
49.77126	-3.00330	1.14475	-2.62354	0.00870	0.11741	CDC5L
85.61983	2.02462	0.77270	2.62019	0.00879	0.11810	HCLS1
5.94265	3.35405	1.28029	2.61976	0.00880	0.11810	NUP88
101.28329	3.54449	1.35347	2.61882	0.00882	0.11818	HAND2
190.54859	-1.13900	0.43507	-2.61795	0.00885	0.11823	TJP2
3635.41617	-0.53235	0.20357	-2.61503	0.00892	0.11900	NR2F1
335.29379	-0.74292	0.28439	-2.61233	0.00899	0.11967	RPS4X
21.32950	-1.15031	0.44045	-2.61168	0.00901	0.11967	RECQL4
6288.03854	-0.84796	0.32523	-2.60730	0.00913	0.11996	POP7
104.88015	-1.77009	0.67824	-2.60984	0.00906	0.11996	RPL35
86.24383	1.02996	0.39519	2.60622	0.00915	0.11996	ADRB2
2046.27973	-0.34742	0.13326	-2.60712	0.00913	0.11996	EPHB3
17.73855	3.54559	1.35884	2.60927	0.00907	0.11996	MR1
416.32358	-0.52528	0.20146	-2.60741	0.00912	0.11996	MEIS1
513.33723	-0.55453	0.21280	-2.60589	0.00916	0.11996	MYO1B
11.65803	2.31564	0.88992	2.60207	0.00927	0.12022	DAXX
219.31315	-0.61140	0.23501	-2.60163	0.00928	0.12022	RPSA
297.91447	0.56959	0.21903	2.60051	0.00931	0.12022	PRNP
141.86219	-1.20215	0.46166	-2.60396	0.00922	0.12022	RELN

3176.52317	-0.43745	0.16823	-2.60023	0.00932	0.12022	CCL4
2203.38378	-0.58314	0.22426	-2.60035	0.00931	0.12022	TNS1
267.86679	-0.95063	0.36536	-2.60188	0.00927	0.12022	RCE1
178.16741	-0.34177	0.13155	-2.59806	0.00938	0.12062	CELF1
9020.94683	0.96010	0.36960	2.59770	0.00938	0.12062	RNF13
50.50598	-1.47420	0.56779	-2.59639	0.00942	0.12084	AFF3
5009.75863	-0.85444	0.32927	-2.59498	0.00946	0.12085	KIF5C
4492.72864	0.78175	0.30134	2.59429	0.00948	0.12085	PSEN1
39.11272	-2.27726	0.87759	-2.59489	0.00946	0.12085	PDLIM1
76121.58292	-0.65760	0.25365	-2.59255	0.00953	0.12098	POU2F2
1180.05891	-0.26619	0.10268	-2.59253	0.00953	0.12098	SMO
1486.42754	-0.37141	0.14331	-2.59169	0.00955	0.12104	CSTF3
971.71206	-0.48692	0.18802	-2.58966	0.00961	0.12151	BCR
109.81817	2.53641	0.98012	2.58786	0.00966	0.12191	GDF15
418.51224	-0.79465	0.30744	-2.58475	0.00974	0.12197	PRSS3
1206.55235	0.63126	0.24405	2.58655	0.00969	0.12197	PTPN12
13941.40660	0.41901	0.16203	2.58601	0.00971	0.12197	RPS7
52.55173	-1.34835	0.52173	-2.58440	0.00975	0.12197	SH3GL1
2838.79545	-0.49539	0.19169	-2.58428	0.00976	0.12197	GPR55
162.63639	-1.58534	0.61387	-2.58254	0.00981	0.12234	IL1A
7.40868	2.38257	0.92358	2.57970	0.00989	0.12297	CYP2A7
171.54535	-1.02124	0.39592	-2.57943	0.00990	0.12297	ATF3
7159.52587	0.66942	0.25980	2.57670	0.00997	0.12370	PTGIR
79.17562	0.95163	0.36947	2.57566	0.01000	0.12384	TFPI
2366.02093	-1.37451	0.53392	-2.57436	0.01004	0.12406	NPY5R
2348.79283	1.90028	0.73940	2.57003	0.01017	0.12447	F11
510.01117	-1.11029	0.43175	-2.57161	0.01012	0.12447	FOXE3
18.25223	-1.83377	0.71322	-2.57110	0.01014	0.12447	HOXA9
29.70388	-0.92578	0.36008	-2.57103	0.01014	0.12447	INHBA
407.51853	1.84953	0.71977	2.56961	0.01018	0.12447	MLX
120.03361	-0.70996	0.27640	-2.56855	0.01021	0.12447	BFSP2
27.06915	1.68734	0.65684	2.56889	0.01020	0.12447	CD79A
749.53505	-0.46403	0.18090	-2.56505	0.01032	0.12514	TOM1L1
49.31939	1.30197	0.50762	2.56486	0.01032	0.12514	RFXAP
220.56659	-0.45878	0.17888	-2.56470	0.01033	0.12514	GGH
6364.58474	0.49356	0.19293	2.55821	0.01052	0.12639	OPTN
20.03107	-1.29441	0.50556	-2.56032	0.01046	0.12639	CRISP3
165.74593	-0.38710	0.15128	-2.55877	0.01050	0.12639	CENPF
218.23949	-1.21545	0.47517	-2.55794	0.01053	0.12639	IGF1R
18.38618	3.74309	1.46244	2.55948	0.01048	0.12639	MYRF
2465.14619	0.36596	0.14316	2.55622	0.01058	0.12678	EYA1
2950.51242	0.59305	0.23207	2.55552	0.01060	0.12680	EPHX1
25.91589	-1.02623	0.40169	-2.55478	0.01063	0.12683	NAE1
3.18294	-2.57312	1.00761	-2.55370	0.01066	0.12698	ZNF234
43.52025	1.52661	0.59803	2.55271	0.01069	0.12711	JUNB
196.15104	-0.35156	0.13786	-2.55005	0.01077	0.12728	AKT3
703.51229	-0.42886	0.16829	-2.54836	0.01082	0.12728	EVI2A
21.99000	3.75817	1.47391	2.54980	0.01078	0.12728	GRIA2

495.59799	-0.31441	0.12332	-2.54962	0.01078	0.12728	FOXO4
123.61613	1.66789	0.65411	2.54985	0.01078	0.12728	PDYN
59684.45293	0.97499	0.38257	2.54853	0.01082	0.12728	PRKCB
5.25067	-2.41495	0.94831	-2.54659	0.01088	0.12769	FICD
804.97766	-0.97760	0.38408	-2.54528	0.01092	0.12770	GABPA
1343.46326	-0.42767	0.16800	-2.54563	0.01091	0.12770	KIF5A
814.99757	2.49027	0.97918	2.54323	0.01098	0.12778	SEMA6C
83.32948	1.32081	0.51936	2.54313	0.01099	0.12778	KRT34
3191.70112	-0.38585	0.15170	-2.54356	0.01097	0.12778	CLPP
17213.15637	0.65761	0.25878	2.54124	0.01105	0.12824	TWF1
9.84665	-2.77393	1.09192	-2.54041	0.01107	0.12831	TRIM14
3848.63873	1.33817	0.52741	2.53725	0.01117	0.12924	SNRPN
530.55854	0.83478	0.32913	2.53633	0.01120	0.12934	SLC19A1
18.47774	2.30476	0.90909	2.53524	0.01124	0.12951	PFAS
185.42217	-0.59531	0.23498	-2.53347	0.01129	0.12993	BUB1B
498.85847	-0.52670	0.20811	-2.53088	0.01138	0.13037	MICU1
2279.60119	1.10762	0.43769	2.53062	0.01139	0.13037	DCTN3
131.10497	-0.58978	0.23308	-2.53040	0.01139	0.13037	CD19
27.38499	-0.87542	0.34606	-2.52966	0.01142	0.13041	SLC30A2
10.78327	-0.99235	0.39304	-2.52483	0.01158	0.13198	SEMA3B
8.45641	-1.89743	0.75193	-2.52343	0.01162	0.13203	CLPX
10.70236	-1.25103	0.49572	-2.52369	0.01161	0.13203	TSHR
3788.72790	0.72206	0.28638	2.52133	0.01169	0.13235	ELK4
95.58171	1.34126	0.53185	2.52185	0.01167	0.13235	SLC12A2
3416.98523	0.28741	0.11402	2.52060	0.01172	0.13239	ATXN2
15516.76925	0.35167	0.13960	2.51916	0.01176	0.13269	DHFRP3
3084.12554	0.53658	0.21337	2.51478	0.01191	0.13396	PMS1
1357.96783	-0.40996	0.16303	-2.51457	0.01192	0.13396	NAPA
242.72918	1.00384	0.39977	2.51103	0.01204	0.13483	ADIRF
3.64012	2.77137	1.10345	2.51155	0.01202	0.13483	PPIB
1397.57064	0.29374	0.11705	2.50967	0.01208	0.13507	ACKR1
5.81041	-3.01490	1.20165	-2.50896	0.01211	0.13507	ATXN3
276.67198	-0.50427	0.20110	-2.50759	0.01216	0.13507	TTC4
1281.21228	-0.50760	0.20241	-2.50780	0.01215	0.13507	ZNF84
6076.03747	0.80337	0.32041	2.50731	0.01217	0.13507	BPY2
5492.67795	-0.34563	0.13804	-2.50381	0.01229	0.13595	MTOR
1009.15218	-0.71252	0.28458	-2.50378	0.01229	0.13595	P4HA1
2488.80818	0.35689	0.14266	2.50164	0.01236	0.13607	PEX2
3.11491	3.02255	1.20790	2.50231	0.01234	0.13607	ZNF20
352.89188	-0.55599	0.22222	-2.50196	0.01235	0.13607	STX10
5.77108	2.27889	0.91178	2.49940	0.01244	0.13669	CCND1
85.72867	-1.21092	0.48466	-2.49847	0.01247	0.13682	FAM107A
239.09850	1.23395	0.49437	2.49599	0.01256	0.13754	DYNLT1
116.46919	1.40330	0.56269	2.49393	0.01263	0.13787	CDKN2C
79.66800	-0.51929	0.20822	-2.49397	0.01263	0.13787	NAT1
235.31586	1.18889	0.47744	2.49011	0.01277	0.13827	NSA2
2065.63171	-0.32530	0.13055	-2.49179	0.01271	0.13827	DSC1
909.69540	1.56740	0.62910	2.49148	0.01272	0.13827	RPL35A

5537.63874	-1.20979	0.48589	-2.48986	0.01278	0.13827	TRIM21
1002.29162	-0.46316	0.18601	-2.49000	0.01277	0.13827	CPNE1
12.32745	3.21081	1.29039	2.48824	0.01284	0.13867	NR2E3
787.48063	-0.43863	0.17650	-2.48520	0.01295	0.13915	GNRH2
5176.65580	-0.43357	0.17438	-2.48636	0.01291	0.13915	RAD52
1372.23737	-0.45201	0.18188	-2.48518	0.01295	0.13915	SMARCE1
17237.66815	-1.11445	0.44924	-2.48074	0.01311	0.14066	DNMT3B
120.56392	1.08079	0.43632	2.47709	0.01325	0.14187	COLEC10
382.81803	-2.36957	0.95803	-2.47338	0.01338	0.14263	GRK2
1797.29041	-0.55651	0.22495	-2.47395	0.01336	0.14263	LECT2
147.08138	-0.78241	0.31618	-2.47458	0.01334	0.14263	ARHGEF11
1736.30987	-0.71761	0.29036	-2.47141	0.01346	0.14318	EIF2B5
42.63927	-2.04152	0.82766	-2.46662	0.01364	0.14463	APBA2
267.21177	0.75474	0.30596	2.46676	0.01363	0.14463	SNRPA1
592.50842	-0.87143	0.35357	-2.46466	0.01371	0.14508	ARHGAP29
4064.20872	0.92624	0.37586	2.46430	0.01373	0.14508	LRIG2
179.28664	0.91349	0.37083	2.46335	0.01376	0.14522	CNIH1
63.78764	-1.07081	0.43489	-2.46222	0.01381	0.14544	RPL10A
1624.24501	-0.63095	0.25656	-2.45929	0.01392	0.14605	GNPDA1
1433.23355	0.51400	0.20903	2.45895	0.01393	0.14605	AURKA
175.44213	-0.53409	0.21713	-2.45974	0.01390	0.14605	IL1R2
1558.16726	1.42418	0.57962	2.45708	0.01401	0.14657	DHRS9
4078.92638	0.32966	0.13427	2.45521	0.01408	0.14709	EFNA4
53.28791	-0.66533	0.27124	-2.45292	0.01417	0.14779	GDI2
51.47795	0.84790	0.34581	2.45195	0.01421	0.14795	NOL3
207.42732	1.05829	0.43187	2.45049	0.01427	0.14830	AOAH
369.07382	-0.56779	0.23179	-2.44961	0.01430	0.14842	GPR18
25.80721	-0.92522	0.37824	-2.44609	0.01444	0.14964	MAGEB1
431.64833	-0.59799	0.24508	-2.43998	0.01469	0.15194	PITPNM1
107.97901	-1.12319	0.46058	-2.43867	0.01474	0.15200	KCNS2
7.82390	4.51133	1.84971	2.43894	0.01473	0.15200	RAB27A
196.45987	1.44736	0.59420	2.43580	0.01486	0.15297	SERPINC1
29.30346	1.00365	0.41230	2.43425	0.01492	0.15317	PRSS23
1557.15347	-0.56151	0.23068	-2.43414	0.01493	0.15317	MC3R
240.88350	-0.94249	0.38757	-2.43181	0.01502	0.15382	PTGER2
81.48819	-1.07333	0.44144	-2.43144	0.01504	0.15382	FGF16
3.13772	3.10652	1.28245	2.42234	0.01542	0.15712	ZNF277
38.83318	-0.72017	0.29727	-2.42262	0.01541	0.15712	GDNF
7.95085	1.97758	0.81653	2.42194	0.01544	0.15712	MAP4K3
3381.62972	-0.38668	0.15969	-2.42142	0.01546	0.15712	SH3BP5
2677.99732	-0.33051	0.13673	-2.41728	0.01564	0.15866	GCKR
440.72743	1.08042	0.44742	2.41479	0.01574	0.15897	MSLN
89.08426	3.01651	1.25035	2.41253	0.01584	0.15897	IPO7
15.66770	-3.20443	1.32795	-2.41308	0.01582	0.15897	ARHGDIA
67.01684	0.98538	0.40838	2.41288	0.01583	0.15897	PRPS2
1964.07366	0.22426	0.09289	2.41427	0.01577	0.15897	UGT2B10
8584.78367	0.73043	0.30275	2.41266	0.01584	0.15897	SLC24A1
3846.34778	0.34716	0.14373	2.41525	0.01572	0.15897	SRSF11

224.29581	-0.34886	0.14482	-2.40895	0.01600	0.16029	EIF4EBP3
4705.73200	0.56323	0.23388	2.40825	0.01603	0.16034	TYK2
71.21720	1.00763	0.41870	2.40654	0.01610	0.16084	PPID
5118.42700	0.62178	0.25853	2.40504	0.01617	0.16104	MYL6
3687.24360	-0.36934	0.15357	-2.40494	0.01618	0.16104	SPOCK1
3613.16319	-0.52194	0.21730	-2.40187	0.01631	0.16215	CNGA2
125.14308	1.14258	0.47594	2.40069	0.01636	0.16216	PI4KA
2778.67243	-0.39210	0.16330	-2.40108	0.01635	0.16216	UBE2B
12.84648	-2.03836	0.84941	-2.39975	0.01641	0.16233	LTF
226.31553	-0.60665	0.25290	-2.39873	0.01645	0.16253	TOP2A
5922.43037	0.51397	0.21432	2.39814	0.01648	0.16254	DNM2
1804.65391	-0.68605	0.28636	-2.39575	0.01659	0.16335	ACO1
37.51583	2.78811	1.16420	2.39486	0.01663	0.16349	MALT1
1726.80917	0.75123	0.31385	2.39361	0.01668	0.16366	SYT5
293.81280	-0.38026	0.15888	-2.39335	0.01670	0.16366	JAKMIP2
3511.20698	-0.42291	0.17675	-2.39276	0.01672	0.16367	SLC10A1
17.38719	-0.85297	0.35665	-2.39161	0.01677	0.16393	LILRB3
1351.44445	-0.54128	0.22640	-2.39085	0.01681	0.16402	ZNF213
431.99084	-1.58624	0.66392	-2.38918	0.01689	0.16426	ZNF202
105.23045	2.39671	1.00313	2.38922	0.01688	0.16426	MAPK8IP1
4.49903	2.07243	0.86815	2.38717	0.01698	0.16433	STAT2
6.45502	-2.83897	1.18937	-2.38696	0.01699	0.16433	CHAF1B
3259.12727	0.22500	0.09427	2.38678	0.01700	0.16433	INPP4B
1192.28345	-0.38803	0.16250	-2.38789	0.01695	0.16433	AKAP12
6.64721	1.46655	0.61497	2.38477	0.01709	0.16499	MT1X
626.94546	0.34507	0.14476	2.38381	0.01713	0.16517	HS2ST1
15.86757	-1.69139	0.71029	-2.38126	0.01725	0.16556	NEUROG1
1290.22436	0.42445	0.17819	2.38200	0.01722	0.16556	TACR1
197.88159	0.78999	0.33175	2.38127	0.01725	0.16556	TFAP2C
134.58040	-0.83514	0.35136	-2.37689	0.01746	0.16682	MEGF8
378.35989	-0.79644	0.33517	-2.37626	0.01749	0.16682	ETV3
176.26774	2.69911	1.13528	2.37749	0.01743	0.16682	PML
1388.87377	-0.39200	0.16496	-2.37629	0.01749	0.16682	TMBIM6
166.44836	1.28534	0.54155	2.37344	0.01762	0.16693	GTF2I
17.58613	-1.64984	0.69514	-2.37338	0.01763	0.16693	IDH3G
431.80467	0.64152	0.27006	2.37543	0.01753	0.16693	ITPA
939.12783	0.41102	0.17319	2.37327	0.01763	0.16693	KCNJ15
26.36356	-1.09874	0.46297	-2.37324	0.01763	0.16693	PIR
1381.44084	0.30809	0.13001	2.36978	0.01780	0.16810	TADA2A
2382.69025	-0.30457	0.12853	-2.36956	0.01781	0.16810	UBA3
10381.35986	0.32476	0.13718	2.36736	0.01792	0.16886	DOCK3
3198.37122	-0.47260	0.19983	-2.36500	0.01803	0.16968	S100A2
266.65721	-0.39213	0.16585	-2.36430	0.01806	0.16975	PDE9A
233.77081	0.66362	0.28093	2.36219	0.01817	0.17047	ITGB5
67.65203	-0.58853	0.24928	-2.36089	0.01823	0.17082	LUM
4223.95252	-0.25653	0.10878	-2.35830	0.01836	0.17176	EP300
14.21221	1.39000	0.59020	2.35515	0.01852	0.17297	PAIP1
544.89399	-0.33006	0.14032	-2.35222	0.01866	0.17408	KCND1

1199.30845	0.44559	0.18959	2.35031	0.01876	0.17473	TMCC2
1866.30736	-0.43384	0.18464	-2.34969	0.01879	0.17476	SLC7A1
40.72424	-0.96477	0.41102	-2.34723	0.01891	0.17566	TM4SF4
6210.96306	0.28319	0.12076	2.34500	0.01903	0.17599	GCLC
4.68455	-1.61057	0.68658	-2.34578	0.01899	0.17599	HAL
2557.26152	-0.44925	0.19158	-2.34491	0.01903	0.17599	PNMT
468.24069	-0.99065	0.42291	-2.34246	0.01916	0.17679	GALR1
114.11354	1.04851	0.44767	2.34213	0.01917	0.17679	EIF3I
297.54325	1.34883	0.57633	2.34039	0.01926	0.17710	ERCC4
618.70891	-0.79556	0.33988	-2.34072	0.01925	0.17710	SCNN1G
1761.94449	0.58913	0.25242	2.33392	0.01960	0.17737	EBI3
141.64696	1.99240	0.85268	2.33662	0.01946	0.17737	NET1
202.36608	-0.83424	0.35737	-2.33438	0.01958	0.17737	RCC1
1466.92816	0.76257	0.32646	2.33591	0.01950	0.17737	PHB2
1063.03723	-0.54070	0.23132	-2.33742	0.01942	0.17737	CNGA3
36.71273	-0.89490	0.38338	-2.33425	0.01958	0.17737	EFNA5
27.18320	-1.03648	0.44373	-2.33582	0.01950	0.17737	KIR2DL2
29.68521	1.15824	0.49547	2.33764	0.01941	0.17737	MT1F
5186.38021	-0.39098	0.16751	-2.33407	0.01959	0.17737	ACP3
29.36355	1.10630	0.47362	2.33583	0.01950	0.17737	PRKACA
413.33536	-0.58010	0.24831	-2.33623	0.01948	0.17737	PSME2
7.74150	2.49249	1.06902	2.33157	0.01972	0.17748	ITIH2
148.65557	1.39630	0.59871	2.33219	0.01969	0.17748	MYOG
72.09110	0.74900	0.32108	2.33278	0.01966	0.17748	RGS1
11.24361	-3.92010	1.68103	-2.33197	0.01970	0.17748	TCEA2
2197.83207	-0.35397	0.15200	-2.32872	0.01987	0.17859	TOB2
1595.02360	-0.22954	0.09859	-2.32813	0.01991	0.17862	DMTN
6.97643	3.36244	1.44487	2.32716	0.01996	0.17862	IL6
26.73227	-1.57598	0.67724	-2.32706	0.01996	0.17862	MAGEA5
91.19922	1.10231	0.47409	2.32509	0.02007	0.17891	USP20
4936.07021	-0.30988	0.13335	-2.32383	0.02013	0.17891	GPR35
3396.01746	0.47168	0.20294	2.32426	0.02011	0.17891	RPL29
1448.20597	0.82860	0.35634	2.32534	0.02005	0.17891	AIMP2
91.20254	-1.29699	0.55797	-2.32448	0.02010	0.17891	VGLL4
6698.28596	0.77778	0.33478	2.32328	0.02016	0.17892	RAC2
5214.24670	0.42207	0.18172	2.32260	0.02020	0.17899	LAD1
215.93941	1.41661	0.61032	2.32110	0.02028	0.17946	S100A5
286.28960	1.06000	0.45727	2.31809	0.02044	0.18065	ASAP2
14.12060	6.84779	2.95779	2.31517	0.02060	0.18155	UBA2
4.39829	3.27332	1.41374	2.31537	0.02059	0.18155	POLR2A
1533.02228	0.41395	0.17913	2.31090	0.02084	0.18311	SRSF3
110.00115	-1.19775	0.51828	-2.31100	0.02083	0.18311	H3-4
54.55748	1.10211	0.47747	2.30820	0.02099	0.18418	TFR2
4812.58075	-0.34463	0.14948	-2.30557	0.02113	0.18426	DRD5
3096.23331	-0.81178	0.35212	-2.30543	0.02114	0.18426	GPC3
11.05634	-0.98275	0.42610	-2.30640	0.02109	0.18426	APOC1P1
94.18258	-1.08039	0.46849	-2.30611	0.02110	0.18426	KCNA4
473.60892	-0.43685	0.18945	-2.30584	0.02112	0.18426	ITGB1BP1

63.49678	1.78379	0.77407	2.30444	0.02120	0.18449	FAM193A
727.69418	1.03098	0.44751	2.30382	0.02123	0.18454	RNF113A
3953.24736	0.33897	0.14722	2.30240	0.02131	0.18473	XG
25.62275	-1.44401	0.62710	-2.30266	0.02130	0.18473	CACNA1I
6.41652	-1.89339	0.82398	-2.29788	0.02157	0.18670	SRPX
590.73866	-0.47945	0.20920	-2.29183	0.02192	0.18717	TCIRG1
38.86879	1.76018	0.76765	2.29293	0.02185	0.18717	KLF12
56.01374	1.12495	0.49012	2.29527	0.02172	0.18717	HOXC6
1452.27616	0.40487	0.17646	2.29441	0.02177	0.18717	ROR1
23.95363	-1.29595	0.56519	-2.29294	0.02185	0.18717	ABCE1
702.04579	-0.38249	0.16696	-2.29089	0.02197	0.18717	SPAG4
717.21752	0.34193	0.14925	2.29102	0.02196	0.18717	TK1
819.44602	-0.42648	0.18582	-2.29514	0.02172	0.18717	ZFYVE9
1801.61600	1.04064	0.45362	2.29405	0.02179	0.18717	PSMF1
36.09522	-1.67168	0.72975	-2.29076	0.02198	0.18717	TTI1
4.87880	1.99729	0.87063	2.29407	0.02179	0.18717	KIAA0232
292.42481	-0.68818	0.30028	-2.29175	0.02192	0.18717	RNF40
16161.33824	0.81694	0.35692	2.28886	0.02209	0.18761	COPS6
7834.83240	-0.40750	0.17802	-2.28900	0.02208	0.18761	SLC28A1
913.29309	1.30216	0.56985	2.28510	0.02231	0.18922	HOXB1
642.98333	1.30765	0.57258	2.28377	0.02239	0.18963	PCDH8
6.31799	-1.18988	0.52127	-2.28267	0.02245	0.18968	ALDOC
27093.50033	0.33389	0.14627	2.28270	0.02245	0.18968	KIF23
15.47520	-1.39994	0.61388	-2.28049	0.02258	0.19017	CSNK1A1
41.81907	1.37199	0.60160	2.28056	0.02257	0.19017	KISS1
19.12886	1.43617	0.62985	2.28016	0.02260	0.19017	MMP12
5.00606	-3.01383	1.32251	-2.27888	0.02267	0.19056	TRIP6
13.99743	-1.15064	0.50508	-2.27811	0.02272	0.19069	SPOCK2
2376.92286	-0.24981	0.10971	-2.27698	0.02279	0.19075	F2RL3
132.21538	-1.25509	0.55112	-2.27733	0.02277	0.19075	FAM189A2
106.57955	1.48840	0.65397	2.27594	0.02285	0.19077	GMFB
2.38797	2.02449	0.88939	2.27628	0.02283	0.19077	IDI1
31.40666	0.82016	0.36047	2.27526	0.02289	0.19086	IL2
2.72527	3.31726	1.45948	2.27291	0.02303	0.19179	PARP3
5447.25347	-0.55397	0.24403	-2.27013	0.02320	0.19294	TPPP
12.99217	6.71932	2.96100	2.26928	0.02325	0.19312	INHA
206.04491	-0.61431	0.27078	-2.26867	0.02329	0.19317	CDH3
5909.62260	-0.76052	0.33551	-2.26678	0.02340	0.19384	GNG4
34008.92436	-0.44077	0.19448	-2.26635	0.02343	0.19384	PPP5C
236.34163	-0.50811	0.22424	-2.26586	0.02346	0.19384	ZNF155
5.87157	2.24913	0.99329	2.26432	0.02355	0.19411	IGF2BP1
1024.31426	0.29940	0.13221	2.26460	0.02354	0.19411	GJA1
270.19265	-0.65801	0.29080	-2.26275	0.02365	0.19466	CES2
890.59623	-0.41430	0.18333	-2.25987	0.02383	0.19568	DMBT1
16.32298	1.33775	0.59199	2.25975	0.02384	0.19568	LRP4
4872.07223	-0.22812	0.10101	-2.25848	0.02392	0.19607	GNA14
34.97595	-0.82247	0.36453	-2.25626	0.02405	0.19695	CRAT
40.66952	1.51353	0.67099	2.25566	0.02409	0.19701	JMJD7-PLA2G4B

1601.83577	-0.87598	0.38871	-2.25357	0.02422	0.19707	KERA
21.79952	2.56680	1.13998	2.25163	0.02435	0.19707	HRH3
2032.84805	0.74915	0.33237	2.25396	0.02420	0.19707	HNRNPA1
184.22908	-0.47547	0.21112	-2.25219	0.02431	0.19707	TNPO1
8.84078	2.05560	0.91238	2.25302	0.02426	0.19707	MGMT
2.64758	-3.21070	1.42781	-2.24869	0.02453	0.19707	NPAT
76.28309	1.18516	0.52683	2.24960	0.02447	0.19707	ARID4A
1066.99704	1.05923	0.47049	2.25132	0.02437	0.19707	SHH
2223.78352	-0.46889	0.20839	-2.25003	0.02445	0.19707	SIM2
276.86379	1.26463	0.56206	2.25001	0.02445	0.19707	SUMO2
24.95049	-0.90415	0.40168	-2.25092	0.02439	0.19707	LTBP4
70.90455	1.04329	0.46396	2.24867	0.02453	0.19707	EED
31.50528	-1.69397	0.75142	-2.25436	0.02417	0.19707	SLK
66.79681	3.99948	1.77789	2.24957	0.02448	0.19707	SV2B
83.43859	1.11571	0.49637	2.24771	0.02459	0.19713	MPV17
2768.95011	0.39171	0.17428	2.24759	0.02460	0.19713	RLN2
120.02403	1.59481	0.71100	2.24304	0.02489	0.19764	KPNA5
2807.43726	-0.36848	0.16417	-2.24445	0.02480	0.19764	KRTAP5-9
32.67262	-1.42807	0.63619	-2.24471	0.02479	0.19764	S100A8
504.36957	-0.50174	0.22354	-2.24455	0.02480	0.19764	SPINT1
14.15148	-0.91086	0.40614	-2.24273	0.02491	0.19764	TUBA4A
9.27819	-1.10248	0.49097	-2.24553	0.02473	0.19764	YWHAH
214.28244	-0.58626	0.26136	-2.24315	0.02489	0.19764	H4C3
403.10376	-0.66663	0.29705	-2.24416	0.02482	0.19764	DYRK2
3.01420	-1.47852	0.65941	-2.24218	0.02495	0.19767	SERTAD2
990.67828	-0.53042	0.23663	-2.24159	0.02499	0.19773	CCR8
103.04002	-1.13174	0.50513	-2.24048	0.02506	0.19805	FOXS1
155.73377	1.01685	0.45420	2.23878	0.02517	0.19843	GLIPR1
1290.20147	-0.32830	0.14662	-2.23912	0.02515	0.19843	SUPT5H
24.12705	-0.98056	0.43810	-2.23821	0.02521	0.19848	ARSL
502.30713	2.53270	1.13319	2.23502	0.02542	0.19975	SEMA4B
1740.76843	0.24711	0.11058	2.23468	0.02544	0.19975	STRN
236.41365	1.03374	0.46267	2.23430	0.02546	0.19975	TRIP12

Supplementary Table 6. Number of AID composite mutations per gene and per tumor type in the MSKCC cohort. This is related to Fig. 4a.

Hugo_Symbol	metamaintype	n_patients	n_composite	prop
EGFR	Melanoma	37	0	0
EGFR	Non-Small Cell Lung Cancer	931	10	0.010741138560687
EGFR	Breast Cancer	54	0	0
EGFR	Soft Tissue Sarcoma	9	0	0
EGFR	Colorectal Cancer	27	0	0
EGFR	Pancreatic Cancer	11	0	0
EGFR	Bladder Cancer	22	0	0
EGFR	Endometrial Cancer	14	0	0
EGFR	Glioma	191	17	0.089005235602094

EGFR	Cancer of Unknown Primary	16	0	0
EGFR	Appendiceal Cancer	2	0	0
EGFR	Bone Cancer	1	0	0
EGFR	Renal Cell Carcinoma	4	0	0
EGFR	Germ Cell Tumor	5	0	0
EGFR	Ovarian Cancer	7	0	0
EGFR	Salivary Gland Cancer	2	0	0
EGFR	Small Cell Lung Cancer	19	0	0
EGFR	Esophagogastric Cancer	12	0	0
EGFR	Head and Neck Cancer	6	0	0
EGFR	Skin Cancer, Non-Melanoma	4	0	0
EGFR	Thyroid Cancer	1	0	0
EGFR	Prostate Cancer	13	0	0
EGFR	Peripheral Nervous System	2	0	0
EGFR	Gastrointestinal Stromal Tumor	3	0	0
EGFR	Hepatobiliary Cancer	8	0	0
EGFR	Uterine Sarcoma	2	0	0
FBXW7	Pancreatic Cancer	18	0	0
FBXW7	Breast Cancer	30	0	0
FBXW7	Bladder Cancer	38	1	0.026315789473684
FBXW7	Endometrial Cancer	114	0	0
FBXW7	Ovarian Cancer	11	0	0
FBXW7	Colorectal Cancer	273	18	0.065934065934066
FBXW7	Small Cell Lung Cancer	5	0	0
FBXW7	Non-Small Cell Lung Cancer	71	1	0.014084507042254
FBXW7	Cancer of Unknown Primary	21	0	0
FBXW7	Esophagogastric Cancer	29	2	0.068965517241379
FBXW7	Melanoma	35	1	0.028571428571429
FBXW7	Appendiceal Cancer	8	0	0
FBXW7	Cervical Cancer	17	0	0
FBXW7	Bone Cancer	2	0	0
FBXW7	Head and Neck Cancer	25	0	0
FBXW7	Renal Cell Carcinoma	5	0	0
FBXW7	Peripheral Nervous System	1	0	0
FBXW7	Gastrointestinal Stromal Tumor	3	0	0
FBXW7	Salivary Gland Cancer	5	0	0
FBXW7	Gastrointestinal Neuroendocrine Tumor	3	0	0
FBXW7	Mesothelioma	9	0	0
FBXW7	Hepatobiliary Cancer	10	0	0
FBXW7	Soft Tissue Sarcoma	8	0	0
FBXW7	Glioma	10	0	0
FBXW7	Skin Cancer, Non-Melanoma	4	0	0
FBXW7	Thyroid Cancer	1	0	0
FBXW7	CNS Cancer	2	0	0

FBXW7	Prostate Cancer	3	0	0
FBXW7	Germ Cell Tumor	1	0	0
FBXW7	Uterine Sarcoma	2	0	0
FGFR3	Bladder Cancer	201	6	0.029850746268657
FGFR3	Glioma	11	0	0
FGFR3	Ovarian Cancer	5	0	0
FGFR3	Mesothelioma	1	0	0
FGFR3	Melanoma	21	0	0
FGFR3	Endometrial Cancer	3	0	0
FGFR3	Colorectal Cancer	13	0	0
FGFR3	Breast Cancer	18	0	0
FGFR3	Skin Cancer, Non-Melanoma	5	0	0
FGFR3	Non-Small Cell Lung Cancer	22	0	0
FGFR3	Cancer of Unknown Primary	11	0	0
FGFR3	Esophagogastric Cancer	3	0	0
FGFR3	Prostate Cancer	5	0	0
FGFR3	Pancreatic Cancer	3	0	0
FGFR3	Soft Tissue Sarcoma	5	0	0
FGFR3	Head and Neck Cancer	5	1	0.2
FGFR3	Salivary Gland Cancer	1	0	0
FGFR3	Renal Cell Carcinoma	6	0	0
FGFR3	Bone Cancer	3	0	0
FGFR3	Hepatobiliary Cancer	6	0	0
FGFR3	Small Cell Lung Cancer	1	0	0
FGFR3	Cervical Cancer	3	0	0
PIK3CA	Salivary Gland Cancer	24	1	0.041666666666667
PIK3CA	Colorectal Cancer	445	10	0.02247191011236
PIK3CA	Breast Cancer	1561	63	0.040358744394619
PIK3CA	Esophagogastric Cancer	57	2	0.035087719298246
PIK3CA	Non-Small Cell Lung Cancer	248	6	0.024193548387097
PIK3CA	Endometrial Cancer	413	18	0.043583535108959
PIK3CA	Bladder Cancer	155	4	0.025806451612903
PIK3CA	Ovarian Cancer	83	1	0.012048192771084
PIK3CA	Glioma	142	2	0.014084507042254
PIK3CA	Head and Neck Cancer	79	2	0.025316455696203
PIK3CA	Hepatobiliary Cancer	39	0	0
PIK3CA	Cervical Cancer	51	2	0.03921568627451
PIK3CA	Melanoma	28	0	0
PIK3CA	Cancer of Unknown Primary	85	1	0.011764705882353
PIK3CA	Bone Cancer	8	0	0
PIK3CA	Prostate Cancer	72	0	0
PIK3CA	Pancreatic Cancer	44	0	0
PIK3CA	Small Cell Lung Cancer	18	0	0
PIK3CA	Germ Cell Tumor	13	0	0
PIK3CA	Appendiceal Cancer	19	0	0

PIK3CA	Renal Cell Carcinoma	20	0	0
PIK3CA	Uterine Sarcoma	6	0	0
PIK3CA	Soft Tissue Sarcoma	36	0	0
PIK3CA	Skin Cancer, Non-Melanoma	24	1	0.041666666666667
PIK3CA	Thyroid Cancer	30	0	0
PIK3CA	Gastrointestinal Neuroendocrine Tumor	3	0	0
PIK3CA	Mesothelioma	5	0	0
PIK3CA	Gastrointestinal Stromal Tumor	7	0	0
PIK3CA	CNS Cancer	2	0	0
PTEN	Melanoma	79	0	0
PTEN	Glioma	334	1	0.002994011976048
PTEN	Germ Cell Tumor	17	0	0
PTEN	Breast Cancer	273	1	0.003663003663004
PTEN	Prostate Cancer	127	0	0
PTEN	Cancer of Unknown Primary	41	0	0
PTEN	Soft Tissue Sarcoma	29	0	0
PTEN	Ovarian Cancer	32	0	0
PTEN	Endometrial Cancer	349	14	0.040114613180516
PTEN	Esophagogastric Cancer	17	0	0
PTEN	Non-Small Cell Lung Cancer	125	0	0
PTEN	Renal Cell Carcinoma	39	1	0.025641025641026
PTEN	Colorectal Cancer	82	0	0
PTEN	Head and Neck Cancer	17	0	0
PTEN	Pancreatic Cancer	27	0	0
PTEN	Gastrointestinal Stromal Tumor	11	1	0.090909090909091
PTEN	Small Cell Lung Cancer	23	0	0
PTEN	Cervical Cancer	16	2	0.125
PTEN	Thyroid Cancer	33	0	0
PTEN	Bladder Cancer	15	0	0
PTEN	Mesothelioma	4	0	0
PTEN	Hepatobiliary Cancer	13	1	0.076923076923077
PTEN	Gastrointestinal Neuroendocrine Tumor	3	0	0
PTEN	Uterine Sarcoma	10	0	0
PTEN	Salivary Gland Cancer	5	0	0
PTEN	Bone Cancer	3	0	0
PTEN	Skin Cancer, Non-Melanoma	5	0	0
PTEN	Appendiceal Cancer	1	0	0
TP53	Prostate Cancer	492	0	0
TP53	Glioma	497	15	0.030181086519115
TP53	Endometrial Cancer	534	4	0.00749063670412
TP53	Pancreatic Cancer	1140	1	0.000877192982456
TP53	Non-Small Cell Lung Cancer	1988	12	0.006036217303823
TP53	Bladder Cancer	361	2	0.005540166204986

TP53	Ovarian Cancer	792	1	0.00126262626262626
TP53	Breast Cancer	1714	6	0.00350058343057 2
TP53	CNS Cancer	3	0	0
TP53	Colorectal Cancer	1986	19	0.00956696878147
TP53	Soft Tissue Sarcoma	240	4	0.0166666666666667
TP53	Cancer of Unknown Primary	437	2	0.004576659038902
TP53	Renal Cell Carcinoma	76	1	0.013157894736842
TP53	Small Cell Lung Cancer	235	0	0
TP53	Esophagogastric Cancer	590	8	0.013559322033898
TP53	Bone Cancer	56	0	0
TP53	Hepatobiliary Cancer	262	0	0
TP53	Head and Neck Cancer	207	9	0.043478260869565
TP53	Salivary Gland Cancer	65	0	0
TP53	Thyroid Cancer	84	2	0.023809523809524
TP53	Melanoma	139	1	0.007194244604317
TP53	Gastrointestinal Neuroendocrine Tumor	31	0	0
TP53	Germ Cell Tumor	57	0	0
TP53	Uterine Sarcoma	80	3	0.0375
TP53	Gastrointestinal Stromal Tumor	23	0	0
TP53	Appendiceal Cancer	62	0	0
TP53	Skin Cancer, Non-Melanoma	85	6	0.070588235294118
TP53	Cervical Cancer	26	1	0.038461538461539
TP53	Mesothelioma	35	1	0.028571428571429
TP53	Peripheral Nervous System	2	0	0

Supplementary Table 7. AID composite mutations' enrichment analysis per gene in the MSKCC cohort. This is related to Fig. 4b, top significant genes only.

Hugo Symbol	composites	samples	Predicted proportion	Observed proportion	p_enriched	p_twosided	logOR	Role	q_enriched	q_twosided
PIK3CA	115	3784	0.005	0.030	5.53x10^{-50}	5.53x10^{-50}	2.57	Oncogene	2.58x10^{-47}	2.58x10^{-47}
TP53	101	12423	0.003	0.008	4.32x10^{-17}	5.96x10^{-17}	1.39	TSG	1.01x10^{-14}	1.01x10^{-14}
FBXW7	24	787	0.005	0.030	1.6x10^{-12}	1.6x10^{-12}	2.70	TSG	2.49x10^{-10}	2.49x10^{-10}
FGFR3	7	359	0.002	0.019	1.03x10^{-5}	1.03x10^{-5}	3.278	Oncogene	0.001	0.001
EGFR	27	1408	0.007	0.019	1.08x10^{-5}	1.08x10^{-5}	1.382	Oncogene	0.001	0.001
PTEN	21	1742	0.005	0.012	9.96x10^{-5}	9.96x10^{-5}	1.381	TSG	0.008	0.007
NOTCH1	10	754	0.004	0.013	0.001	0.001	1.774	Oncogene/TSG	0.059	0.052
ERBB3	8	512	0.004	0.016	0.002	0.002	1.825	Oncogene	0.131	0.117
ERBB4	8	597	0.004	0.013	0.003	0.003	1.777	Oncogene	0.143	0.129

CDKN2A	11	1226	0.003	0.009	0.003	0.003	1.437	TSG	0.146	0.133
TSC1	6	364	0.004	0.016	0.005	0.005	1.972	TSG	0.186	0.171
LATS1	6	289	0.005	0.021	0.005	0.005	1.970	TSG	0.186	0.171
SMAD3	4	228	0.003	0.018	0.007	0.007	2.434	TSG	0.246	0.228
MYCN	2	149	0.001	0.013	0.015	0.015	3.442	Oncogene	0.496	0.463

p, p-value; q, p-adjusted value; OR, odds ratio.

Supplementary Table 8. AID composite mutations' enrichment analysis per residue in the MSKCC cohort. This is related to Fig. 4b, top significant residues only.

query_mutation	query_gene	n.samples_with_singleton_mutation_including_residue	n.samples_with_singleton_mutation_not_including_residue	n.samples_with_composite_mutation_including_residue	n.samples_with_composite_mutation_not_including_residue	OR	q.value.enriched
PIK3CA E726	PIK3CA	34	3642	53	69	81.92	2.59E-58
TP53 R342	TP53	176	12445	25	81	21.80	7.69E-20
TP53 R213	TP53	276	12345	28	78	16.05	4.24E-19
TP53 R196	TP53	189	12432	23	83	18.21	5.71E-17
EGFR A289	EGFR	47	1350	18	11	46.46	5.26E-16
PIK3CA R88	PIK3CA	51	3625	22	100	15.60	4.16E-14
FBXW7 S582	FBXW7	14	739	13	11	60.70	1.97E-12
PTEN R130	PTEN	142	1477	14	8	18.12	1.05E-07
KMT2D R4282	KMT2D	2	1377	6	22	181.80	1.94E-07
PBRM1 R710	PBRM1	5	666	5	2	299.64	8.06E-07
NOTCH1 R353	NOTCH1	1	710	4	6	426.19	1.54E-05
PIK3CA R93	PIK3CA	22	3654	9	113	13.20	4.56E-05
PIK3CA M1004	PIK3CA	10	3666	6	116	18.91	0.001
PIK3CA G1049	PIK3CA	36	3640	9	113	8.04	0.001
FGFR3 G380	FGFR3	6	356	4	4	55.88	0.002
ATM L2877	ATM	5	1043	3	10	60.62	0.009
FAT1 R2726	FAT1	3	937	3	14	64.83	0.009
SMARCA4 E882	SMARCA4	8	821	3	5	59.29	0.009
PIK3CA M1043	PIK3CA	52	3624	9	113	5.54	0.011
ERBB3 M91	ERBB3	5	501	3	5	56.96	0.012
TP53 Q136	TP53	32	12589	4	102	15.41	0.019
BAP1 S460	BAP1	4	542	2	1	234.17	0.024
CDKN2A R58	CDKN2A	74	1159	5	6	12.98	0.024
PIK3CA P449	PIK3CA	2	3674	3	119	46.06	0.024
ATRX R907	ATRX	4	1094	2	5	103.59	0.037

n, number; OR, odds ratio.

Supplementary Table 9. Number of singleton or composite mutations due to AID or not in the MSKCC cohort. This is related to Fig. 4C, data is organized according to the most frequent hotspots. Top 50 hotspots only.

hotspot	n.mutant	n.singletons	n.composites	n.singletons_AID	composite_AID	n.singletons_all	n.composites_all	p.singletons	p.composites	p.mutant	p.singletons_AID	p.composites_AID

KRAS G12	3988	3952	38	0	0	3952	38	0.0422	0.0130	0.0413	0.0000	0.0000
PIK3CA H1047	1029	904	129	0	0	904	129	0.0519	0.0572	0.0520	0.0000	0.0000
PIK3CA E545	920	839	85	0	0	839	85	0.0608	0.0863	0.0616	0.0000	0.0000
TP53 R273	815	764	51	0	0	764	51	0.0690	0.1038	0.0700	0.0000	0.0000
BRAF V600	764	753	11	0	0	753	11	0.0770	0.1075	0.0779	0.0000	0.0000
TP53 R248	760	716	45	0	0	716	45	0.0847	0.1229	0.0858	0.0000	0.0000
TP53 R175	671	634	39	0	0	634	39	0.0915	0.1363	0.0928	0.0000	0.0000
IDH1 R132	556	396	1	159	0	555	1	0.0957	0.1366	0.0985	0.0017	0.0000
PIK3CA E542	556	488	70	0	0	488	70	0.1009	0.1606	0.1043	0.0017	0.0000
NRAS Q61	478	475	3	0	0	475	3	0.1060	0.1616	0.1092	0.0017	0.0000
KRAS G13	384	373	11	0	0	373	11	0.1100	0.1654	0.1132	0.0017	0.0000
TP53 G245	368	269	20	76	3	345	23	0.1128	0.1723	0.1170	0.0025	0.0010
TP53 R282	327	298	30	0	0	298	30	0.1160	0.1825	0.1204	0.0025	0.0010
EGFR L858	326	270	58	0	0	270	58	0.1189	0.2024	0.1238	0.0025	0.0010
AKT1 E17	310	309	1	0	0	309	1	0.1222	0.2027	0.1270	0.0025	0.0010
KRAS Q61	282	273	9	0	0	273	9	0.1251	0.2058	0.1299	0.0025	0.0010
GNAS R201	247	242	5	0	0	242	5	0.1277	0.2075	0.1325	0.0025	0.0010
TP53 Y220	221	206	15	0	0	206	15	0.1299	0.2127	0.1348	0.0025	0.0010
SMAD4 R361	181	174	7	0	0	174	7	0.1318	0.2151	0.1367	0.0025	0.0010
TP53 H179	178	101	10	65	2	166	12	0.1328	0.2185	0.1385	0.0032	0.0017
PIK3CA N345	173	152	22	0	0	152	22	0.1345	0.2260	0.1403	0.0032	0.0017
TP53 C176	161	154	7	0	0	154	7	0.1361	0.2284	0.1420	0.0032	0.0017
CTNNB1 S37	150	144	6	0	0	144	6	0.1376	0.2305	0.1435	0.0032	0.0017
FGFR3 S249	147	131	17	0	0	131	17	0.1390	0.2363	0.1451	0.0032	0.0017
U2AF1 S34	144	144	1	0	0	144	1	0.1406	0.2366	0.1466	0.0032	0.0017
PTEN R130	142	72	16	49	5	121	21	0.1414	0.2421	0.1480	0.0037	0.0034
TP53	133	124	9	0	0	124	9	0.1427	0.2452	0.1494	0.0037	0.0034

P278												
TP53 H193	132	123	11	0	0	123	11	0.1440	0.2490	0.1508	0.0037	0.0034
ESR1 Y537	131	131	0	0	0	131	0	0.1454	0.2490	0.1521	0.0037	0.0034
TP53 R158	130	117	13	0	0	117	13	0.1466	0.2534	0.1535	0.0037	0.0034
TP53 R249	129	123	6	0	0	123	6	0.1480	0.2555	0.1548	0.0037	0.0034
FBXW7 R465	127	122	5	0	0	122	5	0.1493	0.2572	0.1561	0.0037	0.0034
TP53 S241	127	117	10	0	0	117	10	0.1505	0.2606	0.1574	0.0037	0.0034
ESR1 D538	123	123	0	0	0	123	0	0.1518	0.2606	0.1587	0.0037	0.0034
TP53 R280	123	106	17	0	0	106	17	0.1530	0.2664	0.1600	0.0037	0.0034
ERBB2 S310	121	105	17	0	0	105	17	0.1541	0.2723	0.1613	0.0037	0.0034
KRAS A146	118	25	0	92	1	117	1	0.1543	0.2723	0.1625	0.0047	0.0038
TP53 K132	118	111	7	0	0	111	7	0.1555	0.2747	0.1637	0.0047	0.0038
PIK3CA Q546	117	106	11	0	0	106	11	0.1567	0.2784	0.1649	0.0047	0.0038
FBXW7 R505	115	110	5	0	0	110	5	0.1578	0.2801	0.1661	0.0047	0.0038
TP53 M237	113	106	7	0	0	106	7	0.1590	0.2825	0.1673	0.0047	0.0038
TP53 V173	112	109	3	0	0	109	3	0.1601	0.2836	0.1684	0.0047	0.0038
TP53 C135	111	101	12	0	0	101	12	0.1612	0.2877	0.1696	0.0047	0.0038
TP53 G266	111	105	6	0	0	105	6	0.1623	0.2897	0.1707	0.0047	0.0038
TP53 E285	110	90	20	0	0	90	20	0.1633	0.2966	0.1719	0.0047	0.0038
CDKN2A H83	105	102	3	0	0	102	3	0.1644	0.2976	0.1730	0.0047	0.0038
CTNNB1 S45	104	100	4	0	0	100	4	0.1655	0.2990	0.1740	0.0047	0.0038
TP53 C238	104	98	6	0	0	98	6	0.1665	0.3010	0.1751	0.0047	0.0038
SF3B1 K700	101	101	0	0	0	101	0	0.1676	0.3010	0.1762	0.0047	0.0038
SPOP F133	101	70	0	28	3	98	3	0.1683	0.3010	0.1772	0.0050	0.0048

p, p-value; q, p-adjusted value; n, number.

Supplementary Table 10. Composite mutations analysis happening in the same cell (as function of clonality and allelic configuration) in the MSKCC cohort. This is related to Fig. 4d. Top enriched mutations only.

Hugo_Symbol	secondallele	firstallele	Tumor_Sample_Barcode	Role	same_cell_known	AICDA_mutation.1	AICDA_mutation.2	firstallele_N	secondallele_N	totalN	prop_samples_with_first_allele	prop_samples_with_first_allele_lwr	prop_samples_with_first_allele_upr	prop_samples_with_second_allele	prop_samples_with_second_allele_lwr	prop_samples_with_second_allele_upr
KRAS	KRAS G12	KRAS G13	P-0002475-T01-IM3	Oncogene	TRUE	NO	NO	384	3988	5136	0.075	0.068	0.082	0.776	0.765	0.788
KRAS	KRAS G12	KRAS G13	P-0033196-T01-IM6	Oncogene	TRUE	NO	NO	384	3988	5136	0.075	0.068	0.082	0.776	0.765	0.788
KRAS	KRAS G12	KRAS G13	P-0037482-T01-IM6	Oncogene	TRUE	NO	NO	384	3988	5136	0.075	0.068	0.082	0.776	0.765	0.788
KRAS	KRAS G13	KRAS G12	P-0016780-T01-IM6	Oncogene	TRUE	NO	NO	3988	384	5136	0.776	0.765	0.788	0.075	0.068	0.082
KRAS	KRAS G13	KRAS Q61	P-0022951-T02-IM6	Oncogene	TRUE	NO	NO	282	384	5136	0.055	0.049	0.061	0.075	0.068	0.082
KRAS	KRAS G13	KRAS Q61	P-0022951-T03-IM6	Oncogene	TRUE	NO	NO	282	384	5136	0.055	0.049	0.061	0.075	0.068	0.082
KRAS	KRAS Q61	KRAS G12	P-0027540-T01-IM6	Oncogene	TRUE	NO	NO	3988	282	5136	0.776	0.765	0.788	0.055	0.049	0.061
MAP3K1	MAP3K1 Truncating	MAP3K1 S1330	P-0006406-T01-IM5	TSG	TRUE	NO	NO	3	321	731	0.004	0.001	0.012	0.439	0.403	0.476
NOTCH1	NOTCH1 Truncating	NOTCH1 indel:356-359	P-0034481-T01-IM6	Oncogene/TSG	TRUE	NO	NO	19	233	881	0.022	0.013	0.033	0.264	0.236	0.295
NOTCH1	NOTCH1 indel:435-443	NOTCH1 Truncating	P-0001374-T01-IM3	Oncogene/TSG	TRUE	NO	NO	233	1	881	0.264	0.236	0.295	0.001	0.000	0.006
PIK3CA	PIK3CA A1066	PIK3CA E542	P-0017422-T01-IM6	Oncogene	TRUE	NO	NO	556	6	4502	0.124	0.114	0.133	0.001	0.000	0.003
PIK3CA	PIK3CA A1066	PIK3CA E545	P-0029639-T01-IM6	Oncogene	TRUE	NO	NO	920	6	4502	0.204	0.193	0.216	0.001	0.000	0.003
PIK3CA	PIK3CA C420	PIK3CA H1047	P-0023131-T01-IM6	Oncogene	TRUE	NO	NO	1029	78	4502	0.229	0.216	0.241	0.017	0.014	0.022
PIK3CA	PIK3CA E453	PIK3CA E542	P-0000397-T01-IM3	Oncogene	TRUE	NO	NO	556	63	4502	0.124	0.114	0.133	0.014	0.011	0.018
PIK3CA	PIK3CA E453	PIK3CA E542	P-0008694-T01-IM5	Oncogene	TRUE	NO	NO	556	63	4502	0.124	0.114	0.133	0.014	0.011	0.018
PIK3CA	PIK3CA E453	PIK3CA E542	P-0019593-T01-IM6	Oncogene	TRUE	NO	NO	556	63	4502	0.124	0.114	0.133	0.014	0.011	0.018
PIK3CA	PIK3CA E453	PIK3CA E545	P-0029938-T01-IM6	Oncogene	TRUE	NO	NO	920	63	4502	0.204	0.193	0.216	0.014	0.011	0.018
PIK3CA	PIK3CA E453	PIK3CA E545	P-0030392-T01-IM6	Oncogene	TRUE	NO	NO	920	63	4502	0.204	0.193	0.216	0.014	0.011	0.018
PIK3CA	PIK3CA E453	PIK3CA H1047	P-0004017-T01-IM5	Oncogene	TRUE	NO	NO	1029	63	4502	0.229	0.216	0.241	0.014	0.011	0.018
PIK3CA	PIK3CA E542	PIK3CA E545	P-0021660-T01-IM6	Oncogene	TRUE	NO	NO	920	556	4502	0.204	0.193	0.216	0.124	0.114	0.133
PIK3CA	PIK3CA E542	PIK3CA H1047	P-0004424-T01-IM5	Oncogene	TRUE	NO	NO	1029	556	4502	0.229	0.216	0.241	0.124	0.114	0.133
PIK3CA	PIK3CA E542	PIK3CA H1047	P-0014943-T01-IM6	Oncogene	TRUE	NO	NO	1029	556	4502	0.229	0.216	0.241	0.124	0.114	0.133
PIK3CA	PIK3CA E542	PIK3CA R93	P-0027608-T01-IM6	Oncogene	TRUE	YES	NO	35	556	4502	0.008	0.005	0.011	0.124	0.114	0.133
PIK3CA	PIK3CA E545	PIK3CA G1007	P-0036459-T01-IM6	Oncogene	TRUE	NO	YES	5	920	4502	0.001	0.000	0.003	0.204	0.193	0.216
PIK3CA	PIK3CA E545	PIK3CA G106	P-0023461-T02-IM6	Oncogene	TRUE	NO	NO	39	920	4502	0.009	0.006	0.012	0.204	0.193	0.216

PIK3CA	PIK3CA E545	PIK3CA K111	P-0024230-T01-IM6	Oncogene	TRUE	NO	NO	45	920	4502	0.010	0.007	0.013	0.204	0.193	0.216
PIK3CA	PIK3CA E545	PIK3CA M1043	P-0001631-T01-IM3	Oncogene	TRUE	NO	NO	71	920	4502	0.016	0.012	0.020	0.204	0.193	0.216
PIK3CA	PIK3CA E545	PIK3CA R93	P-0023566-T01-IM6	Oncogene	TRUE	NO	NO	35	920	4502	0.008	0.005	0.011	0.204	0.193	0.216
PIK3CA	PIK3CA E545	PIK3CA T1025	P-0017581-T01-IM5	Oncogene	TRUE	NO	NO	16	920	4502	0.004	0.002	0.006	0.204	0.193	0.216
PIK3CA	PIK3CA E726	PIK3CA E542	P-0006166-T01-IM5	Oncogene	TRUE	NO	YES	556	80	4502	0.124	0.114	0.133	0.018	0.014	0.022
PIK3CA	PIK3CA E726	PIK3CA E542	P-0013895-T03-IM6	Oncogene	TRUE	NO	YES	556	80	4502	0.124	0.114	0.133	0.018	0.014	0.022
PIK3CA	PIK3CA E726	PIK3CA E542	P-0035516-T01-IM6	Oncogene	TRUE	NO	YES	556	80	4502	0.124	0.114	0.133	0.018	0.014	0.022
PIK3CA	PIK3CA E726	PIK3CA E542	P-0037533-T01-IM6	Oncogene	TRUE	NO	YES	556	80	4502	0.124	0.114	0.133	0.018	0.014	0.022
PIK3CA	PIK3CA E726	PIK3CA E545	P-0005242-T02-IM6	Oncogene	TRUE	NO	YES	920	80	4502	0.204	0.193	0.216	0.018	0.014	0.022
PIK3CA	PIK3CA E726	PIK3CA E545	P-0005242-T03-IM6	Oncogene	TRUE	NO	YES	920	80	4502	0.204	0.193	0.216	0.018	0.014	0.022
PIK3CA	PIK3CA E726	PIK3CA E545	P-0011305-T01-IM5	Oncogene	TRUE	NO	YES	920	80	4502	0.204	0.193	0.216	0.018	0.014	0.022
PIK3CA	PIK3CA E726	PIK3CA H1047	P-0019458-T02-IM6	Oncogene	TRUE	YES	NO	1029	80	4502	0.229	0.216	0.241	0.018	0.014	0.022
PIK3CA	PIK3CA E726	PIK3CA N345	P-0028907-T01-IM6	Oncogene	TRUE	NO	YES	173	80	4502	0.038	0.033	0.044	0.018	0.014	0.022
PIK3CA	PIK3CA E726	PIK3CA V344	P-0006780-T01-IM5	Oncogene	TRUE	NO	YES	41	80	4502	0.009	0.007	0.012	0.018	0.014	0.022
PIK3CA	PIK3CA E78	PIK3CA E542	P-0005212-T01-IM5	Oncogene	TRUE	NO	NO	556	1	4502	0.124	0.114	0.133	0.000	0.000	0.001
PIK3CA	PIK3CA G106	PIK3CA R88	P-0013512-T01-IM5	Oncogene	FALSE	YES	NO	71	39	4502	0.016	0.012	0.020	0.009	0.006	0.012
PIK3CA	PIK3CA G364	PIK3CA H1047	P-0033968-T01-IM6	Oncogene	TRUE	NO	NO	1029	7	4502	0.229	0.216	0.241	0.002	0.001	0.003
PIK3CA	PIK3CA H1047	PIK3CA E542	P-0005154-T01-IM5	Oncogene	TRUE	NO	NO	556	1029	4502	0.124	0.114	0.133	0.229	0.216	0.241
PIK3CA	PIK3CA H1047	PIK3CA P539	P-0012635-T02-IM6	Oncogene	TRUE	NO	NO	25	1029	4502	0.006	0.004	0.008	0.229	0.216	0.241
PIK3CA	PIK3CA K111	PIK3CA H1047	P-0002841-T04-IM6	Oncogene	TRUE	NO	NO	1029	45	4502	0.229	0.216	0.241	0.010	0.007	0.013
PIK3CA	PIK3CA K111	PIK3CA H1047	P-0016066-T01-IM6	Oncogene	TRUE	NO	NO	1029	45	4502	0.229	0.216	0.241	0.010	0.007	0.013
PIK3CA	PIK3CA M1004	PIK3CA H1047	P-0035268-T01-IM6	Oncogene	TRUE	YES	NO	1029	16	4502	0.229	0.216	0.241	0.004	0.002	0.006
PIK3CA	PIK3CA M1004	PIK3CA Q546	P-0014515-T01-IM6	Oncogene	TRUE	NO	YES	117	16	4502	0.026	0.022	0.031	0.004	0.002	0.006
PIK3CA	PIK3CA M1043	PIK3CA E542	P-0031695-T01-IM6	Oncogene	TRUE	NO	YES	556	71	4502	0.124	0.114	0.133	0.016	0.012	0.020
PIK3CA	PIK3CA M1043	PIK3CA H1047	P-0020645-T01-IM6	Oncogene	TRUE	NO	NO	1029	71	4502	0.229	0.216	0.241	0.016	0.012	0.020
PIK3CA	PIK3CA N107	PIK3CA H1047	P-0018891-T01-IM6	Oncogene	TRUE	NO	NO	1029	8	4502	0.229	0.216	0.241	0.002	0.001	0.003
PIK3CA	PIK3CA N345	PIK3CA H1047	P-0001990-T01-IM3	Oncogene	TRUE	NO	NO	1029	173	4502	0.229	0.216	0.241	0.038	0.033	0.044
PIK3CA	PIK3CA P366	PIK3CA E726	P-0029666-T01-IM6	Oncogene	TRUE	NO	NO	80	4	4502	0.018	0.014	0.022	0.001	0.000	0.002
PIK3CA	PIK3CA P449	PIK3CA E542	P-0035155-T01-IM6	Oncogene	TRUE	YES	NO	556	6	4502	0.124	0.114	0.133	0.001	0.000	0.003
PIK3CA	PIK3CA P539	PIK3CA H1047	P-0014737-T01-IM6	Oncogene	TRUE	NO	NO	1029	25	4502	0.229	0.216	0.241	0.006	0.004	0.008
PIK3CA	PIK3CA P539	PIK3CA indel:108-111	P-0034473-T01-IM6	Oncogene	TRUE	NO	NO	1	25	4502	0.000	0.000	0.001	0.006	0.004	0.008

PIK3CA	PIK3CA Q546	PIK3CA K111	P-0033211-T01-IM6	Oncogene	TRUE	NO	NO	45	117	4502	0.010	0.007	0.013	0.026	0.022	0.031
PIK3CA	PIK3CA Q546	PIK3CA R88	P-0002633-T01-IM3	Oncogene	TRUE	YES	NO	71	117	4502	0.016	0.012	0.020	0.026	0.022	0.031
PIK3CA	PIK3CA R108	PIK3CA E542	P-0035155-T01-IM6	Oncogene	TRUE	NO	NO	556	29	4502	0.124	0.114	0.133	0.006	0.004	0.009
PIK3CA	PIK3CA R108	PIK3CA H1047	P-0030977-T01-IM6	Oncogene	TRUE	NO	NO	1029	29	4502	0.229	0.216	0.241	0.006	0.004	0.009
PIK3CA	PIK3CA R108	PIK3CA N345	P-0020383-T01-IM6	Oncogene	TRUE	NO	NO	173	29	4502	0.038	0.033	0.044	0.006	0.004	0.009
PIK3CA	PIK3CA R108	PIK3CA N345	P-0020383-T03-IM6	Oncogene	TRUE	NO	NO	173	29	4502	0.038	0.033	0.044	0.006	0.004	0.009
PIK3CA	PIK3CA R88	PIK3CA H1047	P-0005968-T01-IM5	Oncogene	TRUE	YES	NO	1029	71	4502	0.229	0.216	0.241	0.016	0.012	0.020
PIK3CA	PIK3CA R88	PIK3CA H1047	P-0027476-T01-IM6	Oncogene	TRUE	YES	NO	1029	71	4502	0.229	0.216	0.241	0.016	0.012	0.020
PIK3CA	PIK3CA R88	PIK3CA H1047	P-0031023-T01-IM6	Oncogene	TRUE	YES	NO	1029	71	4502	0.229	0.216	0.241	0.016	0.012	0.020
PIK3CA	PIK3CA R88	PIK3CA P539	P-0027476-T01-IM6	Oncogene	TRUE	YES	NO	25	71	4502	0.006	0.004	0.008	0.016	0.012	0.020
PIK3CA	PIK3CA R93	PIK3CA H1047	P-0001043-T01-IM3	Oncogene	TRUE	NO	NO	1029	35	4502	0.229	0.216	0.241	0.008	0.005	0.011
PIK3CA	PIK3CA T1025	PIK3CA E545	P-0006335-T02-IM6	Oncogene	TRUE	NO	NO	920	16	4502	0.204	0.193	0.216	0.004	0.002	0.006
PIK3CA	PIK3CA V344	PIK3CA E545	P-0031280-T01-IM6	Oncogene	TRUE	NO	NO	920	41	4502	0.204	0.193	0.216	0.009	0.007	0.012
PIK3CA	PIK3CA V344	PIK3CA H1047	P-0021148-T01-IM6	Oncogene	TRUE	NO	NO	1029	41	4502	0.229	0.216	0.241	0.009	0.007	0.012
PIK3CA	PIK3CA indel:10-18	PIK3CA E545	P-0019024-T01-IM6	Oncogene	TRUE	NO	NO	920	3	4502	0.204	0.193	0.216	0.001	0.000	0.002
PIK3CA	PIK3CA indel:10-19	PIK3CA E542	P-0033643-T01-IM6	Oncogene	TRUE	NO	NO	556	1	4502	0.124	0.114	0.133	0.000	0.000	0.001

Supplementary Table 11. Proportion of composite mutations happening in cis or trans per gene in the MSKCC cohort. This is related to Supplementary Figure 17f.

Gene	Role	Phase	Proportion	Frequency
TERT	Oncogene	cis_other	0.912	31
KMT2C	TSG	cis_other	0.846	11
KMT2D	TSG	cis_other	0.800	12
EGFR	Oncogene	cis_other	0.793	23
ARID1A	TSG	cis_other	0.783	18
PIK3CA	Oncogene	cis_other	0.618	21
CDKN2A	TSG	cis_other	0.474	9
TP53	TSG	cis_other	0.411	67
PIK3R1	TSG	cis_other	0.400	6
APC	TSG	cis_other	0.368	14
PTEN	TSG	cis_other	0.280	7
CDKN1A	TSG	cis_other	0.182	2
TERT	Oncogene	trans_other	0.088	3
KMT2C	TSG	trans_other	0.077	1
KMT2D	TSG	trans_other	0.000	NA
EGFR	Oncogene	trans_other	0.034	1
ARID1A	TSG	trans_other	0.130	3
PIK3CA	Oncogene	trans_other	0.118	4
CDKN2A	TSG	trans_other	0.368	7
TP53	TSG	trans_other	0.534	87
PIK3R1	TSG	trans_other	0.533	8
APC	TSG	trans_other	0.500	19
PTEN	TSG	trans_other	0.680	17
CDKN1A	TSG	trans_other	0.818	9
TERT	Oncogene	cis_AID	0.000	NA
KMT2C	TSG	cis_AID	0.077	1
KMT2D	TSG	cis_AID	0.133	2
EGFR	Oncogene	cis_AID	0.172	5
ARID1A	TSG	cis_AID	0.087	2
PIK3CA	Oncogene	cis_AID	0.265	9
CDKN2A	TSG	cis_AID	0.053	1
TP53	TSG	cis_AID	0.018	3
PIK3R1	TSG	cis_AID	0.067	1
APC	TSG	cis_AID	0.132	5
PTEN	TSG	cis_AID	0.000	NA
CDKN1A	TSG	cis_AID	0.000	NA
TERT	Oncogene	trans_AID	0.000	NA
KMT2C	TSG	trans_AID	0.000	NA
KMT2D	TSG	trans_AID	0.067	1
EGFR	Oncogene	trans_AID	0.000	NA
ARID1A	TSG	trans_AID	0.000	NA
PIK3CA	Oncogene	trans_AID	0.000	NA

CDKN2A	TSG	trans_AID	0.105	2
TP53	TSG	trans_AID	0.037	6
PIK3R1	TSG	trans_AID	0.000	NA
APC	TSG	trans_AID	0.000	NA
PTEN	TSG	trans_AID	0.040	1
CDKN1A	TSG	trans_AID	0.000	NA

Supplementary References

1. Rooney, M. S., Shukla, S. A., Wu, C. J., Getz, G. & Hacohen, N. Molecular and Genetic Properties of Tumors Associated with Local Immune Cytolytic Activity. *Cell* **160**, 48–61 (2015).
2. Shao, X. M. *et al.* High-Throughput Prediction of MHC Class I and II Neoantigens with MHCnuggets. *Cancer Immunol. Res.* **8**, 396–408 (2020).
3. Lawrence, M. S. *et al.* Mutational heterogeneity in cancer and the search for new cancer-associated genes. *Nature* **499**, 214–218 (2013).
4. Ginno, P. A., Lott, P. L., Christensen, H. C., Korf, I. & Chédin, F. R-Loop Formation Is a Distinctive Characteristic of Unmethylated Human CpG Island Promoters. *Mol. Cell* **45**, 814–825 (2012).
5. Pender, A. *et al.* Genome and Transcriptome Biomarkers of Response to Immune Checkpoint Inhibitors in Advanced Solid Tumors. *Clin. Cancer Res.* **27**, 202–212 (2021).
6. Samstein, R. M. *et al.* Tumor mutational load predicts survival after immunotherapy across multiple cancer types. *Nat. Genet.* **51**, 202–206 (2019).
7. Miao, D. *et al.* Genomic correlates of response to immune checkpoint blockade in microsatellite-stable solid tumors. *Nat. Genet.* **50**, 1271–1281 (2018).
8. Liu, D. *et al.* Integrative molecular and clinical modeling of clinical outcomes to PD1 blockade in patients with metastatic melanoma. *Nat. Med.* **25**, 1916–1927 (2019).
9. Hugo, W. *et al.* Genomic and Transcriptomic Features of Response to Anti-PD-1 Therapy in Metastatic Melanoma. *Cell* **165**, 35–44 (2016).
10. Braun, D. A. *et al.* Interplay of somatic alterations and immune infiltration modulates response to PD-1 blockade in advanced clear cell renal cell carcinoma. *Nat. Med.* **26**, 909–918 (2020).
11. Riaz, N. *et al.* Tumor and Microenvironment Evolution during Immunotherapy with Nivolumab. *Cell* **171**, 934-949.e16 (2017).
12. Amin, S. B. *et al.* Comparative Molecular Life History of Spontaneous Canine and

Human Gliomas. *Cancer Cell* **37**, 243-257.e7 (2020).

13. Gardner, H. L. *et al.* Canine osteosarcoma genome sequencing identifies recurrent mutations in DMD and the histone methyltransferase gene SETD2. *Commun. Biol.* **2**, 266 (2019).
14. Wong, K. *et al.* Cross-species genomic landscape comparison of human mucosal melanoma with canine oral and equine melanoma. *Nat. Commun.* **10**, 353 (2019).
15. Shinde, J. *et al.* Palimpsest: an R package for studying mutational and structural variant signatures along clonal evolution in cancer. *Bioinformatics* (2018) doi:10.1093/bioinformatics/bty388.
16. Carter, S. L. *et al.* Absolute quantification of somatic DNA alterations in human cancer. *Nat. Biotechnol.* **30**, 413–421 (2012).
17. Martincorena, I. *et al.* Universal Patterns of Selection in Cancer and Somatic Tissues. *Cell* **171**, 1029-1041.e21 (2017).
18. Mayakonda, A., Lin, D.-C., Assenov, Y., Plass, C. & Koeffler, H. P. Maftools: efficient and comprehensive analysis of somatic variants in cancer. *Genome Res.* **28**, 1747–1756 (2018).
19. Hui, W., Gel, Y. R. & Gastwirth, J. L. **lawstat**: An *R* Package for Law, Public Policy and Biostatistics. *J. Stat. Softw.* **28**, (2008).
20. Quinlan, A. R. & Hall, I. M. BEDTools: a flexible suite of utilities for comparing genomic features. *Bioinformatics* **26**, 841–842 (2010).
21. Schmidt, J. *et al.* Prediction of neo-epitope immunogenicity reveals TCR recognition determinants and provides insight into immunoediting. *Cell Rep. Med.* **2**, 100194 (2021).
22. Love, M. I., Huber, W. & Anders, S. Moderated estimation of fold change and dispersion for RNA-seq data with DESeq2. *Genome Biol.* **15**, 550 (2014).
23. Gu, Z., Eils, R. & Schlesner, M. Complex heatmaps reveal patterns and correlations in multidimensional genomic data. *Bioinformatics* (2016).
24. Gu, Z., Gu, L., Eils, R., Schlesner, M. & Brors, B. circlize implements and enhances circular visualization in R. *Bioinformatics* **30**, 2811–2812 (2014).

25. Yu, G., Wang, L.-G., Han, Y. & He, Q.-Y. clusterProfiler: an R Package for Comparing Biological Themes Among Gene Clusters. *OMICS J. Integr. Biol.* **16**, 284–287 (2012).
26. Terry M. Therneau & Patricia M. Grambsch. *Modeling Survival Data: Extending the Cox Model*. (Springer, 2000).
27. Hänzelmann, S., Castelo, R. & Guinney, J. GSVA: gene set variation analysis for microarray and RNA-Seq data. *BMC Bioinformatics* **14**, 7 (2013).
28. Gel, B. & Serra, E. karyoploteR: an R/Bioconductor package to plot customizable genomes displaying arbitrary data. *Bioinformatics* **33**, 3088–3090 (2017).
29. Blokzijl, F., Janssen, R., van Boxtel, R. & Cuppen, E. MutationalPatterns: comprehensive genome-wide analysis of mutational processes. *Genome Med.* **10**, 33 (2018).
30. Schwarzer, G., Carpenter, J. R. & Rücker, G. Fixed Effect and Random Effects Meta-Analysis. in *Meta-Analysis with R* (eds. Schwarzer, G., Carpenter, J. R. & Rücker, G.) 21–53 (Springer International Publishing, 2015). doi:10.1007/978-3-319-21416-0_2.
31. Cannataro, V. L., Gaffney, S. G. & Townsend, J. P. Effect Sizes of Somatic Mutations in Cancer. *JNCI J. Natl. Cancer Inst.* **110**, 1171–1177 (2018).
32. Akdemir, K. C. *et al.* Somatic mutation distributions in cancer genomes vary with three-dimensional chromatin structure. *Nat. Genet.* **52**, 1178–1188 (2020).
33. Hafemeister, C. & Satija, R. Normalization and variance stabilization of single-cell RNA-seq data using regularized negative binomial regression. *Genome Biol.* **20**, 296 (2019).
34. Bergstrom, E. N., Barnes, M., Martincorena, I. & Alexandrov, L. B. Generating realistic null hypothesis of cancer mutational landscapes using SigProfilerSimulator. *BMC Bioinformatics* **21**, 438 (2020).
